# Evaluation of a Partial Genome Screening of Two Asthma Susceptibility Regions Using Bayesian Network Based Bayesian Multilevel Analysis of Relevance

**DOI:** 10.1371/journal.pone.0033573

**Published:** 2012-03-14

**Authors:** Ildikó Ungvári, Gábor Hullám, Péter Antal, Petra Sz. Kiszel, András Gézsi, Éva Hadadi, Viktor Virág, Gergely Hajós, András Millinghoffer, Adrienne Nagy, András Kiss, Ágnes F. Semsei, Gergely Temesi, Béla Melegh, Péter Kisfali, Márta Széll, András Bikov, Gabriella Gálffy, Lilla Tamási, András Falus, Csaba Szalai

**Affiliations:** 1 Department of Genetics, Cell and Immunobiology, Semmelweis University, Budapest, Hungary; 2 Department of Measurement and Information Systems, University of Technology and Economics, Budapest, Hungary; 3 Heim Pal Children Hospital, Budapest, Hungary; 4 Inflammation Biology and Immunogenomics Research Group, Hungarian Academy of Sciences, Semmelweis University, Budapest, Hungary; 5 Csertex Research Laboratory, Budapest, Hungary; 6 Department of Medical Genetics, University of Pécs, Pécs, Hungary; 7 Dermatological Research Group, Hungarian Academy of Sciences, Szeged, Hungary; 8 Department of Pulmonology, Semmelweis University, Budapest, Hungary; Albert Einstein College of Medicine, United States of America

## Abstract

Genetic studies indicate high number of potential factors related to asthma. Based on earlier linkage analyses we selected the 11q13 and 14q22 asthma susceptibility regions, for which we designed a partial genome screening study using 145 SNPs in 1201 individuals (436 asthmatic children and 765 controls). The results were evaluated with traditional frequentist methods and we applied a new statistical method, called Bayesian network based Bayesian multilevel analysis of relevance (BN-BMLA). This method uses Bayesian network representation to provide detailed characterization of the relevance of factors, such as joint significance, the type of dependency, and multi-target aspects. We estimated posteriors for these relations within the Bayesian statistical framework, in order to estimate the posteriors whether a variable is directly relevant or its association is only mediated.

With frequentist methods one SNP (rs3751464 in the *FRMD6* gene) provided evidence for an association with asthma (OR = 1.43(1.2–1.8); p = 3×10^−4^). The possible role of the *FRMD6* gene in asthma was also confirmed in an animal model and human asthmatics.

In the BN-BMLA analysis altogether 5 SNPs in 4 genes were found relevant in connection with asthma phenotype: *PRPF19* on chromosome 11, and *FRMD6*, *PTGER2* and *PTGDR* on chromosome 14. In a subsequent step a partial dataset containing rhinitis and further clinical parameters was used, which allowed the analysis of relevance of SNPs for asthma and multiple targets. These analyses suggested that SNPs in the *AHNAK* and *MS4A2* genes were indirectly associated with asthma. This paper indicates that BN-BMLA explores the relevant factors more comprehensively than traditional statistical methods and extends the scope of strong relevance based methods to include partial relevance, global characterization of relevance and multi-target relevance.

## Introduction

Asthma is a multifactorial disease influenced by wide range of genetic and environmental factors. Despite the numerous genetic studies carried out to gain insight into its complexity, our understanding of the nature and consequences of the genetic variations remains limited. Several potentially important genomic regions have already been identified but the causal alterations which could be responsible for the observed linkages are sometimes not discovered or more frequently the results of the association studies cannot be confirmed by other studies. One of the disputed genomic regions in asthma is the chromosome 11q13 where positive linkage to atopy was first reported by Cookson et al. [1]. Subsequent association tests carried out on this region revealed the importance of the genes *MS4A2* (earlier FcεRI-β or β chain of the high-affinity receptor for IgE), *SCGB1A1* (earlier uteroglobin or Clara cell secretory protein (CC16)), glutathione S-transferase pi *(GSTP1)* and *GPR44* (earlier CRTH2) in asthma and asthma related phenotypes [1], [2], [3], [4].

Another frequently investigated asthma-related genomic region is the chromosome 14q22. Its attendance in the asthma development has been suggested by different whole-genome linkage studies [5]. Since then several genes have been shown to be involved in asthma-related processes in this region. These are genes for prostanoid transmembrane receptors, such as the prostaglandin-D2 receptor (*PTGDR*), the prostaglandin-E2 receptor (*PTGER2*) and the gene for galectin-3 (*LGALS3*) [6], [7], [8].

Despite the fact that associations of these regions and genes to asthma phenotypes were found earlier, none of them was confirmed subsequently by genome-wide association studies (GWAS) and meta-analyses [9], [10], [11].

This frequent phenomenon of genetic association studies has been explained by several theories, like the insufficient phenotypic descriptors (e.g. oversimplified case-control approach), insufficient observations (e.g. the negligence of rare genetic and epigenetic variants), ethnic differences between the study populations, confounding factors (population stratification and admixture), conventional design errors (small sample size, the multiple testing problem, small minor allele frequencies, curse of dimensionality, bias-variance dilemma, model complexity and significance, etc.), the inappropriate approach towards complex traits (i.e. disregarding the role of high-number of weak factors, gene-gene interactions, pathway-based interpretation), and the high redundancy of predictors (e.g. the discovery of non-causal, transitively associated descriptors) [12].

To overcome several of these limitations, probabilistic graphical models (PGMs) were proposed. Thanks to their ability to efficiently and accurately represent complex networks, PGMs represent powerful tools to dissect the genetic susceptibility of complex diseases. Bayesian networks are a popular class of PGMs, because they provide a clear, graphical semantics for representing a complete dependency-independency map of the domain. Therefore, the graphical representation presents a crucial advantage to allow the distinction between direct (causal) and indirect (due to LD) SNP-phenotype dependencies, thus ensuring a precise mapping of causal mutations. Additionally, BNs have the advantage of being able to efficiently deal with SNP–SNP interactions impacting the phenotype, a situation that is called epistasis.

Due to the high computational complexity and particularly because of the high sample (statistical) complexity, the learning of complete Bayesian network models is computationally prohibitive. To cope with complexity several ‘local’ approaches have emerged which limit their scope, and focus on the identification of strongly relevant variables, and possibly their interaction and causal structure. Thus, they omit a global and detailed characterization of relevance relations [13].

Other approaches apply a resampling scheme (i.e. bootstrap) or the Bayesian statistical framework to cope with the relatively small sample size, and to provide uncertainty and robustness measures for various model properties [14], [15], [16]. Although these methods have a much higher computational complexity than local causal identification approaches, their main advantage is that the modeling is not restricted, thus global characterization of the dependencies in the domain is possible. In fact, these approaches provide an overall characterization of the domain without the use of dedicated target variables [15].

Specifically within the Bayesian statistical framework the uncertainty of the validity of a discrete hypothesis given the observations is expressed by a probability, which is interpreted as an a posteriori belief in the hypothesis (e.g. above 0.5 it is more probable than not, see [17]). On the contrary, a p-value for a hypothesis in the frequentist approach is defined using the concept of repeatability and used indirectly through rejection to confirm a hypotheses.

Previously, we investigated the applicability of Bayesian networks using the Bayesian framework to learn the relevant variables with respect to a set of target variables. This could be seen as the Bayesian interpretation of the feature subset selection problem. We reported Monte Carlo methods to efficiently estimate posteriors over structural model properties, such as Markov blanket sets and Markov blanket graphs, which represent the strongly relevant variables and their interactions [18]. Based on these basic concepts we introduced a new statistical methodology, named Bayesian network based Bayesian multilevel analysis of relevance (BN-BMLA), which supports association analysis by estimating posteriors of strong relevance. In the context of genetic association studies, strongly relevant variables with respect to a phenotype represent the genetic and phenotypic factors that directly influence the phenotype (e.g. disease susceptibility). Strongly relevant variables statistically isolate the target variable from all other variables. However, the standard concept of pairwise association is not identical with the concept of strong relevance. First, if the dependency of a SNP to the phenotype is indirect due to LD with the causal SNP, then it is (transitively) associated but not strongly relevant to the phenotype. Second, if a SNP has no main effect on the phenotype, but has an epistatic effect along with an other factor, then this SNP is not associated (according to the prevailing terminology), but strongly relevant to the phenotype (i.e. it is in pure interaction with the phenotype). Therefore, strong relevance indicates either a strong, direct association or a pure interactionist relevance. See Figure S1 for further comparison of the concept of association and strong relevance.

Additionally, posteriors for strong relevance and partial strong relevance for subsets of variables can also be computed. The concept of partial strong relevance, that a set of *k* predictors are strongly relevant, is particularly useful, because it defines a hierarchical, embedded hypotheses space with varying complexity. For a given domain and sample size the posteriors over k-(strong)-relevance can be used to determine the sufficiency of the data and these posteriors can also be used to investigate statistical interactions [19]. These model properties with varying complexities support the overview, the post hoc interpretation, and the offline meta-analysis of weakly significant results [20]. We tested the BN-BMLA method in a case-control setup using artificial datasets for identifying interactions and conditional relevance and it was proven to be superior over other multivariate methods using conditional models designed to detect associations between genotypic variables and the target variable [21].

In this paper we report results of the BN-BMLA statistical method on real-world genotype data from our partial genome screenings of the 11q13 and 14q22 regions in asthmatic patients and controls. We apply and demonstrate the unique ability of BN-BMLA to provide (1) a complete overview about partial multivariate relevance (i.e., posteriors of partial relevance of *k* predictors for varying *k*), (2) an overview about various types of dependencies between a predictor and a target based on global characterization (i.e., posteriors that a predictor is directly or transitively dependent, or is in pure interaction), and (3) a joint characterization of relevance for multiple target variables (i.e. to model the dependencies of the targets). We compare these results with results from traditional frequentist methods, and discuss the relevant candidate genes and their interactions from the aspect of association with asthma in our population. Among the newly identified genes *FRMD6* showed the strongest association with asthma, and we confirmed the possible role of this gene in the disease in an animal model and in human asthmatics.

## Methods

### Subjects

The study population comprised 1201 unrelated individuals of Hungarian (Caucasian) population. Approximately, 5% of tested subjects were probably of Gypsy origin (estimation based on state population statistics).

Four hundred and thirty six asthmatic children, age 3–18 were recruited to the study. All the asthmatic children had specialist physician-diagnosed asthma with the following characteristics: (1) recurrent breathlessness and expiratory dyspnea requiring treatment; (2) physician diagnosed wheeze; (3) reversibility of the wheezing and dyspnea by bronchodilator treatment measured as forced expiratory volume 1 s (FEV_1_) by a spirometer. All the asthmatics (or their parents) were instructed to record their symptoms accurately for 2 weeks, the treatment, and the peak expiratory flow (PEF) twice daily (in the evening and in the morning). PEF 100% was determined by calculation from the personal best value and the expected value according to the height of the patient.

If the patient is younger than 5 years old, the determination of lung function tests (PEF or FEV_1_) is usually not possible. In that case the diagnosis and classification of the disease were made according to the frequency and severity of other symptoms.

The treatment of the patients remained unchanged before the blood was drawn. None of the asthmatic had experienced an exacerbation or a respiratory infection for at least 4 weeks as indicated by increased symptoms.

The control children were randomly selected from outpatients from the Orthopaedic Department in the Budai Children's Hospital and from the Urological Department of Heim Pal Hospital, Budapest. Children in the control group had mild musculoskeletal alterations (like pes planus or scoliosis), phimosis, or other small urogenital problems, showed no symptoms of asthma and required no medication. The adult controls were blood donors without asthma, and according to questionnaires they had not experienced asthma symptoms earlier. The control group comprised 765 subjects (mean age: 19±12 years, 405 males/360 females).

In this paper we also demonstrate the ability of the BN-BMLA method to predict various types of dependencies from small amount of available data. For this purpose we applied the method to three embedded datasets: (1) the asthma status was known in all cases (1201 subjects, “A” dataset); (2) in all asthmatics (436) and in 664 controls, altogether in 1100 cases the status of rhinitis was also known (“RA” dataset). Rhinitis was diagnosed in 278 asthmatics (64.0%), and in 233 controls (35.1%). Rhinitis was defined by troublesome sneezing or blocked or runny nose severely affecting the well being of the patient in periods without common cold or flu. Only those subjects were involved in this dataset whose rhinitis status was verified by specialists; (3) in 200 children (106 asthmatics and 94 controls) the status of rhinitis and the serum levels of total IgE and eosinophil were known (“CLI” dataset, Table 1).

**Table 1:** Some characteristics of the study subjects belonging to the CLI dataset.

Clinical and biological characteristics	Asthmatics	Controls
Number	106	94
Age, years	11.5±6.1	13.0±4.2
Gender, male/female	63/43	55/39
Rhinitis (%)	66.0	31.9
Total IgE kU/l	260.4±111	94.6±63
Eosinophil cell count 10^6^/ml	0.34±0.25	0.14±0.07

For the measurement of gene expression level in induced sputum, 31 adults between 19 to 61 years old were enrolled in the study, but 10 of them were excluded from the analysis because the qualities of the sputum samples were not appropriate. The study population comprised of 12 asthmatic patients (5 males and 7 females; mean age 36.3±13.0 years) and 9 controls (4 males and 5 females; mean age 29.3±4.9 years). The 12 asthmatic patients had mild atopic stable asthma, no other lung diseases and no lower respiratory tract infection. Patients were required to have FEV1 of greater than 70% of predicted, baseline methacholine PC20 (the provocative concentration of methacholine causing a 20% fall in FEV1) of less than 16 mg/ml. The 9 healthy volunteers were recruited from the staff and students of the participating Hungarian universities. They gave no history of respiratory diseases, had a FEV1/FVC>80% and normal methacholine airway responsiveness (PC20>16 mg/ml).The included groups did not differ statistically regarding age, sex, smoking status and allergy.

The study was conducted according to the principles expressed in the Declaration of Helsinki, and approved by the Ethics Committee of the Hungarian Medical Research Council (ETT TUKEB; http://www.ett.hu/tukeb/tukeb.htm). Written informed consent was obtained from all patients or from the parents or guardians of the minors involved in the study.

### Laboratory analysis

Genomic DNA was extracted from peripheral blood using DNA blood isolation midi kit (Qiagen, Valencia, CA).

Multiplex PCR and SNP genotyping for 144 SNPs were performed using 48- and 12plex genotyping assays on GenomeLab SNPstream genotyping platform (Beckman Coulter Inc. Fullerton, CA) using single-base primer extension technology. In addition, SNP rs545659 was genotyped using TaqMan SNP Genotyping Assay (Applied Biosystems, Warrington, UK) on an Applied Biosystems 7900 Real Time PCR System as per instructions of the manufacturer. Some samples were genotyped in duplicate in the same and in different plates. Only those SNPs where the genotyping call rate was >90% were included in the analysis. Some SNPs genotyped on GenomeLab SNPstream genotyping platform were also genotyped using TaqMan SNP Genotyping Assays. No difference between the results of the two methods was revealed.

Total serum IgE levels were determined by 3gAllergy blood tests in Immulite 2000 Immunoassay System (Siemens Healthcare Diagnostics; Deerfield, IL USA).

The eosinophil cell counts were measured by Coulter MAXM Analyser.

RNA was isolated with the Qiagen Mini RNeasy kit (Maryland, USA). RNA was transcribed to cDNA with the High Capacity cDNA Reverse Transcription Kit (Applied Biosystems, Foster City, CA). Real-time quantitative PCR was performed for the genes found to be relevant in asthma in our analyses (*FRMD6*, *PTGDR*, *PTGER2*, *MS4A2*, *AHNAK*, *PRPF19*, *TXNDC16*) and β-actin using an ABI 7900HT Fast Real-Time PCR System (Applied Biosystems, Foster City, CA) and TaqMan Gene Expression Assays. Relative gene expression was determined by the comparative CT method (ddCT) with β-actin as the endogenous control.

### Sputum induction and analysis

Sputum was collected from 20 asthmatic and 11 healthy volunteers. The participants inhaled 4.5% saline solution generated by a De Vilbiss Nebulizer (Ultra-NebTm 2000 model 200HI) for 5 minutes after pre-treatment with 400 µg of inhaled salbutamol. Induction was performed three times and the pulmonary function was measured each time after the sputum induction. All portions that macroscopically appeared free of salivary contamination were selected. Samples were diluted with phosphate buffered saline containing 0.1% dithiotreitol (Sigma, St Louis, MO, USA), portions were agitated with a vortex and placed on a bench rocker for 30 minutes. Samples were filtered through a 40 µm Falcon cell strainer, and centrifuged at 1500 rpm for 10 minutes. The cell pellet was resuspended in 1 ml PBS and viability (Trypan blue exclusion method) was determined using Burker chamber. After differential cell count, cells were stocked on lyses buffer at −80°C until use.

### SNP selection

SNPs in chromosome region 11q12.2-q13.1 and 14q22.1-22.3 were selected using HapMap data analyzed with Haploview 4.1 (http://www.hapmap.org). Our gene coverage pipeline included tag SNPs for all of the detected haplo blocks with minor allele frequency >0.05 and selected at an *r* ^2^ of 0.8 for the CEU population. In addition to using LD criteria, we also added SNPs based on spacing across the region and their estimated functionality. In this manner we selected 145 SNPs in the given regions of Chr 11 and Chr 14 (68 and 77 SNPs, respectively) for genotyping. See Table S1 for detailed information on the examined SNPs.

### Statistical methods

#### Frequentist methods

Allele frequencies were calculated by allele counting. Data were analyzed using MedCalc 5.0 and SPSS 11.5 programs. Hardy-Weinberg equilibrium was tested by using a χ^2^ goodness-of-fit test. χ^2^ test was used to test for differences in allele distribution between the groups. Logistic regression adjusted for age and sex was applied to assess the effect of the genetic background to dichotomous clinical characteristics. Confidence intervals were calculated at the 95 percent level. Estimated haplotype frequency was calculated by Haploview 4.1: http://www.broad.mit.edu/mpg/haploview/. Haplotype-specific ORs were estimated using conditional logistic regression to model the log odds of disease as a function of the individuals' haplotype probabilities. Normalized gene expression levels were compared by t-test. Power analysis was carried out by genetic power calculator (http://pngu.mgh.harvard.edu/~purcell/gpc [22].

In order to facilitate a multivariate based frequentist analysis we applied multivariate logistic regression and multifactor dimensionality reduction (MDR). MDR is a nonparametric and genetic model-free data mining method for detecting nonlinear interactions among discrete genetic and environmental variables [23].

#### BN-BMLA method

A Bayesian Network (BN) is a directed acyclic graph (DAG) that represents a joint probability distribution of a set of random variables{X_1_, X_2_, …, X_n_}. These refer to the observed or measured factors (e.g. SNPs, clinical parameters) from a specific domain. A node of the graph represents a variable and an edge between two nodes represents a direct dependency between the variables.

Learning a BN structure (i.e. the dependence relations of the variables) is finding a DAG that best describes the dataset. In most cases, where the amount of available data is modest relative to the number of variables there are likely to be many models that have non-negligible posteriors. However, there might be certain structural features, e.g. the presence of an edge, that we can extract reliably. A central structural feature is related to the concept of strong relevance of a single variable or a set of variables. For the definition of Markov blanket sets (MBS), see [24], for strong relevance, see [25], for Markov blanket membership (MBM), see [26] and for their relation, see [27].

With *Bayesian learning* we can estimate the strength with which the data indicates the presence of a certain feature by estimating its a posteriori probability (Eq. (1)):
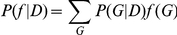
where *G* represents a BN structure, *D* is the dataset, and *f*(*G*) is 1 if the feature holds in *G* and 0 otherwise (for an overview, see [28]. For example, we refer to *p*(MBS = *s|D*) as the MBS posterior of *s*.

#### Bayesian learning

To calculate the first term of the summation in Eq.(1) we use Bayes rule, and we have that
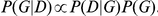
The term *P*(*D|G*) is the marginal likelihood of the data given structure *G*, and the term *P*(*G*) is the prior probability of a structure *G*. We used uniform prior over structures in our experiments.

Assuming that the dataset *D* is complete (i.e., there are no missing values), the variables are multinomial with a Dirichlet parameter prior for every possible instantiations of their parents, and the prior *P*(*G*) satisfies parameter independence, parameter modularity, and structure modularity then the marginal likelihood has an efficiently computable closed form [29].

#### Bayesian model averaging

As it is mentioned before, our goal was to compute the posterior probability of a feature (i.e., Eq.(1)). Because the number of BN structures is super-exponential in the number of random variables in the domain, exact summation of all possible structures *G* is computationally intractable (see [29], [30]. We used Metropolis Coupled Markov Chain Monte Carlo (MC^3^) [31] methods for the approximation of Eq.(1). We defined parallel Markov chains over the space of DAGs, whose stationary distribution were the posterior distribution *P*(*G|D*). We then generated samples by doing random walks in these chains, and used them to estimate Eq. (1) (for convergence and confidence diagnostics, see [32].

Each step in a Markov Chain corresponds to local transformations of the DAG, called operators [33], [34]. Following Castello, we used three operators: (1) adding an edge to the DAG, if it does not violate the acyclic constraint, (2) reversing an edge, if it does not violate the acyclic constraint and (3) removing an edge from the DAG. The probability of the operator selection was uniform.

We ran the MCMC sampler with a burn-in period of 10^6^ steps and then collected 5×10^6^ samples. We restricted the space of the possible structures limiting the number of parents per node to 8.

We computed a posteriori probabilities for structural features summarized in Table 2.

**Table 2:** Structural features that indicate different dependence types between the variables.

Relation	Abbreviation	Graphical
**Pairwise features**		
Direct causal relevance	DCR(X,Y)	There is an edge between X and Y
Transitive causal relevance	TCR(X,Y)	There is directed path between X and Y
Confounded relevance	ConfR(X,Y)	X and Y have common ancestor
Association	A	DCR or TCR or ConfR
Pure interactionist relevance	PIR(X,Y)	X and Y have common child
Strong relevance	SR(X,Y)	PIR or DCR
**Relevance of variable sets**		
Strong relevance	MBS(Y)	The set consisting of Y's parents, its children, and the other parents of its children (the Markov Blanket Set of Y)
**Relevance for multiple target variables**		
Direct relation to one or more targets	EdgeToAny(X, **Y**)	There is an edge between X and **Y**
Strong relevance to one or more targets	SR(X, **Y**)	There is an edge between X and **Y** or X and **Y** have common child

**Figure 1:**
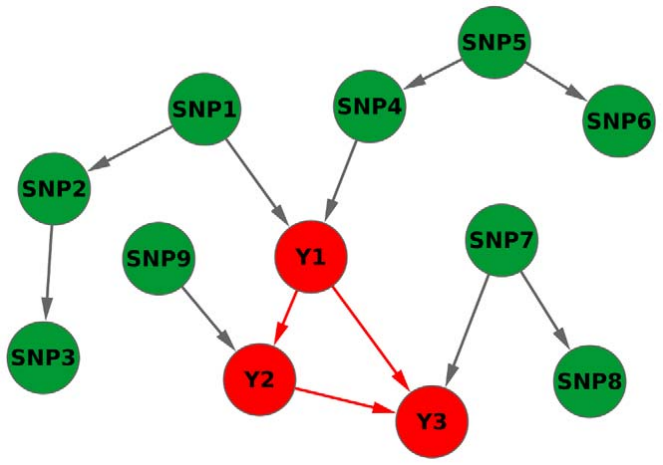
Illustration of different dependency types between variables in a Bayesian Network structure. *Pairwise relevance relations*: Direct causal relevance (e.g., Y1 and SNP1 have common edge), Transitive causal relevance (e.g., there are two directed paths between Y3 and SNP5), Confounded relevance (e.g., Y2 and SNP3 have a common ancestor SNP1), Association (e.g., Y1 and SNP1, because SNP1 is directly related to Y1; Y3 and SNP5, because SNP5 is transitively related to Y3; Y2 and SNP3, because they are in a confounded relation), Pure interactionist relevance (e.g., Y1 and SNP7 have common child), Strong relevance (e.g., Y1 and SNP1, because SNP1 is directly related to Y1; Y1 and SNP7, because they are in pure interaction). *Relevance of variable sets*: Strong relevance (e.g., the variable set consisting of Y2's parents, its children, and the other parents of its children is {Y1, SNP9, Y3, SNP7}). *Relevance for multiple target variables*: Strong relevance to one or more targets (e.g., the variable set consisting of {Y1,Y2,Y3}'s parents, its children, and the other parents of its children is {SNP1, SNP4, SNP7, SNP9}). *Red nodes*: potential target variables, *Green nodes*: SNP variables.

See Figure 1 for a graphical example.

In a post-processing step, we introduced the concept of *k*-MBS as the *k* sized subset of the strongly relevant variables [19]. The a posteriori probability of the sub-relevance of a *k*-sized set *s* is
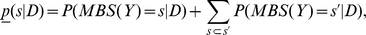
where the terms are the exact MBS posterior of the set *s* and the MBS posterior for all its superset.

The concept of *k*-MBS was motivated by the observation that typically the most probable strongly relevant variable sets often share a significant common part. This partial multivariate approach provides an intermediate and scalable complexity for the analysis. Note that the cardinality of the *k*-MBS features is O(n^k^) and the aggregation involves optionally 2^n^ MBS subsets, which is limited by the number of the DAG structures visited in the MCMC run, i.e. by the number of steps, which is typically 5×10^6^, resulting in 10^4^ different DAGs and MBS sets.

Posterior values are between 0 and 1, where 0 indicates no relevance, 1 indicates 100% relevance between a predictor and a target variable. In our study we consider a variable relevant when its posterior is greater than or equal 0.5.

## Results

### Frequentist analysis

From the 145 genotyped SNPs 5 SNPs were monomorphic (MAF = 0), 5 deviated from Hardy-Weinberg equilibrium in controls (p<0.005) and 33 had poor genotyping results (poor genotype clusters or low call rates) and were not considered for further analyses leaving 102 SNPs (59 in 14q22.1-22.3 and 43 in 11q12.2-q13.1) for frequentist and BN-BMLA analyses.

Table S2 shows the minor allele and genotype frequencies in asthmatic and control patients. Table S3 presents the statistical evaluation for association of SNPs with asthma at allele and genotype levels.

When allele frequencies were considered, one SNP (rs3751464) in the *FRMD6* gene provided an evidence for an association with asthma (OR = 1.43 (1.18–1.75); p = 3×10^−4^). Only this SNP could withstand the correction for multiple testing. Because 102 SNPs were considered, the Bonferroni corrected significance level was 5×10^−4^. This result is compatible with a power analysis which showed that the power of frequentist statistical tests are less than 0.2 for OR below 1.3 at sample size of 1200 (complete dataset).

When the genotype frequencies were considered, the CC genotype of the SNP rs17831682 provided strong evidence for an association (P = 3.9×10^−4^), indicating a recessive model. This SNP is located in the 3′ UTR of the *PTGDR* gene and considered as an exonic splicing enhancer (Genecards). Haplotypes were constructed from the investigated SNPs (Figure S2). The permutation based association test of Haploview indicated two haplotypes consisting of two SNPs (rs3751464 and rs17666653) in the *FRMD6* gene for association with asthma. The TC haplotype increased the susceptibility to asthma (OR = 1.41 (1.07–1.87); permutation p value 0.048), while the CC haplotype reduced it (OR = 0.73 (0.57–0.92); permutation p = 0.02) constructed from rs3751464 and rs17666653, respectively. The haplotype frequencies were for TC: 0.191 and 0.251 and for CC 0.606 and 0.528, in controls and cases, respectively. The rs17666653 is located in intron 4 of the *FRMD6* gene.

### Bayesian network based Bayesian multilevel analysis of relevance

#### Sufficiency of the data

In the Bayesian context we first investigated the sufficiency of the sample size of all datasets (A: 1201, RA: 1100, CLI: 200) for performing a full-scale multivariate analysis to identify the set of relevant variables. This confirmed the necessity of a partial multivariate approach, because there was neither a dominant set nor a set of highly significant sets for the A/RA and especially not for the CLI datasets (Figure S3).

#### Strong univariate relevance

The most relevant SNPs and genes (i.e. with high posteriors for strong relevance with respect to asthma) according to the BN-BMLA are presented in Table 3. In the last column the p values and odds ratios calculated with logistic regression are also shown. Altogether 5 SNPs in 4 genes were found relevant in connection with asthma phenotype: *PRPF19* on chromosome 11, and *FRMD6*, *PTGER2* and *PTGDR* on chromosome 14. The SNP rs7928208 in the gene *PRPF19* is also associated with early childhood asthma (in case of children under 6 years).

**Table 3:** Properties of the most relevant SNPs including posteriors for strong relevance with respect to asthma.

SNP	Gene	Influenced trait	Localization	Posterior probability*	OR (95%CI)	P-value
rs7928208	*PRPF19*	asthma	11q12.2	0.73	1.78 (1.08–2.95)	0.02
rs3751464	*FRMD6*	asthma	14q22.1	0.86	1.43 (1.18–1.75)	0.0003
rs17831682	*PTGDR*	asthma	14q22.1	0.51	1.38 (1.00–1.80)	0.05
rs708502	*PTGER2*	asthma	14q22	0.85	0.98 (0.77–1.25)	0.9
rs17197	*PTGER2*	asthma	14q22	0.82	1.05 (0.82–1.33)	0.7
rs7928208	*PRPF19*	asthma at 6 years	11q12.2	0.91	4.38 (1.89–10.14)	0.0005

* Strong relevance.

Table 4 summarizes the posterior probability of strong relevance for the most relevant SNPs for asthma and for multiple targets, in case of RA and CLI data sets. By multiple targets we mean rhinitis+asthma (RA data set) and IgE level+eosinophil level+rhinitis+asthma (CLI data set) respectively. In case of the RA data set two SNPs in the *AHNAK* gene gave the strongest correlation with the phenotype asthma+rhinitis. Interestingly, a SNP in the most studied gene of the 11q13 region, the *MS4A2*, the gene for the high affinity IgE receptor β subunit showed an association when the CLI data was considered.

**Table 4:** The posterior probability of strong relevance for the most relevant SNPs in case of RA and CLI data.

		RA*		CLI**	
Gene	SNP	Asthma	Multitarget	Asthma	Multitarget
*AHNAK*	rs11231128	0.801	0.826	0.394	0.684
*AHNAK*	rs11827029	0.798	0.810	0.426	0.774
*FRMD6*	rs3751464	0.324	0.280	0.039	0.267
*MS4A2*	rs569108	0.098	0.151	0.441	0.787
*PRPF19*	rs7928208	0.801	0.781	0.145	0.449
*PTGDR*	rs17831675	0.371	0.365	0.424	0.669
*PTGDR*	rs17831682	0.542	0.598	0.466	0.703
*PTGER2*	rs12587410	0.367	0.348	0.772	0.922
*PTGER2*	rs17197	0.380	0.371	0.596	0.881
*PTGER2*	rs708498	0.248	0.206	0.557	0.688
*TXNDC16*	rs1565970	0.754	0.717	0.235	0.484

* *RA – Asthma*: RA dataset, Asthma as target.

*RA – Multitarget*: RA dataset, Asthma and Rhinitis as targets.

** *CLI – Asthma*: CLI dataset, Asthma as target.

*CLI – Multitarget*: CLI dataset, IgE level-Eosinophil level-Rhinitis-Asthma as targets.

#### Partial multivariate relevance

**Figure 2:**
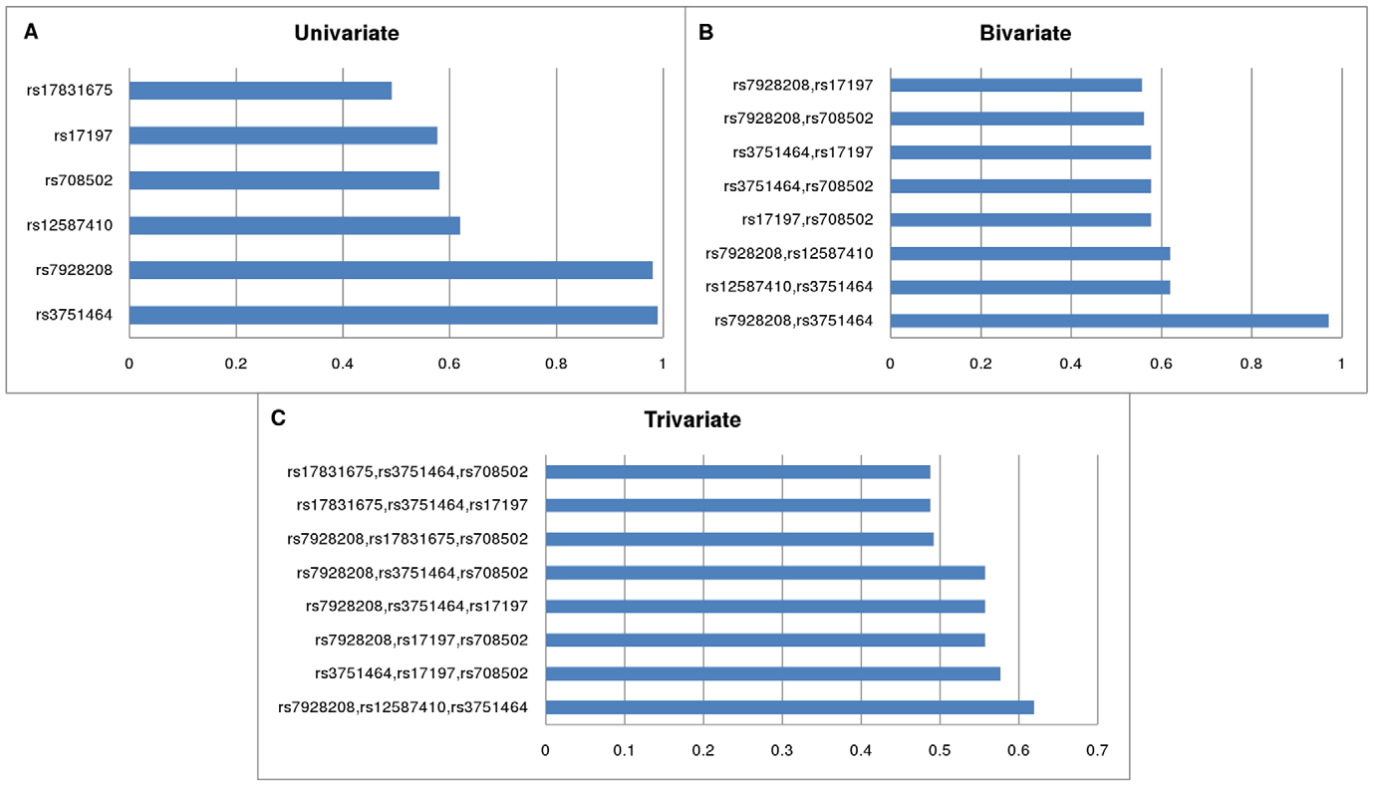
The most probable univariate (MBM), bivariate (2-MBS), trivariate (3-MBS) subsets of variables (Asthma dataset). Relevant SNPs having high or moderately high posteriors, i.e. high probability of being a member of the Markov blanket (MBM) of the target variable *Asthma* (**A**). Relevant SNP sets of size 2 (**B**); and of size 3 (**C**) indicating partial strong relevance. 2-MBS and 3-MBS denote the k = 2 and k = 3 sized subsets of Markov blanket sets. A high *k*-MBS posterior of a set of SNPs indicates their joint relevance and possible interactions between the SNPs.

The multivariate analysis of strong relevance (i.e. the posterior probabilities of Markov blanket sets with asthma as a target, for details see Methods) indicated a very flat posterior distribution. This means that there are several possible strongly relevant sets with low posteriors instead of a dominant set with a high posterior. On the other hand, the aggregation of the multivariate results into univariate conclusions, i.e. strong univariate relevance (described previously) indicated that model averaging can unhinge significant results. Furthermore, it is also possible to aggregate multivariate results into partial multivariate results (i.e. *k*-sized subsets, as detailed in the Methods section),

This allows the investigation of SNP-SNP interactions, because a subset with a high posterior can indicate that these SNPs have a joint effect on the susceptibility of asthma (for a detailed investigation of interactions, see [19]. Results for partial relevance are systematically presented in Figure 2 showing the highest posteriors for various subset sizes (*k*). In this case the “A” dataset was evaluated using asthma as a target variable. The high posteriors for *k* = 1,2,3,4 indicate that the data is sufficient to infer that these variables are jointly strongly relevant, but above that level (*k*≥5) as shown in Figure S4 the multivariate results are weakly significant.

We applied the MDR method on the “A” dataset for model sizes (k = 1,2,3,4) using an exhaustive evaluation. The results confirmed the strong significance of rs3751464 of *FRMD6*, because it was the most frequent part of the models. However, the difference in model scores within a certain model size was low. The range of scores for the 20 highest scoring models containing 1, 2, 3, and 4 variables were 0.548–0.521, 0.561–0.556, 0.592–0.586, and 0.639–0.630 respectively. Furthermore, the 20 highest scoring models of each size (1–4) contained 51 different SNPs altogether, which also confirms the low power of MDR in case of this data set. In contrast to the approach followed in BN-BMLA, model averaging was not possible because of the frequentist nature of the MDR score (compare with Eq.1.). Manual investigation of MDR results indicated that the following SNPs were often parts of the highly significant models: rs3751464 in *FRMD6*, rs17831675 and rs17831682 in *PTGDR*, rs708502 and rs708486 in *PTGER2*.

We also performed logistic regression analysis on the “A” data set with SPSS using PIN = 0.05 as the probability threshold for variable entry, and POUT = 0.1 as the threshold of removing a variable from the model. The forward variable selection method confirmed the strong significance of rs3751464 in *FRMD6* (exp(B) = 1.51, C.I. 95%: [1.23–1.86], p-value<0.001) and rs7928208 in *PRPF19* (exp(B) = 0.56, C.I. 95%: [0.33–0.92], p-value = 0.024). The logistic regression with backward variable selection method was also applied for the 10 most significant variables and their interactions indicated by BN-BMLA. It confirmed the strong significance of rs3751464 (*FRMD6*), rs708502 (*PTGER2*), and rs7928208 (*PRPF19*) and their interactions (see detailed analysis in Gene-gene interactions Section).

#### Gene-gene interactions

The k-MBS concept from BN-BMLA represents the joint relevance of k variables. However, their effects can be linear or non-linear at some scale, which is indicated by the absence or presence of interaction terms in appropriate statistical models. Furthermore, (non-linear) interactions can be epistatic, i.e. a SNP has no main effect on the phenotype, but has an effect along with an other factor. As can be seen in the last column of Table 3, the distribution of the two SNPs in the *PTGER2* gene does not differ between the asthmatic and control groups. It implies that these SNPs do not influence asthma risk alone, only in interaction with other variables, in this case with other SNPs. Table 5 presents the most probable gene-gene (SNP-SNP) joint relevances and interactions for asthma as a target variable. In this evaluation significant interactions between 2 and 3 SNPs were revealed. The table shows the p-values and the corresponding odds ratios for the interaction terms in logistic regression calculated with SPSS using the enter method. The most relevant interactions include intrachromosomal (e.g. rs708502 in *PTGER2* and rs3751464 in *FRMD6*) and interchromosomal interactions: rs7928208 in *PRPF19* (chr. 11) with rs708502 in *PTGER2* (chr. 14) and rs7928208 in *PRPF19* (chr. 11) with rs3751464 in *FRMD6* (chr. 14). Interestingly, the joint interaction of these three variables is also significant (exp(B) = 0.72, C.I. 95%: [0.58–0.89], p-value = 0.002) which is indicated by the logistic regression backward method (described in the previous section). According to these results, the most significant SNP in this study is the rs3751464 in the *FRMD6* gene. It influences the asthma risk both alone and in interactions with other SNPs in *PRPF19* and *PTGER2* genes. In all the interactions with the minor rs3751464 TT genotype significantly increase the asthma risk, while interactions with the more frequent CC genotype decrease the asthma risk (data not shown).

**Table 5:** Gene-gene (SNP-SNP) joint relevance and interactions for asthma susceptibility.

#	Genes	SNPs *	Posterior probability**	p-value***	exp(B) (95%CI)***
1	*PTGER2*	rs17197	0.81	0.134	1.51 (0.88–2.59)
	*PTGER2*	rs708502			
2	*FRMD6*	rs3751464	0.80	0.00377	0.55 (0.36–0.82)
	*PTGER2*	rs708502			
3	*PTGER2*	rs17197	0.79	0.012	1.68 (1.12–2.5)
	*FRMD6*	rs3751464			
4	*PRPF19*	rs7928208	0.61	0.00067	1.19 (1.08–1.32)
	*FRMD6*	rs3751464			
5	*PRPF19*	rs7928208	0.59	0.0049	0.2 (0.07–0.62)
	*PTGER2*	rs708502			
6	*PRPF19*	rs7928208	0.58	0.038	3.1 (1.06–9.05)
	*PTGER2*	rs17197			
7	*PTGER2*	rs17197	0.78	0.0166	0.62 (0.42–0.92)
	*FRMD6*	rs3751464			
	*PTGER2*	rs708502			
8	*PRPF19*	rs7928208	0.58	0.256	1.16 (0.89–1.5)
	*PTGER2*	rs17197			
	*PTGER2*	rs708502			
9	*PRPF19*	rs7928208	0.56	0.0012	0.71 (0.57–0.87)
	*FRMD6*	rs3751464			
	*PTGER2*	rs708502			
10	*PRPF19*	rs7928208	0.55	0.018	1.26 (1.04–1.53)
	*PTGER2*	rs17197			
	*FRMD6*	rs3751464			

* Interaction terms were forced into logistic regression using the enter method. The main effects entered are indicated with underscore.

** Posterior probability of joint relevance.

*** P-value and exp(B) values corresponding to the interaction terms in logistic regression using continuous variables.

#### Detailed characterization of association relations

**Figure 3:**
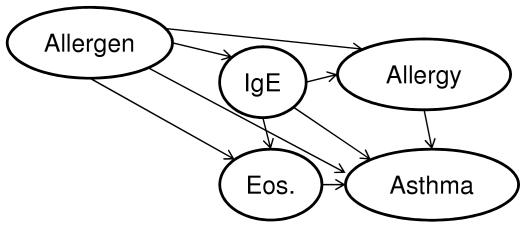
A possible dependency model in asthma. An example for a possible transitive association between IgE and Asthma. Although there is a possible direct relationship, IgE level may relate to asthma indirectly via eosinophil or a presence of allergy.

The association of a genetic variant to a phenotypic feature can have multiple types. First, because of the dependency of the genetic factors (due to linkage disequilibrium or evolutionary patterns), the markers typically found in genetic association studies are rarely directly associated to the phenotype. In these cases, the association is transitive, i.e. it is mediated by the causal SNP. For example, in case of *PTGDR*, the posterior of association of the SNPs (rs17831675, rs17831682, and rs803012) to asthma are larger than 0.9, but rs803012 can be excluded as strongly relevant, because its posterior of strong relevance is lower than 0.005, which indicates its non-causal, non-functional role. Second, in case of multiple targets, which originate potentially from a complex dependency model, the association can be mediated by phenotypic variables. For example in case of the asthma a possible dependency model is shown in Figure 3, in which a SNP affecting IgE might be both directly and transitively associated with asthma.

To evaluate the global characterization of association types shown in Table 2, we computed the a posteriori probability whether a variable is directly relevant or its association is only mediated in cases where the rhinitis status was known (RA dataset; Table 6). Additionally, we computed whether a variable is in pure interaction or its association is pure confounded.

**Table 6:** The a posteriori probability that a SNP is directly relevant (D-Relevant), associated, strongly relevant (S-Relevant), transitive or in pure interaction with asthma using the RA data set.

GENE	SNP	Associated	D-Relevant	S-Relevant	Interaction	Transitive
*AHNAK*	rs11231128	0.643	0.029	0.736	0.708	0.535
*AHNAK*	rs11827029	0.868	0.021	0.728	0.707	0.399
*FRMD6*	rs3751464	0.862	0.284	0.300	0.016	0.331
*MS4A2*	rs569108	0.653	0.087	0.111	0.024	0.633
*PRPF19*	rs7928208	0.878	0.718	0.843	0.125	0.822
*PTGDR*	rs17831675	0.923	0.326	0.362	0.035	0.747
*PTGDR*	rs17831682	0.923	0.524	0.578	0.000	0.863
*PTGDR*	rs803012	0.973	0.000	0.002	0.002	0.539
*PTGER2*	rs1254600	0.970	0.088	0.090	0.000	0.353
*PTGER2*	rs1254601	0.989	0.013	0.046	0.033	0.126
*PTGER2*	rs12587410	0.618	0.157	0.405	0.248	0.522
*PTGER2*	rs17197	0.970	0.350	0.354	0.004	0.604
*PTGER2*	rs708498	0.983	0.002	0.227	0.225	0.108
*TXNDC16*	rs1565970	0.309	0.008	0.722	0.713	0.189

Here we present some examples how the results in Table 6 can be interpreted. The posterior that rs7928208 (*PRPF19*) is transitively associated to asthma is 0.822, and the posterior for a “direct” relation, which is not blocked by any other variable, is similarly high (0.718; RA dataset, asthma as a target variable). On the contrary, the posterior that, rs569108 in *MS4A2* is transitively associated with asthma is 0.633, but the posterior for a “direct” relation is only 0.087. Another interesting example is rs11231128 in *AHNAK*. The posterior that it is transitively associated with asthma is 0.535, the posterior that it is strongly relevant is 0.736, and the posterior for a “direct” relation is only 0.029, The higher probability of strong relevance compared to the posterior for a transitive relation indicates a pure interaction (0.708), which suggests that this SNP is relevant only if the rhinitis status is known. Furthermore, when rhinitis status was excluded from the data set the strong relevance of rs11231128 for asthma vanished (data not shown). This shows that this SNP is strongly relevant through interaction with rhinitis.

#### Association to multiple targets

To decompose the relevance of genetic factors for various phenotypes we computed and compared the posteriors for the strongly relevant variables with respect to each target variable, namely IgE, and eosinophil levels, rhinitis and asthma (CLI dataset), which participate in a complex causal model with multiple paths. Posteriors of the decomposed relevance for multiple target variables are presented in Table 7.

**Table 7:** The posterior probability of strong relevance of predictors for each target and for a multi-target case based on the CLI data set.

		Exist					Only				Other than				
GENE	SNP	IgE	Eos	Rhi	Ast	AP	IgE	Eos	Rhi	Ast	IgE	Eos	Rhi	Ast	MT
*PTGER2*	rs1254600	0.08	0.12	0.15	0.31	0.52	0.04	0.07	0.08	0.21	0.44	0.40	0.38	0.21	0.46
*PTGER2*	rs12587410	0.31	0.38	0.53	0.81	0.96	0.02	0.02	0.04	0.16	0.65	0.58	0.43	0.15	0.91
*PTGER2*	rs17197	0.08	0.17	0.22	0.73	0.84	0.01	0.03	0.05	0.43	0.76	0.67	0.62	0.11	0.85
*PTGER2*	rs708498	0.09	0.04	0.05	0.61	0.68	0.03	0.01	0.02	0.50	0.59	0.64	0.62	0.07	0.67
*PTGDR*	rs17125273	0.17	0.13	0.15	0.10	0.45	0.12	0.08	0.10	0.06	0.27	0.32	0.30	0.35	0.31
*PTGDR*	rs17831675	0.52	0.41	0.48	0.44	0.92	0.09	0.06	0.08	0.06	0.40	0.51	0.44	0.48	0.66
*PTGDR*	rs17831682	0.59	0.53	0.53	0.53	0.96	0.06	0.05	0.05	0.05	0.37	0.43	0.42	0.43	0.71
*MS4A2*	rs569108	0.31	0.47	0.37	0.43	0.87	0.06	0.12	0.08	0.10	0.56	0.40	0.50	0.44	0.77

Target variables: IgE level - *IgE*, Eosinophil level – *Eos*, Rhinitis – *Rhi*, Asthma – *Ast*.

“*Exist*” denotes the probability of strong relevance with respect to a given target.

“*Only*” denotes posteriors for strong relevance to exactly one of the targets.

“*OtherThan*”denotes posteriors for strong relevance to any other target than the one specified by the subcolumn.

“*AP*” column contains an approximation of multi-target strong relevance based on the individual strong relevance posteriors of the targets.

“*MT*” denotes the posterior of multi-target strong relevance.

We would like to demonstrate how the results in Table 7 can be interpreted through an example. Following the earlier discussion of rs569108 in *MS4A2*, the results on the CLI data indicate that this SNP is strongly relevant to some of the CLI targets with a posterior of 0.77. In case of rs708498 in *PTGER2* the difference between the posteriors is more significant. The posteriors for strong relevance with respect to IgE, eosinophil level, rhinitis and asthma is 0.09, 0.04, 0.05,0.61, which clearly indicates that a relationship between rs708498 and asthma is more probable than a relationship with any other targets. Furthermore, the posterior probability that rs708498 is strongly relevant exclusively to IgE, eosinophil level, rhinitis or asthma are 0.03, 0.01,0.02, 0.50, which shows that it is more probable that this SNP is exclusively related to asthma than to any other phenotype. This is also supported by the posteriors that this SNP is strongly relevant to other targets, but not to IgE,eosinophil level, rhinitis or asthma: 0.58, 0.64, 0.62, and 0.07, with the lowest posterior for asthma in this respect. Finally, the posterior for rs708498 being a relevant SNP for multiple phenotypes (0.67) is relatively close to the posterior of strong relevance for asthma (0.61), which shows that relations to other targets are negligible.

**Figure 4:**
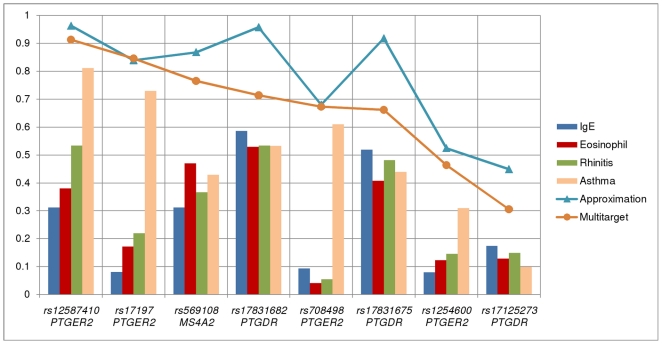
The posterior probability of strong relevance of predictors for each target and for a multi-target case based on the CLI data set. Posterior probabilities for strong relevance to Asthma, Rhinitis, Eosinophil and IgE level are indicated by different columns. Posteriors of joint strong relevance, i.e. multi-target relevance and its approximation based on the individual posteriors for strong relevance with respect to Asthma, Rhinitis, Eosinophil and IgE level are denoted by orange and blue curves, respectively. The approximation assumes independence between the targets, whereas the multi-target posterior accounts for the possible dependencies between the targets.

Figure 4 displays the posteriors for strong relevance detailed in Table 7 providing an overall view on strong relevance with respect to the targets in CLI data set on the level of genes. It indicates that *PTGER2* (rs12587410, rs17197, rs1254600, rs708498) is related to asthma with a higher probability, whereas *PTGDR* (rs17831682, rs17831675, rs17125273) is slightly more relevant with respect to IgE levels, though the difference between posteriors for strong relevance of different targets is lower in the latter case.

The fact that the average posterior for strong relevance in case of the most relevant SNPs is moderate (in case of asthma as a target) or low (in case of IgE, eosinophil and rhintis) indicates that the CLI data set is at its limit in terms of data sufficiency. In fact, this resembles the flat posterior case mentioned previously. However, even in this case, the results provided by the BN-BMLA method at least allow a restricted analysis of the domain. Furthermore, in case of multiple targets, the joint strong relevance (multi-target relevance) approach provides a more robust posterior.

Figure 4 also shows the relationship between the strong multi-target relevance and its approximation based on single target posteriors. The former accounts for the interdependencies between targets, while the approximation treats the targets as independent factors. Therefore, in domains that contain a complex dependency model, the multi-target approach is more viable, since the assumption of independency will not hold. Thus the approximation for a joint relevance based on independently treated posteriors of targets will yield inaccurate results.

### Analysis of gene expression in mice and men

Earlier we have carried out measurements of gene expression levels by Agilent Whole Mouse Genome Oligo Microarray 4×44 K chips in the lungs of mice with allergic airway inflammation and control mice (GSE11911 record number in GEO database) [35]. All of the genes found to be relevant in the present study were expressed in the lung of the mice. We compared the expression level of the genes in the lungs of mice with OVA-induced allergic airway inflammation and control mice. Altogether, 1134 transcripts showed >2.0-fold statistically significant differential expression [35], but none of the relevant genes in this study showed this level of difference. However, in all mice with OVA-induced experimental asthma the expression level of *FRMD6* was consequently lower (in average with 1.52 fold). From the relevant genes in the present study no other genes showed such a consequent correlation.

**Figure 5:**
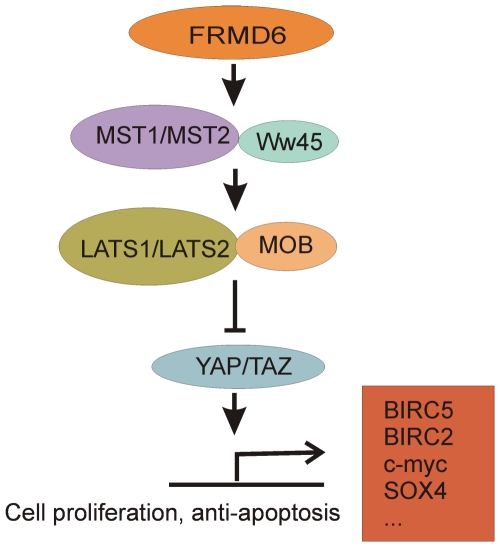
Hypothesized connection between FRMD6 and Birc5 in the conserved Hippo pathway. Hypothetic hippo pathway components in mammals are shown in various colors, with pointed and blunt arrowheads indicating activating and inhibitory interactions, respectively. The pathway regulates transcriptions of several genes, among others that of *Birc5*. Based on [36], [37], [38]. According to this pathway lower level of *FRMD6* might be associated with higher level of *Birc5*, as was found in the lung of the animal model of asthma.

Next, we studied the changes in the expression levels of genes in the known pathway involving *FRMD6*. The *FRMD6* is part of the conserved Hippo pathway playing a critical role in controlling organ size by regulating both cell proliferation and apoptosis [36] (Figure 5). One of the best known target genes of this pathway is the antiapoptotic *Birc5* [37], [38] (also known as survivin) whose expression level showed 5.94 fold increase (corrected P = 0.001) in the lung of OVA induced mice.

We compared the gene expression levels of genes found to be relevant in asthma in the SNP analysis in sputum samples of 12 asthmatics and 9 controls using TaqMan Gene Expression Assays (*FRMD6*, *PTGDR*, *PTGER2*, *MS4A2*, *AHNAK*, *PRPF19*, *TXNDC16*). Sputum mRNA level of *FRMD6* was significantly lower in the asthmatic patients compared to healthy controls with a fold change of 2.73 (p = 10^−6^). No other gene showed statistically significant difference in this comparison.

## Discussion

In this paper we presented the results of a partial genome screening in asthma evaluated by several statistical methods. In 11q12.2-q13.1 and 14q22.1-22.3 genome regions, which were earlier identified as asthma susceptibility regions, we successfully genotyped 102 SNPs in 57 genes. Earlier, in different association studies several asthma genes were identified in these regions, but none of them were confirmed later by GWAS, although two of them (*PTGDR* and *GSTP1*) were verified in candidate gene association studies in several independent populations [9]. Additionally, in a study where the results of GWAS were combined with a candidate gene approach, polymorphisms in *GSTP1* also showed an effect on asthma susceptibility [9]. In our present study, using the BN-BMLA method, several earlier results were confirmed. Associations were confirmed between SNPs in *PTGDR*, *PTGER2*, *MS4A2* and asthma. Interestingly, however, the frequentist method could only identify the association of the *PTGDR* gene, and was unable to detect it in the case of the other two genes. The explanation for this phenomenon is, that according to our evaluation, SNPs in the *PTGER2* influence asthma susceptibility in interactions or indirectly, and similarly, the association between a SNP in *MS4A2* and asthma is transitive, which is hard to detect with traditional frequentist methods. This might be one explanation why the association of the polymorphisms in this gene, which is otherwise a very plausible gene in asthma and atopic diseases, could not be confirmed in the majority of the studies using traditional statistical methods [39], [40], [41].

*MS4A2* gene (earlier known as FcεRI-β), which codes for the high affinity IgE receptor β subunit has a central role in mast cell degranulation and IgE mediated allergy.

The rs569108 SNP, which corresponds to the E237G amino acid substitution, is predicted to introduce a hydrophobicity change within the C-terminus of the receptor. It is adjacent to the immunoreceptor tyrosine activation motif, and may affect the intracellular signaling capacity of the receptor. The *MS4A2* was one of the first candidate genes in atopic diseases, and already in 1996 associations were found between E237G and significantly elevated skin test responses to different allergens and bronchial reactivity to methacholine in a UK population [42]. Since then, several studies in different populations have investigated the role of this polymorphism in asthma and atopy with very controversial results. In this study we could not confirm a direct association between E237G and asthma, but found a transitive association only when each target variable, namely IgE, and eosinophil levels, rhinitis and asthma (CLI dataset) were considered, which corresponded to a complex model with multiple paths.

It is well documented that, in asthmatics, prostaglandin D2 modulates the physiology of the airways by causing bronchoconstriction, vasodilation, and increase in capillary permeability and mucus production. Mice, lacking *PTGDR* fail to develop bronchial hyperresponsiveness upon ovalbumin challenge, suggesting that this receptor has an important role in the disease [43]. Polymorphisms in the gene have been reported to be associated with asthma in American, European and Japanese populations, but not in Chinese children, Latinos or Koreans [44]. In our study on Hungarian children, several SNPs showed different types of associations (direct or transitive). Interestingly, three SNPs (rs17831682, rs17831675, rs17125273) were slightly more relevant with respect to IgE levels, than asthma or other targets.

Prostaglandin E2 exerts anti-inflammatory and bronchoprotective mechanisms in asthma, it inhibits the chemotaxis of eosinophils toward eotaxin, prostaglandin D2 and C5a [45]. Polymorphisms in its receptor *PTGER2* were mainly found to be associated with aspirin-intolerant asthma, but in some studies also with asthma in general [46]. In our study the distribution of the SNPs in the *PTGER2* gene did not differ between the asthmatic and control groups. But, when SNP-SNP interactions were calculated, polymorphisms of the *PTGER2* participated in each significant association. This implies that these SNPs do not influence asthma risk alone, but in interaction with other SNPs. When multiple targets were considered the SNPs of this gene showed relevance only to asthma and the relations to other targets (IgE and eosinophil levels or rhinitis) were negligible.

Besides confirming previous results, the present study also detected new asthma genes in these regions. The most remarkable result of the study is the role of *FRMD6* in asthma. Association between a SNP in *FRMD6* and asthma risk was identified with both the frequentist and BN-BMLA methods. A haplotype in this gene also influenced the disease susceptibility, and the rs3751464 showed an influential role in interactions with other SNPs in this respect. Additionally, the expression level of the *FRMD6* was consistently lower in the lungs of mice with allergic airway inflammation and it was significantly lower in human asthmatics compared with controls. The exact function of this gene and its possible role in asthma are unknown, but some theories can be generated from earlier and our present data. *FRMD6* (FERM domain containing 6, earlier also EX1, or Willin) is suspected to be an upstream component of the Hippo signaling pathway [36]. Several recent publications establish that the pathway is one major conserved mechanism governing cell contact inhibition and organ size control [36], [37]. Clearly, even small changes in this pathway can alter the lung morphogenesis which can lead to modified response to environmental challenges and susceptibility to asthma. The role of this pathway in asthma is also supported by our findings that the expression of the antiapoptotic *Birc5* (also known as survivin), one of the best known target genes of this pathway was drastically reduced in the lung of mice with airway inflammation. This finding supports the theory that one of the mechanisms which can play a role in asthma is the reduced apoptotic potential of the airway epithelium. Studies supporting this theory showed that after rhinovirus infections, the epithelial cells of the asthmatic lungs were unable to enter into apoptosis with the consequence that the replicating virus caused cytopathic cell death with extensive virus shedding [47], [48]. It can be asked, however, why earlier GWAS were unable to detect *FRMD6*? The simplest explanation is that in these studies only one SNP in the *FRMD6* gene was genotyped (rs7140150), which is not in LD with rs3751464. This supports the observation of Michel et al that GWAS coverage is insufficient for many asthma candidate genes [9]. In addition, these GWASs were carried out in other populations, which could significantly influence the results.

The rs3751464 SNP localizes in the 5′ untranslated region of the *FRMD6* gene on chromosome 14q22.1 at 52117892 base pair. With *in silico* methods, we were unable to find out the role of this SNP (or SNPs in LD with rs3751464) in the expression or function of *FRMD6*, or in the disease. In public databases no data were available whether this SNP or its haplotype influence the binding of any regulator elements.

Another interesting and novel finding of this study is the indirect but strong relevance of the SNPs in the *AHNAK* gene to asthma. The SNPs in this gene influenced asthma risk in interaction with rhinitis. *AHNAK* is a ubiquitous protein expressed in a variety of cell types. In epithelial cells *AHNAK* is distributed mainly on the cell membranes, suggesting its role in the formation of tight junction. Now, it is widely accepted that impaired formation of tight junction leads to reduced barrier function and increased susceptibility to asthma [49], [50], [51]. Notably, the rs11231128 is a missense SNP, causing a serine proline amino acid substitution at position 3724, which might influence the 3D structure of the protein. In addition, according to the Ingenuity database, *AHNAK* interacts with several factors playing important role in the disease, like *TGFB1*, *EGFR*, *IL6* and *STAT4* (Ingenuity Pathway Analysis (IPA) 9.0 Software (Ingenuity Systems, Redwood City, CA, USA; www.ingenuity.com)).

Similarly to the SNPs in *AHNAK*, a SNP in the *TXNDC16* gene also influenced asthma risk in interaction with rhinitis. Until now, however, there has been no information about the function of this gene in any databases.

Strong and direct association was found between rs7928208 in the *PRPF19* gene and asthma and this SNP was also associated with asthma development before 6 years of age. Although its possible role in asthma is unknown, the product of the gene, similarly of some earlier found asthma genes (e.g. *RAD50*) plays a role in DNA repair, thus theoretically its altered function might influence the resistance of the cells to environmental stress [52], [53], [54].

The above discussed results clearly show the several advantageous features of the BN-BMLA method over the traditional frequentist methods generally used in gene association studies. As can be seen from the results, the advantage is not only that the BN-BMLA can detect more relevant variables, but the Bayesian networks offer a rich language for the detailed representation of types of relevance, including causal, acausal, and multitarget aspects. Additionally, Bayesian statistics offers an automated and normative solution for the multiple hypothesis testing problem. The computational complexity is manageable using high-throughput and high-performance computing resources for medium sized problems with hundreds of variables. This extends the scope of local ‘causal’ discovery methods, and because of the direct interpretation of Bayesian posteriors, contrary to p-values from the frequentist approach, makes it an ideal candidate for creating probabilistic knowledge bases to support off-line meta-analysis and fusion of background knowledge.

We analyzed partial multivariate strong relevances, because the Bayesian statistical framework allows the calculations of posteriors over a wide range of hypotheses, such as strong relevance of variables, pairs of variables, triplets of variables, etc. This shows the advantage of the Bayesian framework, because it allows the selection of appropriate level of complexity of hypotheses, which is not possible in the traditional hypothesis testing approach.

### Conclusion

In a partial genome screening of asthma we identified *FRMD6* as a novel asthma gene. The possible role of this gene was also confirmed in an animal model and in human asthmatics. Beside *FRMD6*, using BN-BMLA method, we identified several additional genes (*PTGDR*, *PTGER2*, *MS4A2*, *AHNAK*, *PRPF19*, *TXNDC16*), which directly, or indirectly might play a role in the disease. In contrast to BN-BMLA, the traditional and novel frequentist based methods (χ^2^ test, multivariate logistic regression and multifactor dimensionality reduction) could consistently identify only the direct effect of *FRMD6* on asthma risk, and in some models the possible effect of *PTGDR*, *PRPF19* and *PTGER2*.

The BN-BMLA on one hand extends the scope of strong relevance based methods towards (1) partial multivariate relevance, (2) global characterization of pairwise relevances, and (3) multi-target relevances. On the other hand it can be seen as focusing the general, global feature learning techniques towards relevance analysis, i.e. from learning arbitrary dependency substructures to learning strongly relevant sets. Furthermore, this Bayesian global relevance analysis method provides posteriors, which are direct statements about hypotheses, thus it can also be used to construct probabilistic data analytic knowledge bases in genetic association studies to support complex quering, off-line meta-analysis, and fusion with background knowledge. Although, in artificial datasets we have previously demonstrated the superior ability of the BN-BMLA method to detect real factors and interactions in genetic association studies, the method must be applied to other real word datasets, and the results must be validated in alternative methods. If these studies confirm the usefulness of this new statistical method, it could be a good alternative to evaluate the results of gene or genome association studies.

The tool is accessible at http://webbmla.genagrid.eu.

## Supporting Information

Figure S1 **Comparison of standard concept of (pairwise) association and strong relevance.** The concept of strong relevance and association (with respect to a target) has only one common element, direct relevance, i.e. non-mediated relationship between the target and a variable. Association also includes confounded and transitive relevance, where there is a mediator between the given variable and the target. In cause-effect relationship terms, the confounded case corresponds to a common cause; the transitive case corresponds to a cause- effect path. Strong relevance, on the other hand, includes interactionist relevance, i.e. a common effect type relationship.
(TIF)

Figure S2 **Haplotype blocks of the investigated SNPs in the present study.** SNPs are numbered sequentially and their relative location is indicated along the top. Markers 1–54 and markers 55–102 correspond to the studied SNPs on Chromosome 14 and 11, respectively. Triangles surrounding markers represent haplotype blocks.
(TIF)

Figure S3 **The peakness of the posteriors of the most probable 100 MBS sets.** The x axis denotes the rank of a Markov blanket set (MBS) of SNPs, the y axis denotes the joint probability of an MBS, e.g. an MBS with rank = 5 is the fifth most probable set. The “RA” and “CLI” prefixes denote the corresponding dataset, and the “asthma” and “multitarget” suffixes indicate the target variables. RA- multitarget: Rhinitis, Asthma; CLI- multitarget: Asthma, Rhinitis, Eosinophil and IgE level. Note, that the peakness of the posteriors decreases within the same dataset in the multitarget case; and between different data sets, the smaller sample size (CLI:200, RA:1100) results in weaker posteriors. In terms of data sufficiency, the flatness of the CLI MBS posterior curve (both in the single target and the multitarget case) indicates that the CLI data is not sufficient for a complete multivariate analysis. The RA dataset, on the other hand, is more appropriate having a relatively peaked posterior, although the maximum posterior is not particularly high.
(TIF)

Figure S4 **The most probable univariate (MBM), bivariate (2-MBS), trivariate (3-MBS), 4-MBS, 5-MBS subsets of variables.** All posteriors of partial k-relevance are ordered, *x* axis denotes the rank (e.g.: the number 2 means the second highest posterior), and *y* axis denotes the posterior probabilities. As *k* increases (i.e. the set size of jointly considered SNPs) the maximum posteriors decrease, and the slope of the posteriors are much less “peaked”, which means that the lower ranked posteriors are significantly higher than those with higher ranks. The univariate case can be considered as a relatively peaked curve, and as *k* increases, the curve “flattens”. The curve of whole sets (MBS) is the “flattest”, showing only a small difference between the lower ranked and the higher ranked posteriors. In this case, estimating the top 20 MBS is less informative than estimating the top 10 partial k-relevance of k = 2. This indicates the viability of the concept of partial k-relevance.
(TIF)

Table S1 **Information on the examined SNPs.**
(XLS)

Table S2 **Minor allele and genotype frequencies (%) in asthmatic (n = 436) and control (n = 765) patients.**
(DOC)

Table S3 **Statistical evaluation for association of SNPs with asthma at allele and genotype levels.**
(DOC)

## References

[1] Daniels SE, Bhattacharrya S, James A, Leaves NI, Young A (1996). A genome-wide search for quantitative trait loci underlying asthma.. Nature.

[2] Ku MS, Sun HL, Lu KH, Sheu JN, Lee HS (2011). The CC16 A38G polymorphism is associated with the development of asthma in children with allergic rhinitis.. Clin Exp Allergy.

[3] Kamada F, Mashimo Y, Inoue H, Shao C, Hirota T (2007). The GSTP1 gene is a susceptibility gene for childhood asthma and the GSTM1 gene is a modifier of the GSTP1 gene.. Int Arch Allergy Immunol.

[4] Huang JL, Gao PS, Mathias RA, Yao TC, Chen LC (2004). Sequence variants of the gene encoding chemoattractant receptor expressed on Th2 cells (CRTH2) are associated with asthma and differentially influence mRNA stability.. Hum Mol Genet.

[5] (1997). A genome-wide search for asthma susceptibility loci in ethnically diverse populations. The Collaborative Study on the Genetics of Asthma (CSGA).. Nat Genet.

[6] Oguma T, Palmer LJ, Birben E, Sonna LA, Asano K (2004). Role of prostanoid DP receptor variants in susceptibility to asthma.. N Engl J Med.

[7] Ge XN, Bahaie NS, Kang BN, Hosseinkhani MR, Ha SG (2010). Allergen-induced airway remodeling is impaired in galectin-3-deficient mice.. J Immunol.

[8] Park HW, Shin ES, Lee JE, Kim SH, Kim SS (2007). Association between genetic variations in prostaglandin E2 receptor subtype EP3 gene (Ptger3) and asthma in the Korean population.. Clin Exp Allergy.

[9] Michel S, Liang L, Depner M, Klopp N, Ruether A (2010). Unifying candidate gene and GWAS Approaches in Asthma.. PLoS One.

[10] Ricci G, Astolfi A, Remondini D, Cipriani F, Formica S (2011). Pooled genome-wide analysis to identify novel risk loci for pediatric allergic asthma.. PLoS One.

[11] Denham S, Koppelman GH, Blakey J, Wjst M, Ferreira MA (2008). Meta-analysis of genome-wide linkage studies of asthma and related traits.. Respir Res.

[12] Moore JH, Asselbergs FW, Williams SM (2010). Bioinformatics challenges for genome-wide association studies.. Bioinformatics.

[13] Aliferis CF, Statnikov A, Tsamardinos I, Mani S, Koutsoukos XD (2010). Local Causal and Markov Blanket Induction for Causal Discovery and Feature Selection for Classification.. Journal of Machine Learning Research.

[14] Friedman N, Goldszmidt M, Wyner A (1999). On the application of the Bootstrap for computing confidence measures on features of induced Bayesian networks.. Artificial Intelligence and Statistics 99, Proceedings.

[15] Pe'er D, Regev A, Elidan G, Friedman N (2001). Inferring subnetworks from perturbed expression profiles.. Bioinformatics.

[16] Xing H, McDonagh PD, Bienkowska J, Cashorali T, Runge K (2011). Causal Modeling Using Network Ensemble Simulations of Genetic and Gene Expression Data Predicts Genes Involved in Rheumatoid Arthritis.. Plos Computational Biology.

[17] Stephens M, Balding DJ (2009). Bayesian statistical methods for genetic association studies.. Nat Rev Genet.

[18] Antal P, Hullam G, Gezsi A, Millinghoffer A (2006). Learning complex bayesian network features for classification. Proc of third European Workshop on Probabilistic Graphical Models.

[19] Antal P, Millinghoffer A, Hullam G, Szalai C, Falus A (2008). A Bayesian View of Challenges in Feature Selection: Feature Aggregation, Multiple Targets, Redundancy and Interaction..

[20] Antal P, Millinghoffer A, Hullam G, Hajos G, Szalai C, Flower DR, Davies MN, Ranganathan S (2010). A bioinformatic platform for a Bayesian, multiphased, multilevel analysis in immunogenomics.. Bioinformatics for Immunomics, Immunomics reviews.

[21] Hullam G, Antal P, Szalai C, Falus A, Sašo Džeroski PG, Juho Rousu editor (2010). Evaluation of a Bayesian model-based approach in GA studies.. JMLR Workshop and Conference Proceedings Volume 8: Machine Learning in Systems Biology, Proceedings of the third International Workshop on Machine Learning in Systems Biology.

[22] Purcell S, Cherny SS, Sham PC (2003). Genetic Power Calculator: design of linkage and association genetic mapping studies of complex traits.. Bioinformatics.

[23] Moore JH, Gilbert JC, Tsai CT, Chiang FT, Holden T (2006). A flexible computational framework for detecting, characterizing, and interpreting statistical patterns of epistasis in genetic studies of human disease susceptibility.. J Theor Biol.

[24] Pearl J (1988). Probabilistic reasoning in intelligent systems: networks of plausible inference.

[25] John GH, Kohavi R, Pfleger K (1994). Irrelevant features and the subset selection problem. Machine Learning: Proceedings of the Eleventh International Conference (ICML '94).

[26] Friedman N, Goldszmidt M, Wyner A (1999). Data analysis with bayesian networks: A Bootstrap approach..

[27] Tsamardinos I, Aliferis CF (2003). Towards principled feature selection: Relevancy, filters, and wrappers..

[28] Friedman N, Koller D (2003). Being Bayesian about network structure. A Bayesian approach to structure discovery in Bayesian networks.. Machine Learning.

[29] Cooper GF, Herskovits E (1992). A Bayesian Method for the Induction of Probabilistic Networks from Data.. Machine Learning.

[30] Glymour C, Cooper GF (1999).

[31] Liu JS (2001). Monte Carlo strategies in scientific computing.

[32] Gelman A, Carlin JB, Stern HS, Rubin DB (1995). Bayesian Data Analysis.

[33] Madigan D, Andersson SA, Perlman MD, Volinsky CT (1996). Bayesian model averaging and model selection for Markov equivalence classes of acyclic digraphs.. Communications in Statistics-Theory and Methods.

[34] Giudici P, Castelo R (2003). Improving Markov Chain Monte Carlo model search for data mining.. Machine Learning.

[35] Tolgyesi G, Molnar V, Semsei AF, Kiszel P, Ungvari I (2009). Gene expression profiling of experimental asthma reveals a possible role of paraoxonase-1 in the disease.. Int Immunol.

[36] Zhao B, Li L, Lei Q, Guan KL (2010). The Hippo-YAP pathway in organ size control and tumorigenesis: an updated version.. Genes Dev.

[37] Heallen T, Zhang M, Wang J, Bonilla-Claudio M, Klysik E (2011). Hippo pathway inhibits Wnt signaling to restrain cardiomyocyte proliferation and heart size.. Science.

[38] Dong J, Feldmann G, Huang J, Wu S, Zhang N (2007). Elucidation of a universal size-control mechanism in Drosophila and mammals.. Cell.

[39] Simon Thomas N, Wilkinson J, Lonjou C, Morton NE, Holgate ST (2000). Linkage analysis of markers on chromosome 11q13 with asthma and atopy in a United Kingdom population.. Am J Respir Crit Care Med.

[40] Li H, Xiaoyan D, Quanhua L, Jie L, Yixiao B (2009). Single-nucleotide polymorphisms in genes predisposing to asthma in children of Chinese Han nationality.. J Investig Allergol Clin Immunol.

[41] Zhu S, Chan-Yeung M, Becker AB, Dimich-Ward H, Ferguson AC (2000). Polymorphisms of the IL-4, TNF-alpha, and Fcepsilon RIbeta genes and the risk of allergic disorders in at-risk infants.. Am J Respir Crit Care Med.

[42] Hill MR, Cookson WO (1996). A new variant of the beta subunit of the high-affinity receptor for immunoglobulin E (Fc epsilon RI-beta E237G): associations with measures of atopy and bronchial hyper-responsiveness.. Hum Mol Genet.

[43] Honda K, Arima M, Cheng G, Taki S, Hirata H (2003). Prostaglandin D2 reinforces Th2 type inflammatory responses of airways to low-dose antigen through bronchial expression of macrophage-derived chemokine.. J Exp Med.

[44] Leung TF, Li CY, Kong AP, Chan IH, Ng MC (2009). PTGDR is not a major candidate gene for asthma and atopy in Chinese children.. Pediatr Allergy Immunol.

[45] Sturm EM, Schratl P, Schuligoi R, Konya V, Sturm GJ (2008). Prostaglandin E2 inhibits eosinophil trafficking through E-prostanoid 2 receptors.. J Immunol.

[46] Park BL, Park SM, Park JS, Uh ST, Choi JS (2010). Association of PTGER gene family polymorphisms with aspirin intolerant asthma in Korean asthmatics.. BMB Rep.

[47] Wark PA, Johnston SL, Bucchieri F, Powell R, Puddicombe S (2005). Asthmatic bronchial epithelial cells have a deficient innate immune response to infection with rhinovirus.. J Exp Med.

[48] Holgate ST, Davies DE (2009). Rethinking the pathogenesis of asthma.. Immunity.

[49] Holgate ST, Roberts G, Arshad HS, Howarth PH, Davies DE (2009). The role of the airway epithelium and its interaction with environmental factors in asthma pathogenesis.. Proc Am Thorac Soc.

[50] Kim IY, Jung J, Jang M, Ahn YG, Shin JH (2010). 1H NMR-based metabolomic study on resistance to diet-induced obesity in AHNAK knock-out mice.. Biochem Biophys Res Commun.

[51] de Boer WI, Sharma HS, Baelemans SM, Hoogsteden HC, Lambrecht BN (2008). Altered expression of epithelial junctional proteins in atopic asthma: possible role in inflammation.. Can J Physiol Pharmacol.

[52] Li X, Howard TD, Zheng SL, Haselkorn T, Peters SP (2010). Genome-wide association study of asthma identifies RAD50-IL13 and HLA-DR/DQ regions.. J Allergy Clin Immunol.

[53] Legerski RJ (2009). The Pso4 complex splices into the DNA damage response.. Cell Cycle.

[54] Voglauer R, Chang MW, Dampier B, Wieser M, Baumann K (2006). SNEV overexpression extends the life span of human endothelial cells.. Exp Cell Res.

