# Supplementary figures and images for: Evaluation of a Partial Genome Screening of Two Asthma Susceptibility Regions Using Bayesian Network Based Bayesian Multilevel Analysis of Relevance
Source: PLoS One. 2012 Mar 14;7(3):e33573. doi: 10.1371/journal.pone.0033573 (PMC3303848; doi:10.1371/journal.pone.0033573)

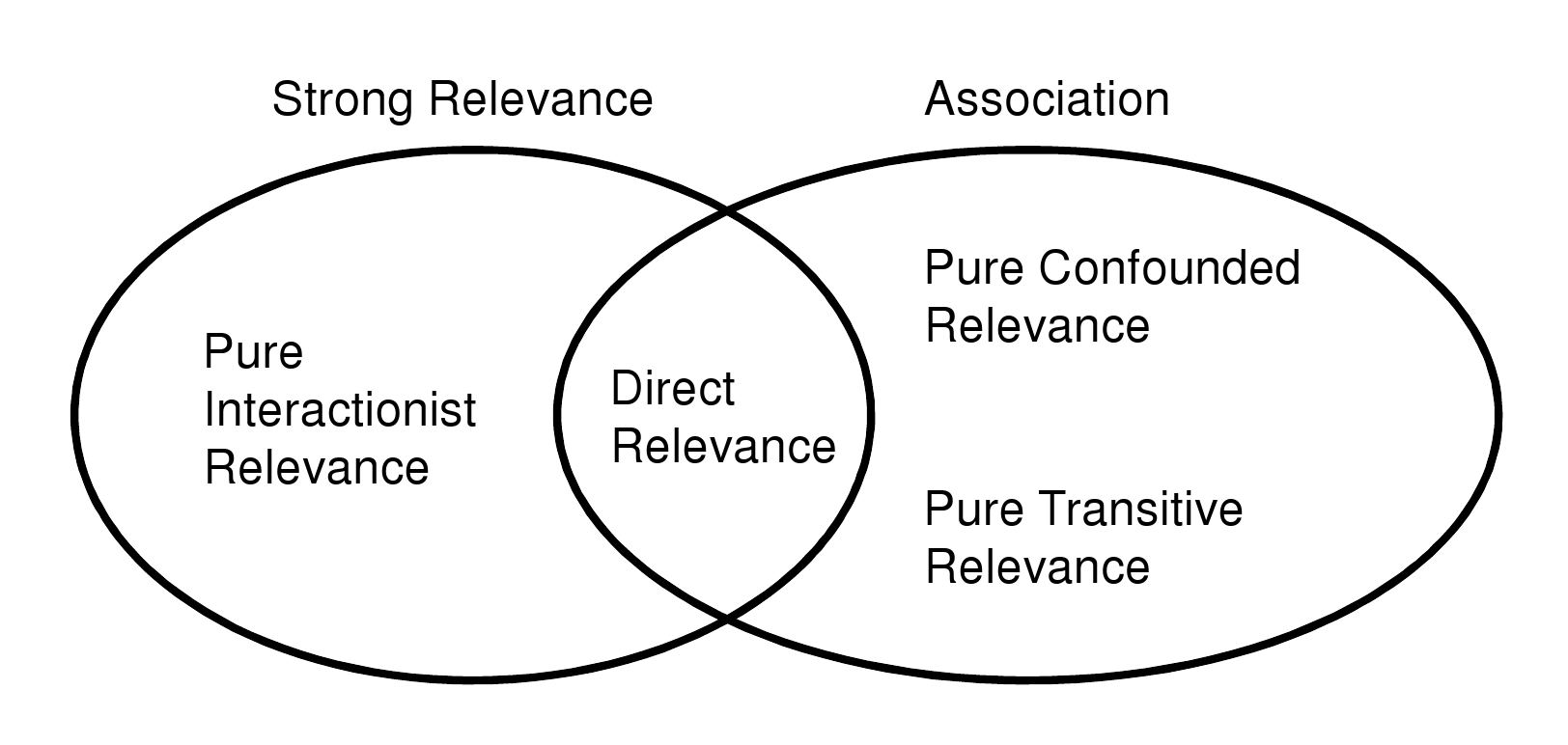

Supplement: Figure S1 — Comparison of standard concept of (pairwise) association and strong relevance. The concept of strong relevance and association (with respect to a target) has only one common element, direct relevance, i.e. non-mediated relationship between the target and a variable. Association also includes confounded and transitive relevance, where there is a mediator between the given variable and the target. In cause-effect relationship terms, the confounded case corresponds to a common cause; the transitive case corresponds to a cause- effect path. Strong relevance, on the other hand, includes interactionist relevance, i.e. a common effect type relationship. (TIF) [file pone.0033573.s001.tif]

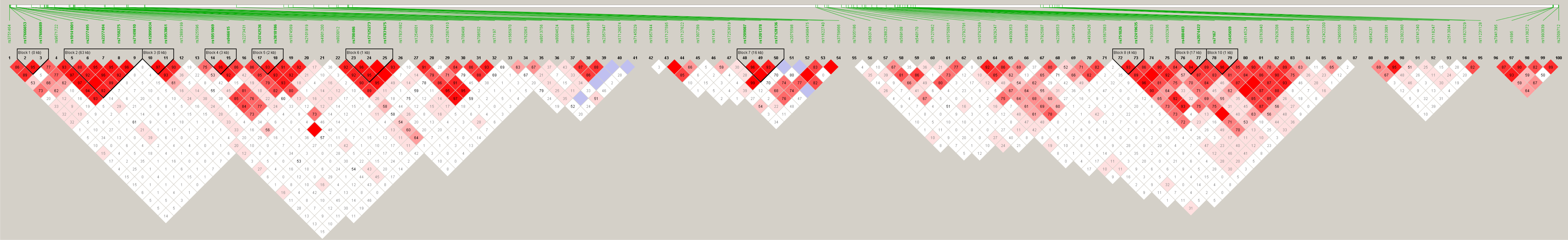

Supplement: Figure S2 — Haplotype blocks of the investigated SNPs in the present study. SNPs are numbered sequentially and their relative location is indicated along the top. Markers 1–54 and markers 55–102 correspond to the studied SNPs on Chromosome 14 and 11, respectively. Triangles surrounding markers represent haplotype blocks. (TIF) [file pone.0033573.s002.tif]

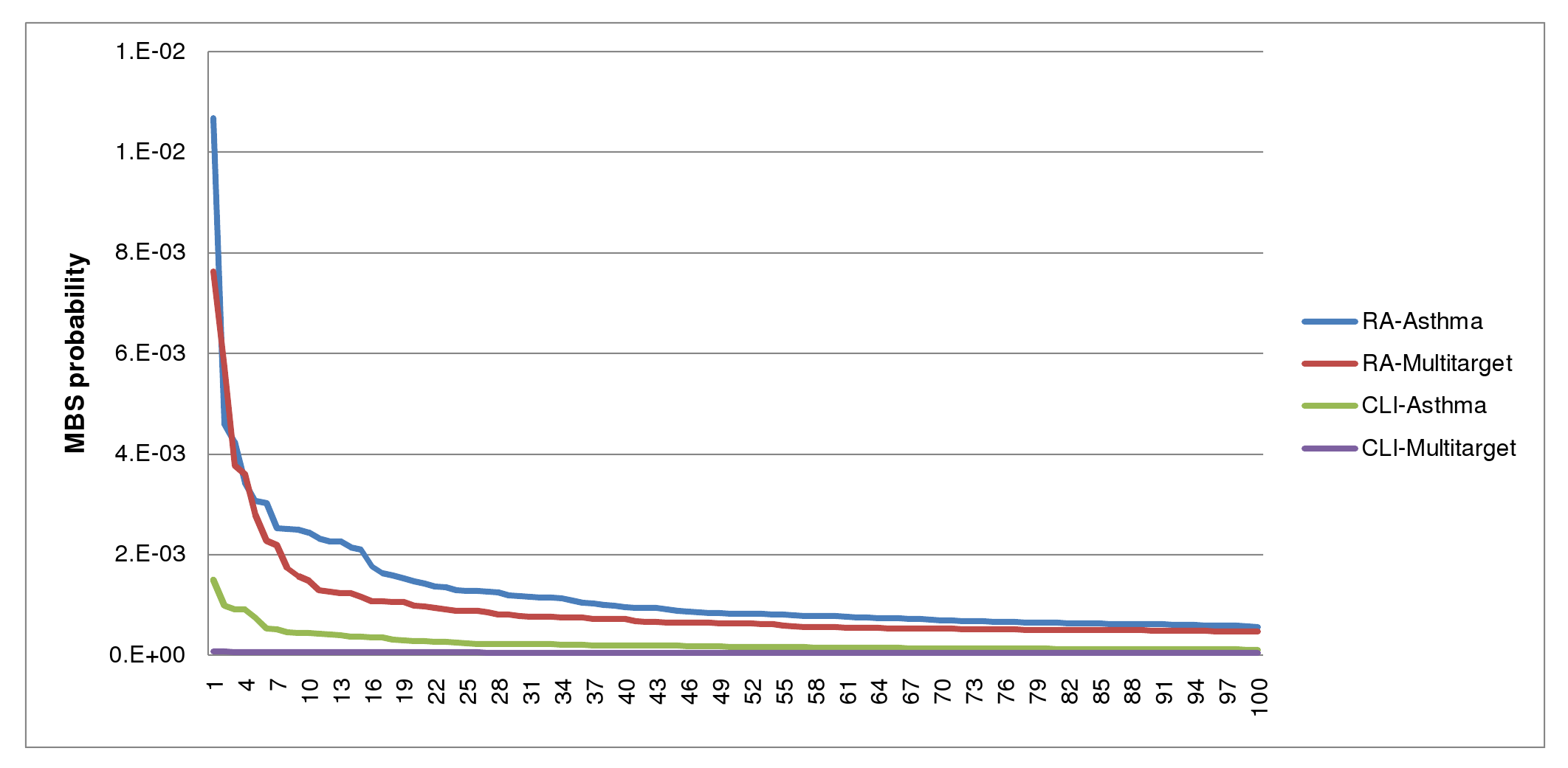

Supplement: Figure S3 — The peakness of the posteriors of the most probable 100 MBS sets. The x axis denotes the rank of a Markov blanket set (MBS) of SNPs, the y axis denotes the joint probability of an MBS, e.g. an MBS with rank = 5 is the fifth most probable set. The “RA” and “CLI” prefixes denote the corresponding dataset, and the “asthma” and “multitarget” suffixes indicate the target variables. RA- multitarget: Rhinitis, Asthma; CLI- multitarget: Asthma, Rhinitis, Eosinophil and IgE level. Note, that the peakness of the posteriors decreases within the same dataset in the multitarget case; and between different data sets, the smaller sample size (CLI:200, RA:1100) results in weaker posteriors. In terms of data sufficiency, the flatness of the CLI MBS posterior curve (both in the single target and the multitarget case) indicates that the CLI data is not sufficient for a complete multivariate analysis. The RA dataset, on the other hand, is more appropriate having a relatively peaked posterior, although the maximum posterior is not particularly high. (TIF) [file pone.0033573.s003.tif]

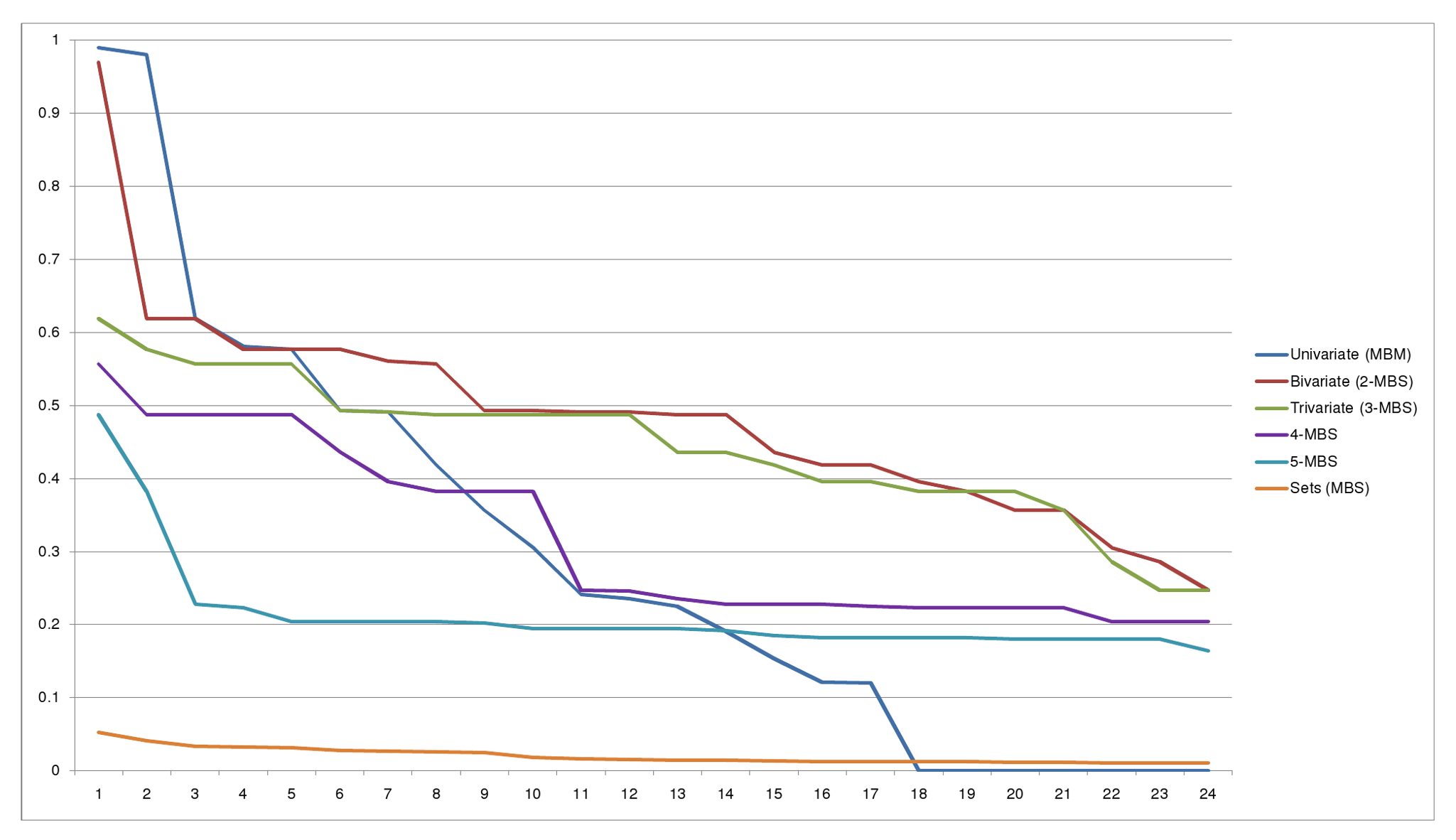

Supplement: Figure S4 — The most probable univariate (MBM), bivariate (2-MBS), trivariate (3-MBS), 4-MBS, 5-MBS subsets of variables. All posteriors of partial k-relevance are ordered, x axis denotes the rank (e.g.: the number 2 means the second highest posterior), and y axis denotes the posterior probabilities. As k increases (i.e. the set size of jointly considered SNPs) the maximum posteriors decrease, and the slope of the posteriors are much less “peaked”, which means that the lower ranked posteriors are significantly higher than those with higher ranks. The univariate case can be considered as a relatively peaked curve, and as k increases, the curve “flattens”. The curve of whole sets (MBS) is the “flattest”, showing only a small difference between the lower ranked and the higher ranked posteriors. In this case, estimating the top 20 MBS is less informative than estimating the top 10 partial k-relevance of k = 2. This indicates the viability of the concept of partial k-relevance. (TIF) [file pone.0033573.s004.tif]
