# Supplementary material for: Evaluation of a Partial Genome Screening of Two Asthma Susceptibility Regions Using Bayesian Network Based Bayesian Multilevel Analysis of Relevance
Source: PLoS One. 2012 Mar 14;7(3):e33573. doi: 10.1371/journal.pone.0033573 (PMC3303848; doi:10.1371/journal.pone.0033573)
Supplement: Table S2 — Minor allele and genotype frequencies (%) in asthmatic (n = 436) and control (n = 765) patients. (DOC) [file pone.0033573.s006.doc]

**Table S2 Minor allele and genotype frequencies (%) in asthmatic (n = 436) and control (n = 765) patients.**

| SNP | Control MAF | Case MAF | Genotype control* | | | Genotype cases* | | |
| --- | --- | --- | --- | --- | --- | --- | --- | --- |
|  |  |  | 0 | 1 | 2 | 0 | 1 | 2 |
| rs2513081 | 19.35 | 19.38 | 65.10 | 31.11 | 3.79 | 64.91 | 31.42 | 3.67 |
| rs7118247 | 19.74 | 16.97 | 65.23 | 30.07 | 4.71 | 69.27 | 27.52 | 3.21 |
| rs10750931 | 15.10 | 16.17 | 71.90 | 26.01 | 2.09 | 69.95 | 27.75 | 2.29 |
| rs528823 | 32.68 | 30.73 | 45.36 | 43.92 | 10.72 | 48.85 | 40.83 | 10.32 |
| rs2302360 | 21.24 | 20.87 | 63.27 | 30.98 | 5.75 | 62.61 | 33.03 | 4.36 |
| rs540170 | 45.75 | 48.97 | 29.02 | 50.46 | 20.52 | 24.54 | 52.98 | 22.48 |
| rs7127662 | 22.16 | 19.15 | 60.92 | 33.86 | 5.23 | 65.60 | 30.50 | 3.90 |
| rs2847204 | 13.07 | 11.12 | 76.21 | 21.44 | 2.35 | 78.90 | 19.95 | 1.15 |
| rs1567083 | 14.51 | 12.50 | 73.33 | 24.31 | 2.35 | 76.61 | 21.79 | 1.61 |
| rs7925087 | 7.52 | 5.96 | 85.36 | 14.25 | 0.39 | 88.30 | 11.47 | 0.23 |
| rs2074422 | 19.61 | 21.22 | 64.31 | 32.16 | 3.53 | 61.70 | 34.17 | 4.13 |
| rs488483 | 20.26 | 19.50 | 62.88 | 33.73 | 3.40 | 65.14 | 30.73 | 4.13 |
| rs7935803 | 35.75 | 39.91 | 42.61 | 43.27 | 14.12 | 38.76 | 42.66 | 18.58 |
| rs12422200 | 23.07 | 24.20 | 58.95 | 35.95 | 5.10 | 57.80 | 36.01 | 6.19 |
| rs7928208 | 2.03 | 3.56 | 96.21 | 3.53 | 0.26 | 92.89 | 7.11 | 0.00 |
| rs2237997 | 36.14 | 33.37 | 40.26 | 47.19 | 12.55 | 43.35 | 46.56 | 10.09 |
| rs3741240 | 35.23 | 35.21 | 43.01 | 43.53 | 13.46 | 40.60 | 48.39 | 11.01 |
| rs11827029 | 4.38 | 2.87 | 91.90 | 7.45 | 0.65 | 94.27 | 5.73 | 0.00 |
| rs11231128 | 3.07 | 2.06 | 93.99 | 5.88 | 0.13 | 95.87 | 4.13 | 0.00 |
| rs954237 | 37.25 | 37.50 | 40.13 | 45.23 | 14.64 | 39.22 | 46.56 | 14.22 |
| rs2905506 | 23.01 | 24.20 | 60.26 | 33.46 | 6.27 | 58.26 | 35.09 | 6.65 |
| rs563748 | 10.92 | 9.40 | 79.61 | 18.95 | 1.44 | 82.11 | 16.97 | 0.92 |
| rs10792269 | 36.60 | 34.52 | 40.52 | 45.75 | 13.73 | 44.50 | 41.97 | 13.53 |
| rs12792791 | 30.39 | 28.33 | 47.71 | 43.79 | 8.50 | 51.61 | 40.14 | 8.26 |
| rs7935186 | 24.25 | 26.49 | 59.08 | 33.33 | 7.58 | 53.90 | 39.22 | 6.88 |
| rs2513044 | 37.52 | 36.81 | 36.60 | 51.76 | 11.63 | 38.53 | 49.31 | 12.16 |
| rs2298553 | 48.63 | 48.51 | 25.62 | 51.50 | 22.88 | 27.98 | 47.02 | 25.00 |
| rs569108 | 3.01 | 2.18 | 94.51 | 4.97 | 0.52 | 0.23 | 3.90 | 95.87 |
| rs514524 | 34.05 | 31.31 | 43.27 | 45.36 | 11.37 | 47.02 | 43.35 | 9.63 |
| rs4939426 | 12.35 | 10.67 | 77.78 | 19.74 | 2.48 | 79.82 | 19.04 | 1.15 |
| rs1941030 | 37.84 | 36.35 | 38.43 | 47.45 | 14.12 | 39.45 | 48.39 | 12.16 |
| rs4939353 | 28.10 | 27.18 | 52.81 | 38.17 | 9.02 | 53.67 | 38.30 | 8.03 |
| rs3829247 | 27.39 | 26.49 | 52.94 | 39.35 | 7.71 | 53.67 | 39.68 | 6.65 |
| rs708498 | 17.58 | 18.00 | 67.71 | 29.41 | 2.88 | 66.51 | 30.96 | 2.52 |
| rs3818186 | 26.08 | 25.92 | 53.59 | 40.65 | 5.75 | 55.28 | 37.61 | 7.11 |
| rs3759666 | 1.18 | 1.26 | 97.65 | 2.35 | 0.00 | 97.48 | 2.52 | 0.00 |
| rs751026 | 46.80 | 49.54 | 28.50 | 49.41 | 22.09 | 27.75 | 45.41 | 26.83 |
| rs2101919 | 25.62 | 25.00 | 54.38 | 40.00 | 5.62 | 6.65 | 36.70 | 56.65 |
| rs17197 | 14.18 | 13.65 | 73.33 | 24.97 | 1.70 | 73.85 | 25.00 | 1.15 |
| rs2273431 | 8.17 | 9.52 | 84.31 | 15.03 | 0.65 | 82.80 | 15.37 | 1.83 |
| rs1307289 | 16.27 | 14.68 | 70.59 | 26.27 | 3.14 | 72.94 | 24.77 | 2.29 |
| rs1138272 | 9.35 | 7.45 | 82.35 | 16.60 | 1.05 | 86.24 | 12.61 | 1.15 |
| rs1254600 | 16.54 | 17.55 | 69.80 | 27.32 | 2.88 | 67.43 | 30.05 | 2.52 |
| rs12419635 | 9.22 | 9.86 | 82.22 | 17.12 | 0.65 | 80.96 | 18.35 | 0.69 |
| rs7941395 | 37.25 | 38.19 | 38.82 | 47.84 | 13.33 | 38.99 | 45.64 | 15.37 |
| rs2277494 | 24.05 | 23.97 | 58.69 | 34.51 | 6.80 | 58.94 | 34.17 | 6.88 |
| rs1051069 | 46.80 | 48.05 | 27.32 | 51.76 | 20.92 | 25.69 | 52.52 | 21.79 |
| rs3763840 | 34.64 | 35.55 | 42.88 | 44.97 | 12.16 | 41.74 | 45.41 | 12.84 |
| rs8013756 | 12.16 | 11.47 | 77.78 | 20.13 | 2.09 | 77.52 | 22.02 | 0.46 |
| rs1032936 | 31.44 | 28.56 | 46.93 | 43.27 | 9.80 | 52.29 | 38.30 | 9.40 |
| rs1254601 | 43.20 | 41.86 | 31.50 | 50.59 | 17.91 | 34.86 | 46.56 | 18.58 |
| rs7145029 | 16.47 | 17.66 | 69.80 | 27.45 | 2.75 | 67.43 | 29.82 | 2.75 |
| rs10498475 | 6.93 | 6.42 | 86.54 | 13.07 | 0.39 | 87.39 | 12.39 | 0.23 |
| rs17126074 | 1.05 | 0.57 | 97.91 | 2.09 | 0.00 | 98.85 | 1.15 | 0.00 |
| rs8004624 | 49.02 | 48.74 | 26.27 | 49.41 | 24.31 | 25.46 | 46.56 | 27.98 |
| rs6572868 | 10.33 | 10.32 | 80.13 | 19.08 | 0.78 | 80.73 | 17.89 | 1.38 |
| rs3825596 | 7.78 | 8.49 | 84.84 | 14.77 | 0.39 | 83.72 | 15.60 | 0.69 |
| rs17127622 | 6.21 | 4.24 | 87.97 | 11.63 | 0.39 | 91.97 | 7.57 | 0.46 |
| rs762063 | 43.46 | 39.91 | 32.42 | 48.24 | 19.35 | 34.40 | 51.38 | 14.22 |
| rs12889199 | 7.65 | 7.00 | 85.36 | 13.99 | 0.65 | 86.70 | 12.61 | 0.69 |
| rs17253619 | 10.85 | 11.93 | 79.74 | 18.82 | 1.44 | 77.06 | 22.02 | 0.92 |
| rs2357947 | 10.07 | 10.67 | 80.78 | 18.30 | 0.92 | 80.05 | 18.58 | 1.38 |
| rs11622740 | 35.69 | 32.91 | 41.57 | 45.49 | 12.94 | 46.33 | 41.51 | 12.16 |
| rs946615 | 26.27 | 24.31 | 53.59 | 40.26 | 6.14 | 58.03 | 35.32 | 6.65 |
| rs2277495 | 31.63 | 29.13 | 47.45 | 41.83 | 10.72 | 50.92 | 39.91 | 9.17 |
| rs3751464 | 19.61 | 25.92 | 64.71 | 31.37 | 3.92 | 55.05 | 38.07 | 6.88 |
| rs17127595 | 13.66 | 15.02 | 74.25 | 24.18 | 1.57 | 72.48 | 25.00 | 2.52 |
| rs1695 | 30.72 | 31.42 | 48.50 | 41.57 | 9.93 | 46.56 | 44.04 | 9.40 |
| rs11431 | 48.56 | 48.05 | 24.58 | 53.73 | 21.70 | 24.77 | 54.36 | 20.87 |
| rs2075598 | 1.76 | 1.03 | 96.47 | 3.53 | 0.00 | 97.94 | 2.06 | 0.00 |
| rs4901200 | 13.07 | 11.93 | 75.82 | 22.22 | 1.96 | 77.29 | 21.56 | 1.15 |
| rs7150275 | 22.81 | 23.39 | 59.87 | 34.64 | 5.49 | 60.09 | 33.03 | 6.88 |
| rs10141001 | 14.18 | 12.96 | 73.73 | 24.18 | 2.09 | 75.46 | 23.17 | 1.38 |
| rs3794042 | 39.41 | 39.68 | 37.78 | 45.62 | 16.60 | 36.47 | 47.71 | 15.83 |
| rs1874569 | 25.29 | 24.43 | 54.90 | 39.61 | 5.49 | 58.03 | 35.09 | 6.88 |
| rs2509712 | 18.43 | 20.30 | 66.14 | 30.85 | 3.01 | 62.16 | 35.09 | 2.75 |
| rs7149810 | 23.14 | 23.97 | 59.74 | 34.25 | 6.01 | 59.40 | 33.26 | 7.34 |
| rs7167 | 22.81 | 23.05 | 60.39 | 33.59 | 6.01 | 59.17 | 35.55 | 5.28 |
| rs3742536 | 22.29 | 21.33 | 59.87 | 35.69 | 4.44 | 61.24 | 34.86 | 3.90 |
| rs12895034 | 33.86 | 33.94 | 44.31 | 43.66 | 12.03 | 44.95 | 42.20 | 12.84 |
| rs17694496 | 21.63 | 18.12 | 60.52 | 35.69 | 3.79 | 67.66 | 28.44 | 3.90 |
| rs1201378 | 40.33 | 39.91 | 34.90 | 49.54 | 15.56 | 38.30 | 43.58 | 18.12 |
| rs803012 | 22.68 | 21.67 | 59.61 | 35.42 | 4.97 | 60.78 | 35.09 | 4.13 |
| rs708502 | 14.12 | 13.88 | 73.59 | 24.58 | 1.83 | 73.39 | 25.46 | 1.15 |
| rs1209087 | 40.33 | 40.71 | 35.03 | 49.28 | 15.69 | 37.84 | 42.89 | 19.27 |
| rs17125273 | 15.36 | 17.20 | 71.63 | 26.01 | 2.35 | 70.18 | 25.23 | 4.59 |
| rs1565970 | 7.12 | 6.19 | 86.14 | 13.46 | 0.39 | 87.84 | 11.93 | 0.23 |
| rs1957844 | 20.52 | 22.02 | 63.01 | 32.94 | 4.05 | 61.01 | 33.94 | 5.05 |
| rs17666653 | 20.33 | 22.13 | 63.66 | 32.03 | 4.31 | 59.40 | 36.93 | 3.67 |
| rs17128136 | 9.22 | 10.32 | 82.88 | 15.82 | 1.31 | 81.42 | 16.51 | 2.06 |
| rs12587410 | 3.27 | 3.78 | 93.73 | 6.01 | 0.26 | 92.66 | 7.11 | 0.23 |
| rs17831682 | 7.39 | 9.63 | 85.23 | 14.77 | 0.00 | 82.34 | 16.06 | 1.61 |
| rs17666689 | 16.08 | 17.43 | 70.46 | 26.93 | 2.61 | 68.58 | 27.98 | 3.44 |
| rs708486 | 45.82 | 48.62 | 28.63 | 51.11 | 20.26 | 28.21 | 46.33 | 25.46 |
| rs9671722 | 16.01 | 17.09 | 71.24 | 25.49 | 3.27 | 69.50 | 26.83 | 3.67 |
| rs1953861 | 38.24 | 41.28 | 37.78 | 47.97 | 14.25 | 34.63 | 48.17 | 17.20 |
| rs17831675 | 7.25 | 9.63 | 85.62 | 14.25 | 0.13 | 82.11 | 16.51 | 1.38 |
| rs1993839 | 19.67 | 80.85 | 64.31 | 32.03 | 3.66 | 65.60 | 30.50 | 3.90 |
| rs555835 | 42.22 | 39.91 | 33.33 | 48.89 | 17.78 | 36.24 | 47.71 | 16.06 |
| rs545659 | 23.46 | 23.17 | 59.48 | 34.12 | 6.41 | 59.40 | 34.86 | 5.73 |

*0 denotes the homozygote genotypes for the frequent allele, 1 is heterozygote; 2 homozygote genotype for the minor allele; MAF: minor allele frequency
