# Supplementary material for: Evaluation of a Partial Genome Screening of Two Asthma Susceptibility Regions Using Bayesian Network Based Bayesian Multilevel Analysis of Relevance
Source: PLoS One. 2012 Mar 14;7(3):e33573. doi: 10.1371/journal.pone.0033573 (PMC3303848; doi:10.1371/journal.pone.0033573)
Supplement: Table S3 — Statistical evaluation for association of SNPs with asthma at allele and genotype levels. (DOC) [file pone.0033573.s007.doc]

**Table S3. Statistical evaluation for association of SNPs with asthma at allele and genotype levels**

| **SNP** | **Tests for deviation from Hardy-Weinberg equilibrium** | | **Tests for association (95% confidence interval)** | | | | |
| --- | --- | --- | --- | --- | --- | --- | --- |
|  | **Controls** | **Cases** | **allele freq. difference** | **heterozygous** | **homozygous** | **allele positivity** | **Armitage's trend test** |
| rs2513081 | 0.908 | 1.000 | **Risk allele 2** | | | | |
|  |  |  |  |  |  |  |  |
|  |  |  |  |  |  |  |  |
|  |  |  | **[1]<->[2]** | **[11]<->[12]** | **[11+]<->[22]** | **[11]<->[12+22]** | **odds ratio** |
|  |  |  | OR=1.002 | OR=1.013 | OR=0.971 | OR=1.008 | OR=0.998 |
|  |  |  | C.I.=[0.812-1.237] | C.I.=[0.784-1.308] | C.I.=[0.518-1.819] | C.I.=[0.788-1.290] |  |
|  |  |  | chi2=0.00 | chi2=0.01 | chi2=0.01 | chi2=0.00 | chi2=0.00 |
|  |  |  | p=0.98366 (P) | p=0.92151 | p=0.92646 | p=0.94712 | p=0.98366 |
|  |  |  | **Risk allele 1** | | | | |
|  |  |  | **[2]<->[1]** | **[22]<->[12]** | **[22]<->[11]** | **[11+12]<->[22]** | **odds ratio** |
|  |  |  | OR=0.998 | OR=1.043 | OR=1.030 | OR=1.034 | OR=1.002 |
|  |  |  | C.I.=[0.808-1.232] | C.I.=[0.547-1.990] | C.I.=[0.550-1.929] | C.I.=[0.555-1.927] |  |
|  |  |  | chi2=0.00 | chi2=0.02 | chi2=0.01 | chi2=0.01 | chi2=0.00 |
|  |  |  | p=0.98366 (P) | p=0.89752 | p=0.92646 | p=0.91535 | p=0.98366 |
| rs7118247 | 0.170 | 0.611 | **Risk allele 2** | | | | |
|  |  |  |  |  |  |  |  |
|  |  |  |  |  |  |  |  |
|  |  |  | **[1]<->[2]** | **[11]<->[12]** | **[11+]<->[22]** | **[11]<->[12+22]** | **odds ratio** |
|  |  |  | OR=0.831 | OR=0.862 | OR=0.643 | OR=0.832 | OR=0.829 |
|  |  |  | C.I.=[0.669-1.033] | C.I.=[0.663-1.121] | C.I.=[0.341-1.211] | C.I.=[0.647-1.071] |  |
|  |  |  | chi2=2.79 | chi2=1.22 | chi2=1.90 | chi2=2.04 | chi2=2.68 |
|  |  |  | p=0.09477 (P) | p=0.26842 | p=0.16830 | p=0.15342 | p=0.10184 |
|  |  |  | **Risk allele 1** | | | | |
|  |  |  | **[2]<->[1]** | **[22]<->[12]** | **[22]<->[11]** | **[11+12]<->[22]** | **odds ratio** |
|  |  |  | OR=1.203 | OR=1.342 | OR=1.556 | OR=1.489 | OR=1.210 |
|  |  |  | C.I.=[0.968-1.495] | C.I.=[0.696-2.584] | C.I.=[0.826-2.933] | C.I.=[0.794-2.792] |  |
|  |  |  | chi2=2.79 | chi2=0.78 | chi2=1.90 | chi2=1.56 | chi2=2.68 |
|  |  |  | p=0.09477 (P) | p=0.37839 | p=0.16830 | p=0.21233 | p=0.10184 |
| rs10750931 | 0.779 | 0.725 | **Risk allele 2** | | | | |
|  |  |  |  |  |  |  |  |
|  |  |  |  |  |  |  |  |
|  |  |  | **[1]<->[2]** | **[11]<->[12]** | **[11+]<->[22]** | **[11]<->[12+22]** | **odds ratio** |
|  |  |  | OR=1.085 | OR=1.096 | OR=1.127 | OR=1.099 | OR=1.082 |
|  |  |  | C.I.=[0.863-1.363] | C.I.=[0.841-1.430] | C.I.=[0.505-2.514] | C.I.=[0.849-1.423] |  |
|  |  |  | chi2=0.49 | chi2=0.46 | chi2=0.09 | chi2=0.51 | chi2=0.50 |
|  |  |  | p=0.48509 (P) | p=0.49697 | p=0.77005 | p=0.47501 | p=0.48115 |
|  |  |  | **Risk allele 1** | | | | |
|  |  |  | **[2]<->[1]** | **[22]<->[12]** | **[22]<->[11]** | **[11+12]<->[22]** | **odds ratio** |
|  |  |  | OR=0.922 | OR=0.973 | OR=0.887 | OR=0.910 | OR=0.924 |
|  |  |  | C.I.=[0.734-1.158] | C.I.=[0.428-2.213] | C.I.=[0.398-1.979] | C.I.=[0.409-2.023] |  |
|  |  |  | chi2=0.49 | chi2=0.00 | chi2=0.09 | chi2=0.05 | chi2=0.50 |
|  |  |  | p=0.48509 (P) | p=0.94768 | p=0.77005 | p=0.81701 | p=0.48115 |
| rs528823 | 1.000 | 0.431 | **Risk allele 2** | | | | |
|  |  |  |  |  |  |  |  |
|  |  |  |  |  |  |  |  |
|  |  |  | **[1]<->[2]** | **[11]<->[12]** | **[11+]<->[22]** | **[11]<->[12+22]** | **odds ratio** |
|  |  |  | OR=0.914 | OR=0.863 | OR=0.894 | OR=0.869 | OR=0.927 |
|  |  |  | C.I.=[0.764-1.093] | C.I.=[0.673-1.107] | C.I.=[0.598-1.336] | C.I.=[0.687-1.100] |  |
|  |  |  | chi2=0.97 | chi2=1.34 | chi2=0.30 | chi2=1.36 | chi2=0.95 |
|  |  |  | p=0.32547 (P) | p=0.24661 | p=0.58450 | p=0.24317 | p=0.32934 |
|  |  |  | **Risk allele 1** | | | | |
|  |  |  | **[2]<->[1]** | **[22]<->[12]** | **[22]<->[11]** | **[11+12]<->[22]** | **odds ratio** |
|  |  |  | OR=1.094 | OR=0.965 | OR=1.119 | OR=1.043 | OR=1.078 |
|  |  |  | C.I.=[0.915-1.309] | C.I.=[0.643-1.449] | C.I.=[0.749-1.671] | C.I.=[0.710-1.532] |  |
|  |  |  | chi2=0.97 | chi2=0.03 | chi2=0.30 | chi2=0.05 | chi2=0.95 |
|  |  |  | p=0.32547 (P) | p=0.86495 | p=0.58450 | p=0.82929 | p=0.32934 |
| rs2302360 | 0.051 | 1.000 | **Risk allele 2** | | | | |
|  |  |  |  |  |  |  |  |
|  |  |  |  |  |  |  |  |
|  |  |  | **[1]<->[2]** | **[11]<->[12]** | **[11+]<->[22]** | **[11]<->[12+22]** | **odds ratio** |
|  |  |  | OR=0.978 | OR=1.077 | OR=0.766 | OR=1.028 | OR=0.950 |
|  |  |  | C.I.=[0.797-1.199] | C.I.=[0.835-1.390] | C.I.=[0.438-1.338] | C.I.=[0.806-1.312] |  |
|  |  |  | chi2=0.05 | chi2=0.33 | chi2=0.88 | chi2=0.05 | chi2=0.04 |
|  |  |  | p=0.83067 (P) | p=0.56718 | p=0.34701 | p=0.82156 | p=0.83449 |
|  |  |  | **Risk allele 1** | | | | |
|  |  |  | **[2]<->[1]** | **[22]<->[12]** | **[22]<->[11]** | **[11+12]<->[22]** | **odds ratio** |
|  |  |  | OR=1.023 | OR=1.407 | OR=1.306 | OR=1.339 | OR=1.058 |
|  |  |  | C.I.=[0.834-1.254] | C.I.=[0.791-2.504] | C.I.=[0.748-2.282] | C.I.=[0.772-2.325] |  |
|  |  |  | chi2=0.05 | chi2=1.36 | chi2=0.88 | chi2=1.09 | chi2=0.04 |
|  |  |  | p=0.83067 (P) | p=0.24408 | p=0.34701 | p=0.29747 | p=0.83449 |
| rs540170 | 0.663 | 0.250 | **Risk allele 2** | | | | |
|  |  |  |  |  |  |  |  |
|  |  |  |  |  |  |  |  |
|  |  |  | **[1]<->[2]** | **[11]<->[12]** | **[11+]<->[22]** | **[11]<->[12+22]** | **odds ratio** |
|  |  |  | OR=1.138 | OR=1.242 | OR=1.295 | OR=1.257 | OR=1.141 |
|  |  |  | C.I.=[0.963-1.344] | C.I.=[0.936-1.647] | C.I.=[0.920-1.823] | C.I.=[0.961-1.644] |  |
|  |  |  | chi2=2.31 | chi2=2.26 | chi2=2.20 | chi2=2.80 | chi2=2.38 |
|  |  |  | p=0.12879 (P) | p=0.13287 | p=0.13788 | p=0.09424 | p=0.12276 |
|  |  |  | **Risk allele 1** | | | | |
|  |  |  | **[2]<->[1]** | **[22]<->[12]** | **[22]<->[11]** | **[11+12]<->[22]** | **odds ratio** |
|  |  |  | OR=0.879 | OR=0.959 | OR=0.772 | OR=0.891 | OR=0.877 |
|  |  |  | C.I.=[0.744-1.038] | C.I.=[0.710-1.295] | C.I.=[0.549-1.087] | C.I.=[0.670-1.185] |  |
|  |  |  | chi2=2.31 | chi2=0.08 | chi2=2.20 | chi2=0.63 | chi2=2.38 |
|  |  |  | p=0.12879 (P) | p=0.78336 | p=0.13788 | p=0.42584 | p=0.12276 |
| rs7127662 | 0.601 | 0.757 | **Risk allele 2** | | | | |
|  |  |  |  |  |  |  |  |
|  |  |  |  |  |  |  |  |
|  |  |  | **[1]<->[2]** | **[11]<->[12]** | **[11+]<->[22]** | **[11]<->[12+22]** | **odds ratio** |
|  |  |  | OR=0.832 | OR=0.837 | OR=0.692 | OR=0.817 | OR=0.834 |
|  |  |  | C.I.=[0.676-1.024] | C.I.=[0.648-1.081] | C.I.=[0.385-1.245] | C.I.=[0.640-1.045] |  |
|  |  |  | chi2=3.02 | chi2=1.87 | chi2=1.52 | chi2=2.60 | chi2=2.96 |
|  |  |  | p=0.08238 (P) | p=0.17157 | p=0.21708 | p=0.10687 | p=0.08522 |
|  |  |  | **Risk allele 1** | | | | |
|  |  |  | **[2]<->[1]** | **[22]<->[12]** | **[22]<->[11]** | **[11+12]<->[22]** | **odds ratio** |
|  |  |  | OR=1.202 | OR=1.208 | OR=1.444 | OR=1.360 | OR=1.199 |
|  |  |  | C.I.=[0.977-1.479] | C.I.=[0.660-2.212] | C.I.=[0.804-2.595] | C.I.=[0.761-2.429] |  |
|  |  |  | chi2=3.02 | chi2=0.38 | chi2=1.52 | chi2=1.09 | chi2=2.96 |
|  |  |  | p=0.08238 (P) | p=0.53934 | p=0.21708 | p=0.29733 | p=0.08522 |
| rs2847204 | 0.113 | 1.000 | **Risk allele 2** | | | | |
|  |  |  |  |  |  |  |  |
|  |  |  |  |  |  |  |  |
|  |  |  | **[1]<->[2]** | **[11]<->[12]** | **[11+]<->[22]** | **[11]<->[12+22]** | **odds ratio** |
|  |  |  | OR=0.832 | OR=0.899 | OR=0.471 | OR=0.857 | OR=0.816 |
|  |  |  | C.I.=[0.643-1.077] | C.I.=[0.671-1.204] | C.I.=[0.173-1.279] | C.I.=[0.645-1.138] |  |
|  |  |  | chi2=1.95 | chi2=0.51 | chi2=2.28 | chi2=1.14 | chi2=1.88 |
|  |  |  | p=0.16310 (P) | p=0.47513 | p=0.13095 | p=0.28541 | p=0.17053 |
|  |  |  | **Risk allele 1** | | | | |
|  |  |  | **[2]<->[1]** | **[22]<->[12]** | **[22]<->[11]** | **[11+12]<->[22]** | **odds ratio** |
|  |  |  | OR=1.201 | OR=1.910 | OR=2.124 | OR=2.077 | OR=1.246 |
|  |  |  | C.I.=[0.928-1.555] | C.I.=[0.686-5.319] | C.I.=[0.782-5.773] | C.I.=[0.766-5.634] |  |
|  |  |  | chi2=1.95 | chi2=1.58 | chi2=2.28 | chi2=2.15 | chi2=1.88 |
|  |  |  | p=0.16310 (P) | p=0.20912 | p=0.13095 | p=0.14249 | p=0.17053 |
| rs1567083 | 0.560 | 0.829 | **Risk allele 2** | | | | |
|  |  |  |  |  |  |  |  |
|  |  |  |  |  |  |  |  |
|  |  |  | **[1]<->[2]** | **[11]<->[12]** | **[11+]<->[22]** | **[11]<->[12+22]** | **odds ratio** |
|  |  |  | OR=0.842 | OR=0.858 | OR=0.653 | OR=0.840 | OR=0.838 |
|  |  |  | C.I.=[0.658-1.076] | C.I.=[0.647-1.137] | C.I.=[0.270-1.580] | C.I.=[0.639-1.104] |  |
|  |  |  | chi2=1.89 | chi2=1.14 | chi2=0.91 | chi2=1.57 | chi2=1.86 |
|  |  |  | p=0.16939 (P) | p=0.28620 | p=0.34136 | p=0.21077 | p=0.17265 |
|  |  |  | **Risk allele 1** | | | | |
|  |  |  | **[2]<->[1]** | **[22]<->[12]** | **[22]<->[11]** | **[11+12]<->[22]** | **odds ratio** |
|  |  |  | OR=1.188 | OR=1.313 | OR=1.531 | OR=1.477 | OR=1.196 |
|  |  |  | C.I.=[0.929-1.519] | C.I.=[0.530-3.254] | C.I.=[0.633-3.704] | C.I.=[0.612-3.564] |  |
|  |  |  | chi2=1.89 | chi2=0.35 | chi2=0.91 | chi2=0.76 | chi2=1.86 |
|  |  |  | p=0.16939 (P) | p=0.55498 | p=0.34136 | p=0.38296 | p=0.17265 |
| rs7925087 | 0.792 | 1.000 | **Risk allele 2** | | | | |
|  |  |  |  |  |  |  |  |
|  |  |  |  |  |  |  |  |
|  |  |  | **[1]<->[2]** | **[11]<->[12]** | **[11+]<->[22]** | **[11]<->[12+22]** | **odds ratio** |
|  |  |  | OR=0.780 | OR=0.778 | OR=0.565 | OR=0.772 | OR=0.775 |
|  |  |  | C.I.=[0.556-1.095] | C.I.=[0.544-1.113] | C.I.=[0.059-5.454] | C.I.=[0.542-1.101] |  |
|  |  |  | chi2=2.07 | chi2=1.90 | chi2=0.25 | chi2=2.05 | chi2=2.12 |
|  |  |  | p=0.15014 (P) | p=0.16824 | p=0.61724 | p=0.15211 | p=0.14537 |
|  |  |  | **Risk allele 1** | | | | |
|  |  |  | **[2]<->[1]** | **[22]<->[12]** | **[22]<->[11]** | **[11+12]<->[22]** | **odds ratio** |
|  |  |  | OR=1.282 | OR=1.376 | OR=1.769 | OR=1.713 | OR=1.292 |
|  |  |  | C.I.=[0.913-1.798] | C.I.=[0.140-13.559] | C.I.=[0.183-17.064] | C.I.=[0.178-16.515] |  |
|  |  |  | chi2=2.07 | chi2=0.08 | chi2=0.25 | chi2=0.22 | chi2=2.12 |
|  |  |  | p=0.15014 (P) | p=0.78360 | p=0.61724 | p=0.63772 | p=0.14537 |
| rs2074422 | 0.647 | 0.774 | **Risk allele 2** | | | | |
|  |  |  |  |  |  |  |  |
|  |  |  |  |  |  |  |  |
|  |  |  | **[1]<->[2]** | **[11]<->[12]** | **[11+]<->[22]** | **[11]<->[12+22]** | **odds ratio** |
|  |  |  | OR=1.104 | OR=1.108 | OR=1.219 | OR=1.119 | OR=1.106 |
|  |  |  | C.I.=[0.899-1.356] | C.I.=[0.861-1.425] | C.I.=[0.659-2.255] | C.I.=[0.877-1.427] |  |
|  |  |  | chi2=0.89 | chi2=0.63 | chi2=0.40 | chi2=0.82 | chi2=0.91 |
|  |  |  | p=0.34522 (P) | p=0.42572 | p=0.52658 | p=0.36547 | p=0.34022 |
|  |  |  | **Risk allele 1** | | | | |
|  |  |  | **[2]<->[1]** | **[22]<->[12]** | **[22]<->[11]** | **[11+12]<->[22]** | **odds ratio** |
|  |  |  | OR=0.906 | OR=0.909 | OR=0.820 | OR=0.850 | OR=0.904 |
|  |  |  | C.I.=[0.737-1.113] | C.I.=[0.484-1.706] | C.I.=[0.444-1.516] | C.I.=[0.462-1.561] |  |
|  |  |  | chi2=0.89 | chi2=0.09 | chi2=0.40 | chi2=0.28 | chi2=0.91 |
|  |  |  | p=0.34522 (P) | p=0.76538 | p=0.52658 | p=0.59912 | p=0.34022 |
| rs488483 | 0.263 | 0.648 | **Risk allele 2** | | | | |
|  |  |  |  |  |  |  |  |
|  |  |  |  |  |  |  |  |
|  |  |  | **[1]<->[2]** | **[11]<->[12]** | **[11+]<->[22]** | **[11]<->[12+22]** | **odds ratio** |
|  |  |  | OR=0.953 | OR=0.880 | OR=1.173 | OR=0.906 | OR=0.985 |
|  |  |  | C.I.=[0.773-1.174] | C.I.=[0.682-1.135] | C.I.=[0.632-2.177] | C.I.=[0.709-1.159] |  |
|  |  |  | chi2=0.20 | chi2=0.97 | chi2=0.25 | chi2=0.61 | chi2=0.21 |
|  |  |  | p=0.65165 (P) | p=0.32438 | p=0.61373 | p=0.43314 | p=0.64824 |
|  |  |  | **Risk allele 1** | | | | |
|  |  |  | **[2]<->[1]** | **[22]<->[12]** | **[22]<->[11]** | **[11+12]<->[22]** | **odds ratio** |
|  |  |  | OR=1.049 | OR=0.750 | OR=0.853 | OR=0.817 | OR=1.018 |
|  |  |  | C.I.=[0.852-1.293] | C.I.=[0.397-1.417] | C.I.=[0.459-1.583] | C.I.=[0.443-1.508] |  |
|  |  |  | chi2=0.20 | chi2=0.79 | chi2=0.25 | chi2=0.42 | chi2=0.21 |
|  |  |  | p=0.65165 (P) | p=0.37472 | p=0.61373 | p=0.51742 | p=0.64824 |
| rs7935803 | 0.115 | 0.022 | **Risk allele 2** | | | | |
|  |  |  |  |  |  |  |  |
|  |  |  |  |  |  |  |  |
|  |  |  | **[1]<->[2]** | **[11]<->[12]** | **[11+]<->[22]** | **[11]<->[12+22]** | **odds ratio** |
|  |  |  | OR=1.193 | OR=1.084 | OR=1.447 | OR=1.173 | OR=1.190 |
|  |  |  | C.I.=[1.006-1.416] | C.I.=[0.837-1.404] | C.I.=[1.027-2.038] | C.I.=[0.923-1.492] |  |
|  |  |  | chi2=4.11 | chi2=0.37 | chi2=4.48 | chi2=1.70 | chi2=3.80 |
|  |  |  | p=0.04275 (P) | p=0.54080 | p=0.03429 | p=0.19208 | p=0.05114 |
|  |  |  | **Risk allele 1** | | | | |
|  |  |  | **[2]<->[1]** | **[22]<->[12]** | **[22]<->[11]** | **[11+12]<->[22]** | **odds ratio** |
|  |  |  | OR=0.838 | OR=0.749 | OR=0.691 | OR=0.720 | OR=0.842 |
|  |  |  | C.I.=[0.706-0.994] | C.I.=[0.534-1.052] | C.I.=[0.491-0.974] | C.I.=[0.525-0.988] |  |
|  |  |  | chi2=4.11 | chi2=2.79 | chi2=4.48 | chi2=4.17 | chi2=3.80 |
|  |  |  | p=0.04275 (P) | p=0.09508 | p=0.03429 | p=0.04123 | p=0.05114 |
| rs12422200 | 0.838 | 0.696 | **Risk allele 2** | | | | |
|  |  |  |  |  |  |  |  |
|  |  |  |  |  |  |  |  |
|  |  |  | **[1]<->[2]** | **[11]<->[12]** | **[11+]<->[22]** | **[11]<->[12+22]** | **odds ratio** |
|  |  |  | OR=1.064 | OR=1.022 | OR=1.239 | OR=1.049 | OR=1.080 |
|  |  |  | C.I.=[0.875-1.294] | C.I.=[0.796-1.311] | C.I.=[0.741-2.072] | C.I.=[0.826-1.331] |  |
|  |  |  | chi2=0.39 | chi2=0.03 | chi2=0.67 | chi2=0.15 | chi2=0.39 |
|  |  |  | p=0.53151 (P) | p=0.86574 | p=0.41342 | p=0.69575 | p=0.53131 |
|  |  |  | **Risk allele 1** | | | | |
|  |  |  | **[2]<->[1]** | **[22]<->[12]** | **[22]<->[11]** | **[11+12]<->[22]** | **odds ratio** |
|  |  |  | OR=0.940 | OR=0.825 | OR=0.807 | OR=0.814 | OR=0.928 |
|  |  |  | C.I.=[0.773-1.142] | C.I.=[0.486-1.399] | C.I.=[0.483-1.350] | C.I.=[0.491-1.349] |  |
|  |  |  | chi2=0.39 | chi2=0.51 | chi2=0.67 | chi2=0.64 | chi2=0.39 |
|  |  |  | p=0.53151 (P) | p=0.47407 | p=0.41342 | p=0.42344 | p=0.53131 |
| rs7928208 | 0.035 | 1.000 | **Risk allele 2** | | | | |
|  |  |  |  |  |  |  |  |
|  |  |  |  |  |  |  |  |
|  |  |  | **[1]<->[2]** | **[11]<->[12]** | **[11+]<->[22]** | **[11]<->[12+22]** | **odds ratio** |
|  |  |  | OR=1.782 | OR=2.087 | OR=0.363 | OR=1.943 | OR=1.874 |
|  |  |  | C.I.=[1.076-2.953] | C.I.=[1.228-3.545] | C.I.=[0.017-7.585] | C.I.=[1.154-3.270] |  |
|  |  |  | chi2=5.16 | chi2=7.69 | chi2=1.10 | chi2=6.45 | chi2=4.97 |
|  |  |  | p=0.02307 (P) | p=0.00556 | p=0.29438 | p=0.01112 | p=0.02585 |
|  |  |  | **Risk allele 1** | | | | |
|  |  |  | **[2]<->[1]** | **[22]<->[12]** | **[22]<->[11]** | **[11+12]<->[22]** | **odds ratio** |
|  |  |  | OR=0.561 | OR=5.727 | OR=2.753 | OR=2.859 | OR=0.683 |
|  |  |  | C.I.=[0.339-0.930] | C.I.=[0.263-124.510] | C.I.=[0.132-57.480] | C.I.=[0.137-59.677] |  |
|  |  |  | chi2=5.16 | chi2=2.21 | chi2=1.10 | chi2=1.14 | chi2=4.97 |
|  |  |  | p=0.02307 (P) | p=0.13697 | p=0.29438 | p=0.28528 | p=0.02585 |
| rs2237997 | 0.584 | 0.389 | **Risk allele 2** | | | | |
|  |  |  |  |  |  |  |  |
|  |  |  |  |  |  |  |  |
|  |  |  | **[1]<->[2]** | **[11]<->[12]** | **[11+]<->[22]** | **[11]<->[12+22]** | **odds ratio** |
|  |  |  | OR=0.885 | OR=0.916 | OR=0.747 | OR=0.881 | OR=0.875 |
|  |  |  | C.I.=[0.743-1.054] | C.I.=[0.714-1.176] | C.I.=[0.501-1.114] | C.I.=[0.694-1.118] |  |
|  |  |  | chi2=1.87 | chi2=0.47 | chi2=2.05 | chi2=1.09 | chi2=1.93 |
|  |  |  | p=0.17114 (P) | p=0.49310 | p=0.15213 | p=0.29621 | p=0.16461 |
|  |  |  | **Risk allele 1** | | | | |
|  |  |  | **[2]<->[1]** | **[22]<->[12]** | **[22]<->[11]** | **[11+12]<->[22]** | **odds ratio** |
|  |  |  | OR=1.130 | OR=1.227 | OR=1.339 | OR=1.278 | OR=1.144 |
|  |  |  | C.I.=[0.948-1.346] | C.I.=[0.826-1.823] | C.I.=[0.897-1.998] | C.I.=[0.876-1.866] |  |
|  |  |  | chi2=1.87 | chi2=1.03 | chi2=2.05 | chi2=1.63 | chi2=1.93 |
|  |  |  | p=0.17114 (P) | p=0.31111 | p=0.15213 | p=0.20193 | p=0.16461 |
| rs3741240 | 0.205 | 0.248 | **Risk allele 2** | | | | |
|  |  |  |  |  |  |  |  |
|  |  |  |  |  |  |  |  |
|  |  |  | **[1]<->[2]** | **[11]<->[12]** | **[11+]<->[22]** | **[11]<->[12+22]** | **odds ratio** |
|  |  |  | OR=0.999 | OR=1.178 | OR=0.866 | OR=1.104 | OR=0.971 |
|  |  |  | C.I.=[0.839-1.189] | C.I.=[0.916-1.514] | C.I.=[0.588-1.277] | C.I.=[0.870-1.402] |  |
|  |  |  | chi2=0.00 | chi2=1.63 | chi2=0.53 | chi2=0.66 | chi2=0.00 |
|  |  |  | p=0.99121 (P) | p=0.20164 | p=0.46819 | p=0.41596 | p=0.99124 |
|  |  |  | **Risk allele 1** | | | | |
|  |  |  | **[2]<->[1]** | **[22]<->[12]** | **[22]<->[11]** | **[11+12]<->[22]** | **odds ratio** |
|  |  |  | OR=1.001 | OR=1.360 | OR=1.154 | OR=1.258 | OR=1.031 |
|  |  |  | C.I.=[0.841-1.191] | C.I.=[0.927-1.995] | C.I.=[0.783-1.702] | C.I.=[0.873-1.811] |  |
|  |  |  | chi2=0.00 | chi2=2.48 | chi2=0.53 | chi2=1.52 | chi2=0.00 |
|  |  |  | p=0.99121 (P) | p=0.11557 | p=0.46819 | p=0.21723 | p=0.99124 |
| rs11827029 | 0.012 | 1.000 | **Risk allele 2** | | | | |
|  |  |  |  |  |  |  |  |
|  |  |  |  |  |  |  |  |
|  |  |  | **[1]<->[2]** | **[11]<->[12]** | **[11+]<->[22]** | **[11]<->[12+22]** | **odds ratio** |
|  |  |  | OR=0.645 | OR=0.750 | OR=0.155 | OR=0.690 | OR=0.699 |
|  |  |  | C.I.=[0.404-1.028] | C.I.=[0.462-1.219] | C.I.=[0.009-2.818] | C.I.=[0.427-1.115] |  |
|  |  |  | chi2=3.45 | chi2=1.35 | chi2=2.92 | chi2=2.32 | chi2=3.21 |
|  |  |  | p=0.06333 (P) | p=0.24473 | p=0.08773 | p=0.12749 | p=0.07307 |
|  |  |  | **Risk allele 1** | | | | |
|  |  |  | **[2]<->[1]** | **[22]<->[12]** | **[22]<->[11]** | **[11+12]<->[22]** | **odds ratio** |
|  |  |  | OR=1.552 | OR=4.878 | OR=6.434 | OR=6.314 | OR=1.931 |
|  |  |  | C.I.=[0.973-2.475] | C.I.=[0.260-91.575] | C.I.=[0.355-116.658] | C.I.=[0.348-114.452] |  |
|  |  |  | chi2=3.45 | chi2=2.14 | chi2=2.92 | chi2=2.86 | chi2=3.21 |
|  |  |  | p=0.06333 (P) | p=0.14359 | p=0.08773 | p=0.09072 | p=0.07307 |
| rs11231128 | 0.518 | 1.000 | **Risk allele 2** | | | | |
|  |  |  |  |  |  |  |  |
|  |  |  |  |  |  |  |  |
|  |  |  | **[1]<->[2]** | **[11]<->[12]** | **[11+]<->[22]** | **[11]<->[12+22]** | **odds ratio** |
|  |  |  | OR=0.665 | OR=0.688 | OR=0.573 | OR=0.673 | OR=0.676 |
|  |  |  | C.I.=[0.384-1.152] | C.I.=[0.393-1.204] | C.I.=[0.023-14.100] | C.I.=[0.385-1.176] |  |
|  |  |  | chi2=2.14 | chi2=1.73 | chi2=0.58 | chi2=1.96 | chi2=2.13 |
|  |  |  | p=0.14330 (P) | p=0.18819 | p=0.44589 | p=0.16202 | p=0.14406 |
|  |  |  | **Risk allele 1** | | | | |
|  |  |  | **[2]<->[1]** | **[22]<->[12]** | **[22]<->[11]** | **[11+12]<->[22]** | **odds ratio** |
|  |  |  | OR=1.504 | OR=1.220 | OR=1.745 | OR=1.713 | OR=1.616 |
|  |  |  | C.I.=[0.868-2.606] | C.I.=[0.047-31.330] | C.I.=[0.071-42.932] | C.I.=[0.070-42.138] |  |
|  |  |  | chi2=2.14 | chi2=0.40 | chi2=0.58 | chi2=0.57 | chi2=2.13 |
|  |  |  | p=0.14330 (P) | p=0.52838 | p=0.44589 | p=0.45010 | p=0.14406 |
| rs954237 | 0.395 | 0.919 | **Risk allele 2** | | | | |
|  |  |  |  |  |  |  |  |
|  |  |  |  |  |  |  |  |
|  |  |  | **[1]<->[2]** | **[11]<->[12]** | **[11+]<->[22]** | **[11]<->[12+22]** | **odds ratio** |
|  |  |  | OR=1.011 | OR=1.053 | OR=0.994 | OR=1.039 | OR=1.005 |
|  |  |  | C.I.=[0.851-1.200] | C.I.=[0.816-1.359] | C.I.=[0.692-1.428] | C.I.=[0.817-1.321] |  |
|  |  |  | chi2=0.01 | chi2=0.16 | chi2=0.00 | chi2=0.10 | chi2=0.01 |
|  |  |  | p=0.90495 (P) | p=0.68961 | p=0.97333 | p=0.75656 | p=0.90602 |
|  |  |  | **Risk allele 1** | | | | |
|  |  |  | **[2]<->[1]** | **[22]<->[12]** | **[22]<->[11]** | **[11+12]<->[22]** | **odds ratio** |
|  |  |  | OR=0.990 | OR=1.060 | OR=1.006 | OR=1.035 | OR=0.996 |
|  |  |  | C.I.=[0.833-1.175] | C.I.=[0.743-1.512] | C.I.=[0.700-1.445] | C.I.=[0.740-1.447] |  |
|  |  |  | chi2=0.01 | chi2=0.10 | chi2=0.00 | chi2=0.04 | chi2=0.01 |
|  |  |  | p=0.90495 (P) | p=0.74848 | p=0.97333 | p=0.84225 | p=0.90602 |
| rs2905506 | 0.126 | 0.362 | **Risk allele 2** | | | | |
|  |  |  |  |  |  |  |  |
|  |  |  |  |  |  |  |  |
|  |  |  | **[1]<->[2]** | **[11]<->[12]** | **[11+]<->[22]** | **[11]<->[12+22]** | **odds ratio** |
|  |  |  | OR=1.068 | OR=1.085 | OR=1.097 | OR=1.087 | OR=1.059 |
|  |  |  | C.I.=[0.879-1.299] | C.I.=[0.843-1.396] | C.I.=[0.675-1.782] | C.I.=[0.855-1.380] |  |
|  |  |  | chi2=0.44 | chi2=0.40 | chi2=0.14 | chi2=0.46 | chi2=0.42 |
|  |  |  | p=0.50768 (P) | p=0.52723 | p=0.70993 | p=0.49612 | p=0.51819 |
|  |  |  | **Risk allele 1** | | | | |
|  |  |  | **[2]<->[1]** | **[22]<->[12]** | **[22]<->[11]** | **[11+12]<->[22]** | **odds ratio** |
|  |  |  | OR=0.936 | OR=0.989 | OR=0.912 | OR=0.940 | OR=0.944 |
|  |  |  | C.I.=[0.770-1.138] | C.I.=[0.598-1.635] | C.I.=[0.561-1.482] | C.I.=[0.583-1.513] |  |
|  |  |  | chi2=0.44 | chi2=0.00 | chi2=0.14 | chi2=0.07 | chi2=0.42 |
|  |  |  | p=0.50768 (P) | p=0.96630 | p=0.70993 | p=0.79765 | p=0.51819 |
| rs563748 | 0.457 | 0.784 | **Risk allele 2** | | | | |
|  |  |  |  |  |  |  |  |
|  |  |  |  |  |  |  |  |
|  |  |  | **[1]<->[2]** | **[11]<->[12]** | **[11+]<->[22]** | **[11]<->[12+22]** | **odds ratio** |
|  |  |  | OR=0.847 | OR=0.868 | OR=0.619 | OR=0.851 | OR=0.842 |
|  |  |  | C.I.=[0.641-1.119] | C.I.=[0.637-1.182] | C.I.=[0.196-1.957] | C.I.=[0.629-1.150] |  |
|  |  |  | chi2=1.37 | chi2=0.81 | chi2=0.68 | chi2=1.11 | chi2=1.34 |
|  |  |  | p=0.24260 (P) | p=0.36950 | p=0.40941 | p=0.29242 | p=0.24698 |
|  |  |  | **Risk allele 1** | | | | |
|  |  |  | **[2]<->[1]** | **[22]<->[12]** | **[22]<->[11]** | **[11+12]<->[22]** | **odds ratio** |
|  |  |  | OR=1.180 | OR=1.403 | OR=1.617 | OR=1.576 | OR=1.192 |
|  |  |  | C.I.=[0.894-1.559] | C.I.=[0.432-4.559] | C.I.=[0.511-5.115] | C.I.=[0.499-4.978] |  |
|  |  |  | chi2=1.37 | chi2=0.32 | chi2=0.68 | chi2=0.61 | chi2=1.34 |
|  |  |  | p=0.24260 (P) | p=0.57128 | p=0.40941 | p=0.43479 | p=0.24698 |
| rs10792269 | 0.697 | 0.138 | **Risk allele 2** | | | | |
|  |  |  |  |  |  |  |  |
|  |  |  |  |  |  |  |  |
|  |  |  | **[1]<->[2]** | **[11]<->[12]** | **[11+]<->[22]** | **[11]<->[12+22]** | **odds ratio** |
|  |  |  | OR=0.913 | OR=0.835 | OR=0.898 | OR=0.850 | OR=0.929 |
|  |  |  | C.I.=[0.767-1.087] | C.I.=[0.649-1.076] | C.I.=[0.623-1.295] | C.I.=[0.670-1.078] |  |
|  |  |  | chi2=1.05 | chi2=1.94 | chi2=0.33 | chi2=1.80 | chi2=1.01 |
|  |  |  | p=0.30598 (P) | p=0.16413 | p=0.56389 | p=0.17977 | p=0.31432 |
|  |  |  | **Risk allele 1** | | | | |
|  |  |  | **[2]<->[1]** | **[22]<->[12]** | **[22]<->[11]** | **[11+12]<->[22]** | **odds ratio** |
|  |  |  | OR=1.095 | OR=0.931 | OR=1.114 | OR=1.017 | OR=1.076 |
|  |  |  | C.I.=[0.920-1.303] | C.I.=[0.646-1.341] | C.I.=[0.772-1.606] | C.I.=[0.721-1.433] |  |
|  |  |  | chi2=1.05 | chi2=0.15 | chi2=0.33 | chi2=0.01 | chi2=1.01 |
|  |  |  | p=0.30598 (P) | p=0.69938 | p=0.56389 | p=0.92523 | p=0.31432 |
| rs12792791 | 0.349 | 0.814 | **Risk allele 2** | | | | |
|  |  |  |  |  |  |  |  |
|  |  |  |  |  |  |  |  |
|  |  |  | **[1]<->[2]** | **[11]<->[12]** | **[11+]<->[22]** | **[11]<->[12+22]** | **odds ratio** |
|  |  |  | OR=0.905 | OR=0.847 | OR=0.898 | OR=0.856 | OR=0.919 |
|  |  |  | C.I.=[0.754-1.087] | C.I.=[0.662-1.085] | C.I.=[0.579-1.395] | C.I.=[0.676-1.083] |  |
|  |  |  | chi2=1.14 | chi2=1.73 | chi2=0.23 | chi2=1.68 | chi2=1.16 |
|  |  |  | p=0.28623 (P) | p=0.18884 | p=0.63312 | p=0.19437 | p=0.28184 |
|  |  |  | **Risk allele 1** | | | | |
|  |  |  | **[2]<->[1]** | **[22]<->[12]** | **[22]<->[11]** | **[11+12]<->[22]** | **odds ratio** |
|  |  |  | OR=1.105 | OR=0.943 | OR=1.113 | OR=1.032 | OR=1.087 |
|  |  |  | C.I.=[0.920-1.327] | C.I.=[0.604-1.474] | C.I.=[0.717-1.728] | C.I.=[0.674-1.579] |  |
|  |  |  | chi2=1.14 | chi2=0.07 | chi2=0.23 | chi2=0.02 | chi2=1.16 |
|  |  |  | p=0.28623 (P) | p=0.79733 | p=0.63312 | p=0.88548 | p=0.28184 |
| rs7935186 | 0.014 | 1.000 | **Risk allele 2** | | | | |
|  |  |  |  |  |  |  |  |
|  |  |  |  |  |  |  |  |
|  |  |  | **[1]<->[2]** | **[11]<->[12]** | **[11+]<->[22]** | **[11]<->[12+22]** | **odds ratio** |
|  |  |  | OR=1.126 | OR=1.290 | OR=0.995 | OR=1.235 | OR=1.077 |
|  |  |  | C.I.=[0.931-1.362] | C.I.=[1.005-1.656] | C.I.=[0.623-1.589] | C.I.=[0.974-1.566] |  |
|  |  |  | chi2=1.49 | chi2=4.00 | chi2=0.00 | chi2=3.05 | chi2=1.41 |
|  |  |  | p=0.22266 (P) | p=0.04561 | p=0.98280 | p=0.08070 | p=0.23527 |
|  |  |  | **Risk allele 1** | | | | |
|  |  |  | **[2]<->[1]** | **[22]<->[12]** | **[22]<->[11]** | **[11+12]<->[22]** | **odds ratio** |
|  |  |  | OR=0.888 | OR=1.296 | OR=1.005 | OR=1.110 | OR=0.929 |
|  |  |  | C.I.=[0.734-1.075] | C.I.=[0.801-2.098] | C.I.=[0.629-1.605] | C.I.=[0.703-1.754] |  |
|  |  |  | chi2=1.49 | chi2=1.12 | chi2=0.00 | chi2=0.20 | chi2=1.41 |
|  |  |  | p=0.22266 (P) | p=0.28970 | p=0.98280 | p=0.65395 | p=0.23527 |
| rs2513044 | 0.004 | 0.257 | **Risk allele 2** | | | | |
|  |  |  |  |  |  |  |  |
|  |  |  |  |  |  |  |  |
|  |  |  | **[1]<->[2]** | **[11]<->[12]** | **[11+]<->[22]** | **[11]<->[12+22]** | **odds ratio** |
|  |  |  | OR=0.970 | OR=0.905 | OR=0.993 | OR=0.921 | OR=0.979 |
|  |  |  | C.I.=[0.817-1.153] | C.I.=[0.702-1.166] | C.I.=[0.672-1.466] | C.I.=[0.723-1.174] |  |
|  |  |  | chi2=0.12 | chi2=0.60 | chi2=0.00 | chi2=0.44 | chi2=0.13 |
|  |  |  | p=0.73133 (P) | p=0.43920 | p=0.96987 | p=0.50583 | p=0.71917 |
|  |  |  | **Risk allele 1** | | | | |
|  |  |  | **[2]<->[1]** | **[22]<->[12]** | **[22]<->[11]** | **[11+12]<->[22]** | **odds ratio** |
|  |  |  | OR=1.031 | OR=0.912 | OR=1.008 | OR=0.951 | OR=1.021 |
|  |  |  | C.I.=[0.868-1.224] | C.I.=[0.624-1.331] | C.I.=[0.682-1.488] | C.I.=[0.662-1.367] |  |
|  |  |  | chi2=0.12 | chi2=0.23 | chi2=0.00 | chi2=0.07 | chi2=0.13 |
|  |  |  | p=0.73133 (P) | p=0.63207 | p=0.96987 | p=0.78762 | p=0.71917 |
| rs2298553 | 0.426 | 0.214 | **Risk allele 2** | | | | |
|  |  |  |  |  |  |  |  |
|  |  |  |  |  |  |  |  |
|  |  |  | **[1]<->[2]** | **[11]<->[12]** | **[11+]<->[22]** | **[11]<->[12+22]** | **odds ratio** |
|  |  |  | OR=0.995 | OR=0.836 | OR=1.001 | OR=0.887 | OR=0.998 |
|  |  |  | C.I.=[0.843-1.175] | C.I.=[0.630-1.108] | C.I.=[0.720-1.391] | C.I.=[0.680-1.155] |  |
|  |  |  | chi2=0.00 | chi2=1.55 | chi2=0.00 | chi2=0.80 | chi2=0.00 |
|  |  |  | p=0.95552 (P) | p=0.21276 | p=0.99688 | p=0.37258 | p=0.95556 |
|  |  |  | **Risk allele 1** | | | | |
|  |  |  | **[2]<->[1]** | **[22]<->[12]** | **[22]<->[11]** | **[11+12]<->[22]** | **odds ratio** |
|  |  |  | OR=1.005 | OR=0.835 | OR=0.999 | OR=0.890 | OR=1.002 |
|  |  |  | C.I.=[0.851-1.187] | C.I.=[0.623-1.119] | C.I.=[0.719-1.389] | C.I.=[0.676-1.171] |  |
|  |  |  | chi2=0.00 | chi2=1.45 | chi2=0.00 | chi2=0.69 | chi2=0.00 |
|  |  |  | p=0.95552 (P) | p=0.22812 | p=0.99688 | p=0.40479 | p=0.95556 |
| rs569108 | 0.005 | 0.182 | **Risk allele 2** | | | | |
|  |  |  |  |  |  |  |  |
|  |  |  |  |  |  |  |  |
|  |  |  | **[1]<->[2]** | **[11]<->[12]** | **[11+]<->[22]** | **[11]<->[12+22]** | **odds ratio** |
|  |  |  | OR=0.719 | OR=0.774 | OR=0.432 | OR=0.741 | OR=0.736 |
|  |  |  | C.I.=[0.418-1.234] | C.I.=[0.431-1.388] | C.I.=[0.048-3.882] | C.I.=[0.421-1.304] |  |
|  |  |  | chi2=1.45 | chi2=0.74 | chi2=0.59 | chi2=1.09 | chi2=1.28 |
|  |  |  | p=0.22932 (P) | p=0.38863 | p=0.44092 | p=0.29757 | p=0.25818 |
|  |  |  | **Risk allele 1** | | | | |
|  |  |  | **[2]<->[1]** | **[22]<->[12]** | **[22]<->[11]** | **[11+12]<->[22]** | **odds ratio** |
|  |  |  | OR=1.392 | OR=1.789 | OR=2.313 | OR=2.286 | OR=1.373 |
|  |  |  | C.I.=[0.810-2.391] | C.I.=[0.186-17.230] | C.I.=[0.258-20.759] | C.I.=[0.255-20.522] |  |
|  |  |  | chi2=1.45 | chi2=0.26 | chi2=0.59 | chi2=0.58 | chi2=1.28 |
|  |  |  | p=0.22932 (P) | p=0.61030 | p=0.44092 | p=0.44745 | p=0.25818 |
| rs514524 | 0.810 | 0.912 | **Risk allele 2** | | | | |
|  |  |  |  |  |  |  |  |
|  |  |  |  |  |  |  |  |
|  |  |  | **[1]<->[2]** | **[11]<->[12]** | **[11+]<->[22]** | **[11]<->[12+22]** | **odds ratio** |
|  |  |  | OR=0.883 | OR=0.879 | OR=0.779 | OR=0.859 | OR=0.882 |
|  |  |  | C.I.=[0.739-1.055] | C.I.=[0.686-1.128] | C.I.=[0.519-1.171] | C.I.=[0.679-1.088] |  |
|  |  |  | chi2=1.89 | chi2=1.03 | chi2=1.44 | chi2=1.58 | chi2=1.91 |
|  |  |  | p=0.16906 (P) | p=0.31079 | p=0.22999 | p=0.20866 | p=0.16727 |
|  |  |  | **Risk allele 1** | | | | |
|  |  |  | **[2]<->[1]** | **[22]<->[12]** | **[22]<->[11]** | **[11+12]<->[22]** | **odds ratio** |
|  |  |  | OR=1.133 | OR=1.128 | OR=1.283 | OR=1.204 | OR=1.134 |
|  |  |  | C.I.=[0.948-1.354] | C.I.=[0.750-1.698] | C.I.=[0.854-1.928] | C.I.=[0.816-1.775] |  |
|  |  |  | chi2=1.89 | chi2=0.34 | chi2=1.44 | chi2=0.88 | chi2=1.91 |
|  |  |  | p=0.16906 (P) | p=0.56267 | p=0.22999 | p=0.34915 | p=0.16727 |
| rs4939426 | 0.019 | 1.000 | **Risk allele 2** | | | | |
|  |  |  |  |  |  |  |  |
|  |  |  |  |  |  |  |  |
|  |  |  | **[1]<->[2]** | **[11]<->[12]** | **[11+]<->[22]** | **[11]<->[12+22]** | **odds ratio** |
|  |  |  | OR=0.847 | OR=0.940 | OR=0.450 | OR=0.885 | OR=0.828 |
|  |  |  | C.I.=[0.651-1.102] | C.I.=[0.697-1.267] | C.I.=[0.167-1.216] | C.I.=[0.663-1.182] |  |
|  |  |  | chi2=1.53 | chi2=0.17 | chi2=2.61 | chi2=0.68 | chi2=1.44 |
|  |  |  | p=0.21656 (P) | p=0.68372 | p=0.10635 | p=0.40809 | p=0.23001 |
|  |  |  | **Risk allele 1** | | | | |
|  |  |  | **[2]<->[1]** | **[22]<->[12]** | **[22]<->[11]** | **[11+12]<->[22]** | **odds ratio** |
|  |  |  | OR=1.181 | OR=2.089 | OR=2.223 | OR=2.195 | OR=1.235 |
|  |  |  | C.I.=[0.907-1.536] | C.I.=[0.753-5.798] | C.I.=[0.823-6.005] | C.I.=[0.814-5.922] |  |
|  |  |  | chi2=1.53 | chi2=2.07 | chi2=2.61 | chi2=2.53 | chi2=1.44 |
|  |  |  | p=0.21656 (P) | p=0.14974 | p=0.10635 | p=0.11139 | p=0.23001 |
| rs1941030 | 0.878 | 0.407 | **Risk allele 2** | | | | |
|  |  |  |  |  |  |  |  |
|  |  |  |  |  |  |  |  |
|  |  |  | **[1]<->[2]** | **[11]<->[12]** | **[11+]<->[22]** | **[11]<->[12+22]** | **odds ratio** |
|  |  |  | OR=0.938 | OR=0.994 | OR=0.839 | OR=0.958 | OR=0.928 |
|  |  |  | C.I.=[0.790-1.115] | C.I.=[0.771-1.280] | C.I.=[0.574-1.225] | C.I.=[0.753-1.219] |  |
|  |  |  | chi2=0.53 | chi2=0.00 | chi2=0.83 | chi2=0.12 | chi2=0.54 |
|  |  |  | p=0.46779 (P) | p=0.96015 | p=0.36278 | p=0.72769 | p=0.46288 |
|  |  |  | **Risk allele 1** | | | | |
|  |  |  | **[2]<->[1]** | **[22]<->[12]** | **[22]<->[11]** | **[11+12]<->[22]** | **odds ratio** |
|  |  |  | OR=1.066 | OR=1.184 | OR=1.192 | OR=1.188 | OR=1.078 |
|  |  |  | C.I.=[0.897-1.266] | C.I.=[0.818-1.715] | C.I.=[0.816-1.741] | C.I.=[0.835-1.689] |  |
|  |  |  | chi2=0.53 | chi2=0.81 | chi2=0.83 | chi2=0.92 | chi2=0.54 |
|  |  |  | p=0.46779 (P) | p=0.36941 | p=0.36278 | p=0.33731 | p=0.46288 |
| rs4939353 | 0.128 | 0.545 | **Risk allele 2** | | | | |
|  |  |  |  |  |  |  |  |
|  |  |  |  |  |  |  |  |
|  |  |  | **[1]<->[2]** | **[11]<->[12]** | **[11+]<->[22]** | **[11]<->[12+22]** | **odds ratio** |
|  |  |  | OR=0.955 | OR=0.987 | OR=0.876 | OR=0.966 | OR=0.949 |
|  |  |  | C.I.=[0.793-1.150] | C.I.=[0.770-1.267] | C.I.=[0.565-1.356] | C.I.=[0.763-1.223] |  |
|  |  |  | chi2=0.24 | chi2=0.01 | chi2=0.35 | chi2=0.08 | chi2=0.23 |
|  |  |  | p=0.62617 (P) | p=0.92063 | p=0.55204 | p=0.77415 | p=0.63408 |
|  |  |  | **Risk allele 1** | | | | |
|  |  |  | **[2]<->[1]** | **[22]<->[12]** | **[22]<->[11]** | **[11+12]<->[22]** | **odds ratio** |
|  |  |  | OR=1.047 | OR=1.127 | OR=1.142 | OR=1.136 | OR=1.054 |
|  |  |  | C.I.=[0.869-1.262] | C.I.=[0.720-1.766] | C.I.=[0.737-1.768] | C.I.=[0.743-1.737] |  |
|  |  |  | chi2=0.24 | chi2=0.27 | chi2=0.35 | chi2=0.35 | chi2=0.23 |
|  |  |  | p=0.62617 (P) | p=0.60027 | p=0.55204 | p=0.55663 | p=0.63408 |
| rs3829247 | 0.785 | 0.806 | **Risk allele 2** | | | | |
|  |  |  |  |  |  |  |  |
|  |  |  |  |  |  |  |  |
|  |  |  | **[1]<->[2]** | **[11]<->[12]** | **[11+]<->[22]** | **[11]<->[12+22]** | **odds ratio** |
|  |  |  | OR=0.956 | OR=0.995 | OR=0.851 | OR=0.971 | OR=0.945 |
|  |  |  | C.I.=[0.792-1.153] | C.I.=[0.777-1.273] | C.I.=[0.530-1.365] | C.I.=[0.767-1.229] |  |
|  |  |  | chi2=0.23 | chi2=0.00 | chi2=0.45 | chi2=0.06 | chi2=0.23 |
|  |  |  | p=0.63502 (P) | p=0.96671 | p=0.50232 | p=0.80775 | p=0.63506 |
|  |  |  | **Risk allele 1** | | | | |
|  |  |  | **[2]<->[1]** | **[22]<->[12]** | **[22]<->[11]** | **[11+12]<->[22]** | **odds ratio** |
|  |  |  | OR=1.047 | OR=1.169 | OR=1.175 | OR=1.173 | OR=1.060 |
|  |  |  | C.I.=[0.867-1.263] | C.I.=[0.722-1.894] | C.I.=[0.733-1.886] | C.I.=[0.740-1.860] |  |
|  |  |  | chi2=0.23 | chi2=0.40 | chi2=0.45 | chi2=0.46 | chi2=0.23 |
|  |  |  | p=0.63502 (P) | p=0.52465 | p=0.50232 | p=0.49742 | p=0.63506 |
| rs708498 | 0.803 | 0.416 | **Risk allele 2** | | | | |
|  |  |  |  |  |  |  |  |
|  |  |  |  |  |  |  |  |
|  |  |  | **[1]<->[2]** | **[11]<->[12]** | **[11+]<->[22]** | **[11]<->[12+22]** | **odds ratio** |
|  |  |  | OR=1.029 | OR=1.072 | OR=0.893 | OR=1.056 | OR=1.013 |
|  |  |  | C.I.=[0.828-1.279] | C.I.=[0.829-1.386] | C.I.=[0.427-1.868] | C.I.=[0.822-1.356] |  |
|  |  |  | chi2=0.07 | chi2=0.28 | chi2=0.09 | chi2=0.18 | chi2=0.07 |
|  |  |  | p=0.79415 (P) | p=0.59768 | p=0.76385 | p=0.67030 | p=0.79135 |
|  |  |  | **Risk allele 1** | | | | |
|  |  |  | **[2]<->[1]** | **[22]<->[12]** | **[22]<->[11]** | **[11+12]<->[22]** | **odds ratio** |
|  |  |  | OR=0.972 | OR=1.200 | OR=1.120 | OR=1.144 | OR=0.989 |
|  |  |  | C.I.=[0.782-1.207] | C.I.=[0.564-2.552] | C.I.=[0.535-2.342] | C.I.=[0.549-2.382] |  |
|  |  |  | chi2=0.07 | chi2=0.22 | chi2=0.09 | chi2=0.13 | chi2=0.07 |
|  |  |  | p=0.79415 (P) | p=0.63543 | p=0.76385 | p=0.71904 | p=0.79135 |
| rs3818186 | 0.159 | 0.708 | **Risk allele 2** | | | | |
|  |  |  |  |  |  |  |  |
|  |  |  |  |  |  |  |  |
|  |  |  | **[1]<->[2]** | **[11]<->[12]** | **[11+]<->[22]** | **[11]<->[12+22]** | **odds ratio** |
|  |  |  | OR=0.992 | OR=0.897 | OR=1.199 | OR=0.934 | OR=1.025 |
|  |  |  | C.I.=[0.820-1.199] | C.I.=[0.701-1.149] | C.I.=[0.737-1.949] | C.I.=[0.738-1.184] |  |
|  |  |  | chi2=0.01 | chi2=0.74 | chi2=0.53 | chi2=0.32 | chi2=0.01 |
|  |  |  | p=0.93108 (P) | p=0.38919 | p=0.46491 | p=0.57406 | p=0.93012 |
|  |  |  | **Risk allele 1** | | | | |
|  |  |  | **[2]<->[1]** | **[22]<->[12]** | **[22]<->[11]** | **[11+12]<->[22]** | **odds ratio** |
|  |  |  | OR=1.008 | OR=0.748 | OR=0.834 | OR=0.797 | OR=0.978 |
|  |  |  | C.I.=[0.834-1.219] | C.I.=[0.455-1.230] | C.I.=[0.513-1.357] | C.I.=[0.496-1.283] |  |
|  |  |  | chi2=0.01 | chi2=1.31 | chi2=0.53 | chi2=0.88 | chi2=0.01 |
|  |  |  | p=0.93108 (P) | p=0.25212 | p=0.46491 | p=0.34948 | p=0.93012 |
| rs3759666 | 1.000 | 1.000 | **Risk allele 2** | | | | |
|  |  |  |  |  |  |  |  |
|  |  |  |  |  |  |  |  |
|  |  |  | **[1]<->[2]** | **[11]<->[12]** | **[11+]<->[22]** | **[11]<->[12+22]** | **odds ratio** |
|  |  |  | OR=1.073 | OR=1.074 | OR=1.757 | OR=1.074 | OR=1.074 |
|  |  |  | C.I.=[0.505-2.283] | C.I.=[0.503-2.296] | C.I.=[0.035-88.695] | C.I.=[0.503-2.296] |  |
|  |  |  | chi2=0.03 | chi2=0.03 | chi2=nan | chi2=0.03 | chi2=0.03 |
|  |  |  | p=0.85447 (P) | p=0.85358 | p=1.00000 | p=0.85358 | p=0.85358 |
|  |  |  | **Risk allele 1** | | | | |
|  |  |  | **[2]<->[1]** | **[22]<->[12]** | **[22]<->[11]** | **[11+12]<->[22]** | **odds ratio** |
|  |  |  | OR=0.932 | OR=0.622 | OR=0.569 | OR=0.570 | OR=0.887 |
|  |  |  | C.I.=[0.438-1.982] | C.I.=[0.012-33.549] | C.I.=[0.011-28.739] | C.I.=[0.011-28.788] |  |
|  |  |  | chi2=0.03 | chi2=nan | chi2=nan | chi2=nan | chi2=0.03 |
|  |  |  | p=0.85447 (P) | p=1.00000 | p=1.00000 | p=1.00000 | p=0.85358 |
| rs751026 | 0.828 | 0.056 | **Risk allele 2** | | | | |
|  |  |  |  |  |  |  |  |
|  |  |  |  |  |  |  |  |
|  |  |  | **[1]<->[2]** | **[11]<->[12]** | **[11+]<->[22]** | **[11]<->[12+22]** | **odds ratio** |
|  |  |  | OR=1.116 | OR=0.944 | OR=1.247 | OR=1.038 | OR=1.114 |
|  |  |  | C.I.=[0.945-1.318] | C.I.=[0.713-1.250] | C.I.=[0.902-1.724] | C.I.=[0.798-1.348] |  |
|  |  |  | chi2=1.68 | chi2=0.16 | chi2=1.79 | chi2=0.08 | chi2=1.61 |
|  |  |  | p=0.19545 (P) | p=0.68614 | p=0.18095 | p=0.78284 | p=0.20404 |
|  |  |  | **Risk allele 1** | | | | |
|  |  |  | **[2]<->[1]** | **[22]<->[12]** | **[22]<->[11]** | **[11+12]<->[22]** | **odds ratio** |
|  |  |  | OR=0.896 | OR=0.757 | OR=0.802 | OR=0.773 | OR=0.898 |
|  |  |  | C.I.=[0.759-1.058] | C.I.=[0.565-1.013] | C.I.=[0.580-1.108] | C.I.=[0.589-1.015] |  |
|  |  |  | chi2=1.68 | chi2=3.52 | chi2=1.79 | chi2=3.44 | chi2=1.61 |
|  |  |  | p=0.19545 (P) | p=0.06068 | p=0.18095 | p=0.06348 | p=0.20404 |
| rs2101919 | 0.185 | 0.701 | **Risk allele 2** | | | | |
|  |  |  |  |  |  |  |  |
|  |  |  |  |  |  |  |  |
|  |  |  | **[1]<->[2]** | **[11]<->[12]** | **[11+]<->[22]** | **[11]<->[12+22]** | **odds ratio** |
|  |  |  | OR=0.968 | OR=0.881 | OR=1.136 | OR=0.912 | OR=0.998 |
|  |  |  | C.I.=[0.799-1.172] | C.I.=[0.687-1.128] | C.I.=[0.691-1.866] | C.I.=[0.720-1.156] |  |
|  |  |  | chi2=0.11 | chi2=1.01 | chi2=0.25 | chi2=0.58 | chi2=0.12 |
|  |  |  | p=0.73673 (P) | p=0.31438 | p=0.61492 | p=0.44637 | p=0.73363 |
|  |  |  | **Risk allele 1** | | | | |
|  |  |  | **[2]<->[1]** | **[22]<->[12]** | **[22]<->[11]** | **[11+12]<->[22]** | **odds ratio** |
|  |  |  | OR=1.033 | OR=0.775 | OR=0.880 | OR=0.836 | OR=1.004 |
|  |  |  | C.I.=[0.853-1.251] | C.I.=[0.466-1.289] | C.I.=[0.536-1.447] | C.I.=[0.514-1.359] |  |
|  |  |  | chi2=0.11 | chi2=0.97 | chi2=0.25 | chi2=0.52 | chi2=0.12 |
|  |  |  | p=0.73673 (P) | p=0.32554 | p=0.61492 | p=0.46945 | p=0.73363 |
| rs17197 | 0.554 | 0.306 | **Risk allele 2** | | | | |
|  |  |  |  |  |  |  |  |
|  |  |  |  |  |  |  |  |
|  |  |  | **[1]<->[2]** | **[11]<->[12]** | **[11+]<->[22]** | **[11]<->[12+22]** | **odds ratio** |
|  |  |  | OR=0.956 | OR=0.994 | OR=0.670 | OR=0.974 | OR=0.937 |
|  |  |  | C.I.=[0.752-1.217] | C.I.=[0.757-1.305] | C.I.=[0.237-1.897] | C.I.=[0.745-1.272] |  |
|  |  |  | chi2=0.13 | chi2=0.00 | chi2=0.58 | chi2=0.04 | chi2=0.14 |
|  |  |  | p=0.71561 (P) | p=0.96695 | p=0.44789 | p=0.84433 | p=0.71029 |
|  |  |  | **Risk allele 1** | | | | |
|  |  |  | **[2]<->[1]** | **[22]<->[12]** | **[22]<->[11]** | **[11+12]<->[22]** | **odds ratio** |
|  |  |  | OR=1.046 | OR=1.484 | OR=1.492 | OR=1.490 | OR=1.073 |
|  |  |  | C.I.=[0.822-1.331] | C.I.=[0.515-4.274] | C.I.=[0.527-4.224] | C.I.=[0.528-4.208] |  |
|  |  |  | chi2=0.13 | chi2=0.54 | chi2=0.58 | chi2=0.57 | chi2=0.14 |
|  |  |  | p=0.71561 (P) | p=0.46223 | p=0.44789 | p=0.44853 | p=0.71029 |
| rs2273431 | 1.000 | 0.043 | **Risk allele 2** | | | | |
|  |  |  |  |  |  |  |  |
|  |  |  |  |  |  |  |  |
|  |  |  | **[1]<->[2]** | **[11]<->[12]** | **[11+]<->[22]** | **[11]<->[12+22]** | **odds ratio** |
|  |  |  | OR=1.182 | OR=1.041 | OR=2.859 | OR=1.117 | OR=1.261 |
|  |  |  | C.I.=[0.884-1.582] | C.I.=[0.750-1.444] | C.I.=[0.928-8.803] | C.I.=[0.814-1.532] |  |
|  |  |  | chi2=1.28 | chi2=0.06 | chi2=3.66 | chi2=0.47 | chi2=1.23 |
|  |  |  | p=0.25849 (P) | p=0.81025 | p=0.05585 | p=0.49343 | p=0.26832 |
|  |  |  | **Risk allele 1** | | | | |
|  |  |  | **[2]<->[1]** | **[22]<->[12]** | **[22]<->[11]** | **[11+12]<->[22]** | **odds ratio** |
|  |  |  | OR=0.846 | OR=0.364 | OR=0.350 | OR=0.352 | OR=0.824 |
|  |  |  | C.I.=[0.632-1.131] | C.I.=[0.114-1.158] | C.I.=[0.114-1.077] | C.I.=[0.114-1.083] |  |
|  |  |  | chi2=1.28 | chi2=3.13 | chi2=3.66 | chi2=3.62 | chi2=1.23 |
|  |  |  | p=0.25849 (P) | p=0.07668 | p=0.05585 | p=0.05711 | p=0.26832 |
| rs1307289 | 0.352 | 0.848 | **Risk allele 2** | | | | |
|  |  |  |  |  |  |  |  |
|  |  |  |  |  |  |  |  |
|  |  |  | **[1]<->[2]** | **[11]<->[12]** | **[11+]<->[22]** | **[11]<->[12+22]** | **odds ratio** |
|  |  |  | OR=0.885 | OR=0.912 | OR=0.708 | OR=0.891 | OR=0.879 |
|  |  |  | C.I.=[0.702-1.116] | C.I.=[0.695-1.197] | C.I.=[0.334-1.499] | C.I.=[0.685-1.158] |  |
|  |  |  | chi2=1.07 | chi2=0.44 | chi2=0.82 | chi2=0.75 | chi2=1.04 |
|  |  |  | p=0.30123 (P) | p=0.50858 | p=0.36418 | p=0.38643 | p=0.30786 |
|  |  |  | **Risk allele 1** | | | | |
|  |  |  | **[2]<->[1]** | **[22]<->[12]** | **[22]<->[11]** | **[11+12]<->[22]** | **odds ratio** |
|  |  |  | OR=1.130 | OR=1.290 | OR=1.413 | OR=1.380 | OR=1.141 |
|  |  |  | C.I.=[0.896-1.424] | C.I.=[0.595-2.796] | C.I.=[0.667-2.994] | C.I.=[0.654-2.913] |  |
|  |  |  | chi2=1.07 | chi2=0.42 | chi2=0.82 | chi2=0.72 | chi2=1.04 |
|  |  |  | p=0.30123 (P) | p=0.51868 | p=0.36418 | p=0.39660 | p=0.30786 |
| rs1138272 | 0.523 | 0.079 | **Risk allele 2** | | | | |
|  |  |  |  |  |  |  |  |
|  |  |  |  |  |  |  |  |
|  |  |  | **[1]<->[2]** | **[11]<->[12]** | **[11+]<->[22]** | **[11]<->[12+22]** | **odds ratio** |
|  |  |  | OR=0.781 | OR=0.726 | OR=1.047 | OR=0.745 | OR=0.821 |
|  |  |  | C.I.=[0.575-1.061] | C.I.=[0.516-1.021] | C.I.=[0.340-3.224] | C.I.=[0.535-1.036] |  |
|  |  |  | chi2=2.51 | chi2=3.41 | chi2=0.01 | chi2=3.08 | chi2=2.41 |
|  |  |  | p=0.11280 (P) | p=0.06465 | p=0.93592 | p=0.07911 | p=0.12033 |
|  |  |  | **Risk allele 1** | | | | |
|  |  |  | **[2]<->[1]** | **[22]<->[12]** | **[22]<->[11]** | **[11+12]<->[22]** | **odds ratio** |
|  |  |  | OR=1.280 | OR=0.693 | OR=0.955 | OR=0.911 | OR=1.219 |
|  |  |  | C.I.=[0.943-1.738] | C.I.=[0.217-2.213] | C.I.=[0.310-2.940] | C.I.=[0.296-2.802] |  |
|  |  |  | chi2=2.51 | chi2=0.39 | chi2=0.01 | chi2=0.03 | chi2=2.41 |
|  |  |  | p=0.11280 (P) | p=0.53393 | p=0.93592 | p=0.87074 | p=0.12033 |
| rs1254600 | 0.793 | 0.509 | **Risk allele 2** | | | | |
|  |  |  |  |  |  |  |  |
|  |  |  |  |  |  |  |  |
|  |  |  | **[1]<->[2]** | **[11]<->[12]** | **[11+]<->[22]** | **[11]<->[12+22]** | **odds ratio** |
|  |  |  | OR=1.074 | OR=1.138 | OR=0.908 | OR=1.117 | OR=1.049 |
|  |  |  | C.I.=[0.861-1.339] | C.I.=[0.877-1.478] | C.I.=[0.434-1.899] | C.I.=[0.867-1.438] |  |
|  |  |  | chi2=0.40 | chi2=0.95 | chi2=0.07 | chi2=0.73 | chi2=0.41 |
|  |  |  | p=0.52537 (P) | p=0.32945 | p=0.79791 | p=0.39282 | p=0.52375 |
|  |  |  | **Risk allele 1** | | | | |
|  |  |  | **[2]<->[1]** | **[22]<->[12]** | **[22]<->[11]** | **[11+12]<->[22]** | **odds ratio** |
|  |  |  | OR=0.931 | OR=1.254 | OR=1.101 | OR=1.144 | OR=0.955 |
|  |  |  | C.I.=[0.747-1.161] | C.I.=[0.589-2.670] | C.I.=[0.527-2.302] | C.I.=[0.549-2.382] |  |
|  |  |  | chi2=0.40 | chi2=0.34 | chi2=0.07 | chi2=0.13 | chi2=0.41 |
|  |  |  | p=0.52537 (P) | p=0.55726 | p=0.79791 | p=0.71904 | p=0.52375 |
| rs12419635 | 0.667 | 0.786 | **Risk allele 2** | | | | |
|  |  |  |  |  |  |  |  |
|  |  |  |  |  |  |  |  |
|  |  |  | **[1]<->[2]** | **[11]<->[12]** | **[11+]<->[22]** | **[11]<->[12+22]** | **odds ratio** |
|  |  |  | OR=1.078 | OR=1.088 | OR=1.069 | OR=1.087 | OR=1.076 |
|  |  |  | C.I.=[0.813-1.429] | C.I.=[0.800-1.479] | C.I.=[0.254-4.500] | C.I.=[0.804-1.472] |  |
|  |  |  | chi2=0.27 | chi2=0.29 | chi2=0.01 | chi2=0.30 | chi2=0.28 |
|  |  |  | p=0.60236 (P) | p=0.58969 | p=0.92736 | p=0.58690 | p=0.59745 |
|  |  |  | **Risk allele 1** | | | | |
|  |  |  | **[2]<->[1]** | **[22]<->[12]** | **[22]<->[11]** | **[11+12]<->[22]** | **odds ratio** |
|  |  |  | OR=0.928 | OR=1.018 | OR=0.935 | OR=0.950 | OR=0.929 |
|  |  |  | C.I.=[0.700-1.230] | C.I.=[0.237-4.374] | C.I.=[0.222-3.937] | C.I.=[0.226-3.993] |  |
|  |  |  | chi2=0.27 | chi2=0.00 | chi2=0.01 | chi2=0.00 | chi2=0.28 |
|  |  |  | p=0.60236 (P) | p=0.98107 | p=0.92736 | p=0.94369 | p=0.59745 |
| rs7941395 | 0.537 | 0.479 | **Risk allele 2** | | | | |
|  |  |  |  |  |  |  |  |
|  |  |  |  |  |  |  |  |
|  |  |  | **[1]<->[2]** | **[11]<->[12]** | **[11+]<->[22]** | **[11]<->[12+22]** | **odds ratio** |
|  |  |  | OR=1.041 | OR=0.950 | OR=1.148 | OR=0.993 | OR=1.054 |
|  |  |  | C.I.=[0.877-1.235] | C.I.=[0.736-1.227] | C.I.=[0.800-1.647] | C.I.=[0.780-1.264] |  |
|  |  |  | chi2=0.21 | chi2=0.16 | chi2=0.56 | chi2=0.00 | chi2=0.21 |
|  |  |  | p=0.64979 (P) | p=0.69350 | p=0.45505 | p=0.95439 | p=0.64936 |
|  |  |  | **Risk allele 1** | | | | |
|  |  |  | **[2]<->[1]** | **[22]<->[12]** | **[22]<->[11]** | **[11+12]<->[22]** | **odds ratio** |
|  |  |  | OR=0.961 | OR=0.828 | OR=0.871 | OR=0.847 | OR=0.949 |
|  |  |  | C.I.=[0.810-1.141] | C.I.=[0.581-1.178] | C.I.=[0.607-1.251] | C.I.=[0.607-1.183] |  |
|  |  |  | chi2=0.21 | chi2=1.10 | chi2=0.56 | chi2=0.95 | chi2=0.21 |
|  |  |  | p=0.64979 (P) | p=0.29388 | p=0.45505 | p=0.32975 | p=0.64936 |
| rs2277494 | 0.137 | 0.190 | **Risk allele 2** | | | | |
|  |  |  |  |  |  |  |  |
|  |  |  |  |  |  |  |  |
|  |  |  | **[1]<->[2]** | **[11]<->[12]** | **[11+]<->[22]** | **[11]<->[12+22]** | **odds ratio** |
|  |  |  | OR=0.995 | OR=0.986 | OR=1.008 | OR=0.990 | OR=0.998 |
|  |  |  | C.I.=[0.819-1.209] | C.I.=[0.766-1.269] | C.I.=[0.627-1.620] | C.I.=[0.779-1.257] |  |
|  |  |  | chi2=0.00 | chi2=0.01 | chi2=0.00 | chi2=0.01 | chi2=0.00 |
|  |  |  | p=0.96286 (P) | p=0.91317 | p=0.97398 | p=0.93197 | p=0.96390 |
|  |  |  | **Risk allele 1** | | | | |
|  |  |  | **[2]<->[1]** | **[22]<->[12]** | **[22]<->[11]** | **[11+12]<->[22]** | **odds ratio** |
|  |  |  | OR=1.005 | OR=0.978 | OR=0.992 | OR=0.987 | OR=1.002 |
|  |  |  | C.I.=[0.827-1.221] | C.I.=[0.598-1.600] | C.I.=[0.617-1.595] | C.I.=[0.620-1.572] |  |
|  |  |  | chi2=0.00 | chi2=0.01 | chi2=0.00 | chi2=0.00 | chi2=0.00 |
|  |  |  | p=0.96286 (P) | p=0.93033 | p=0.97398 | p=0.95608 | p=0.96390 |
| rs1051069 | 0.309 | 0.293 | **Risk allele 2** | | | | |
|  |  |  |  |  |  |  |  |
|  |  |  |  |  |  |  |  |
|  |  |  | **[1]<->[2]** | **[11]<->[12]** | **[11+]<->[22]** | **[11]<->[12+22]** | **odds ratio** |
|  |  |  | OR=0.951 | OR=0.974 | OR=0.903 | OR=0.949 | OR=0.949 |
|  |  |  | C.I.=[0.805-1.123] | C.I.=[0.720-1.317] | C.I.=[0.641-1.271] | C.I.=[0.713-1.264] |  |
|  |  |  | chi2=0.35 | chi2=0.03 | chi2=0.34 | chi2=0.13 | chi2=0.37 |
|  |  |  | p=0.55416 (P) | p=0.86377 | p=0.55699 | p=0.72174 | p=0.54519 |
|  |  |  | **Risk allele 1** | | | | |
|  |  |  | **[2]<->[1]** | **[22]<->[12]** | **[22]<->[11]** | **[11+12]<->[22]** | **odds ratio** |
|  |  |  | OR=1.052 | OR=1.079 | OR=1.108 | OR=1.087 | OR=1.053 |
|  |  |  | C.I.=[0.890-1.242] | C.I.=[0.814-1.430] | C.I.=[0.787-1.560] | C.I.=[0.832-1.421] |  |
|  |  |  | chi2=0.35 | chi2=0.28 | chi2=0.34 | chi2=0.38 | chi2=0.37 |
|  |  |  | p=0.55416 (P) | p=0.59577 | p=0.55699 | p=0.53879 | p=0.54519 |
| rs3763840 | 0.873 | 0.835 | **Risk allele 2** | | | | |
|  |  |  |  |  |  |  |  |
|  |  |  |  |  |  |  |  |
|  |  |  | **[1]<->[2]** | **[11]<->[12]** | **[11+]<->[22]** | **[11]<->[12+22]** | **odds ratio** |
|  |  |  | OR=0.961 | OR=0.956 | OR=0.921 | OR=0.939 | OR=0.961 |
|  |  |  | C.I.=[0.807-1.144] | C.I.=[0.657-1.391] | C.I.=[0.632-1.345] | C.I.=[0.659-1.339] |  |
|  |  |  | chi2=0.20 | chi2=0.06 | chi2=0.18 | chi2=0.12 | chi2=0.20 |
|  |  |  | p=0.65293 (P) | p=0.81343 | p=0.67140 | p=0.72830 | p=0.65418 |
|  |  |  | **Risk allele 1** | | | | |
|  |  |  | **[2]<->[1]** | **[22]<->[12]** | **[22]<->[11]** | **[11+12]<->[22]** | **odds ratio** |
|  |  |  | OR=1.041 | OR=1.037 | OR=1.085 | OR=1.048 | OR=1.041 |
|  |  |  | C.I.=[0.874-1.239] | C.I.=[0.806-1.334] | C.I.=[0.744-1.583] | C.I.=[0.825-1.329] |  |
|  |  |  | chi2=0.20 | chi2=0.08 | chi2=0.18 | chi2=0.15 | chi2=0.20 |
|  |  |  | p=0.65293 (P) | p=0.77551 | p=0.67140 | p=0.70254 | p=0.65418 |
| rs8013756 | 0.125 | 0.097 | **Risk allele 2** | | | | |
|  |  |  |  |  |  |  |  |
|  |  |  |  |  |  |  |  |
|  |  |  | **[1]<->[2]** | **[11]<->[12]** | **[11+]<->[22]** | **[11]<->[12+22]** | **odds ratio** |
|  |  |  | OR=1.068 | OR=4.987 | OR=4.545 | OR=4.636 | OR=1.206 |
|  |  |  | C.I.=[0.825-1.384] | C.I.=[1.122-22.170] | C.I.=[1.039-19.885] | C.I.=[1.061-20.256] |  |
|  |  |  | chi2=0.25 | chi2=5.39 | chi2=4.85 | chi2=5.02 | chi2=0.25 |
|  |  |  | p=0.61612 (P) | p=0.02024 | p=0.02765 | p=0.02512 | p=0.61747 |
|  |  |  | **Risk allele 1** | | | | |
|  |  |  | **[2]<->[1]** | **[22]<->[12]** | **[22]<->[11]** | **[11+12]<->[22]** | **odds ratio** |
|  |  |  | OR=0.936 | OR=1.097 | OR=0.220 | OR=1.015 | OR=0.901 |
|  |  |  | C.I.=[0.723-1.212] | C.I.=[0.823-1.463] | C.I.=[0.050-0.963] | C.I.=[0.765-1.346] |  |
|  |  |  | chi2=0.25 | chi2=0.40 | chi2=4.85 | chi2=0.01 | chi2=0.25 |
|  |  |  | p=0.61612 (P) | p=0.52671 | p=0.02765 | p=0.91876 | p=0.61747 |
| rs1032936 | 1.000 | 0.198 | **Risk allele 2** | | | | |
|  |  |  |  |  |  |  |  |
|  |  |  |  |  |  |  |  |
|  |  |  | **[1]<->[2]** | **[11]<->[12]** | **[11+]<->[22]** | **[11]<->[12+22]** | **odds ratio** |
|  |  |  | OR=0.872 | OR=0.794 | OR=0.861 | OR=0.807 | OR=0.894 |
|  |  |  | C.I.=[0.726-1.046] | C.I.=[0.619-1.019] | C.I.=[0.568-1.304] | C.I.=[0.637-1.021] |  |
|  |  |  | chi2=2.18 | chi2=3.28 | chi2=0.50 | chi2=3.20 | chi2=2.14 |
|  |  |  | p=0.13963 (P) | p=0.07021 | p=0.47891 | p=0.07365 | p=0.14356 |
|  |  |  | **Risk allele 1** | | | | |
|  |  |  | **[2]<->[1]** | **[22]<->[12]** | **[22]<->[11]** | **[11+12]<->[22]** | **odds ratio** |
|  |  |  | OR=1.147 | OR=0.923 | OR=1.162 | OR=1.047 | OR=1.117 |
|  |  |  | C.I.=[0.956-1.377] | C.I.=[0.604-1.410] | C.I.=[0.767-1.760] | C.I.=[0.702-1.563] |  |
|  |  |  | chi2=2.18 | chi2=0.14 | chi2=0.50 | chi2=0.05 | chi2=2.14 |
|  |  |  | p=0.13963 (P) | p=0.71057 | p=0.47891 | p=0.82135 | p=0.14356 |
| rs1254601 | 0.418 | 0.376 | **Risk allele 2** | | | | |
|  |  |  |  |  |  |  |  |
|  |  |  |  |  |  |  |  |
|  |  |  | **[1]<->[2]** | **[11]<->[12]** | **[11+]<->[22]** | **[11]<->[12+22]** | **odds ratio** |
|  |  |  | OR=0.946 | OR=0.832 | OR=0.937 | OR=0.859 | OR=0.956 |
|  |  |  | C.I.=[0.800-1.120] | C.I.=[0.638-1.084] | C.I.=[0.666-1.319] | C.I.=[0.670-1.102] |  |
|  |  |  | chi2=0.41 | chi2=1.86 | chi2=0.14 | chi2=1.42 | chi2=0.41 |
|  |  |  | p=0.52170 (P) | p=0.17215 | p=0.71080 | p=0.23284 | p=0.52090 |
|  |  |  | **Risk allele 1** | | | | |
|  |  |  | **[2]<->[1]** | **[22]<->[12]** | **[22]<->[11]** | **[11+12]<->[22]** | **odds ratio** |
|  |  |  | OR=1.057 | OR=0.887 | OR=1.067 | OR=0.956 | OR=1.045 |
|  |  |  | C.I.=[0.893-1.250] | C.I.=[0.642-1.225] | C.I.=[0.758-1.501] | C.I.=[0.705-1.296] |  |
|  |  |  | chi2=0.41 | chi2=0.53 | chi2=0.14 | chi2=0.08 | chi2=0.41 |
|  |  |  | p=0.52170 (P) | p=0.46753 | p=0.71080 | p=0.77223 | p=0.52090 |
| rs7145029 | 0.896 | 0.742 | **Risk allele 2** | | | | |
|  |  |  |  |  |  |  |  |
|  |  |  |  |  |  |  |  |
|  |  |  | **[1]<->[2]** | **[11]<->[12]** | **[11+]<->[22]** | **[11]<->[12+22]** | **odds ratio** |
|  |  |  | OR=1.088 | OR=1.124 | OR=1.038 | OR=1.117 | OR=1.073 |
|  |  |  | C.I.=[0.873-1.356] | C.I.=[0.866-1.460] | C.I.=[0.503-2.140] | C.I.=[0.867-1.438] |  |
|  |  |  | chi2=0.56 | chi2=0.78 | chi2=0.01 | chi2=0.73 | chi2=0.56 |
|  |  |  | p=0.45427 (P) | p=0.37845 | p=0.91971 | p=0.39282 | p=0.45251 |
|  |  |  | **Risk allele 1** | | | | |
|  |  |  | **[2]<->[1]** | **[22]<->[12]** | **[22]<->[11]** | **[11+12]<->[22]** | **odds ratio** |
|  |  |  | OR=0.919 | OR=1.083 | OR=0.963 | OR=0.997 | OR=0.931 |
|  |  |  | C.I.=[0.738-1.146] | C.I.=[0.516-2.276] | C.I.=[0.467-1.986] | C.I.=[0.486-2.047] |  |
|  |  |  | chi2=0.56 | chi2=0.04 | chi2=0.01 | chi2=0.00 | chi2=0.56 |
|  |  |  | p=0.45427 (P) | p=0.83257 | p=0.91971 | p=0.99415 | p=0.45251 |
| rs10498475 | 1.000 | 1.000 | **Risk allele 2** | | | | |
|  |  |  |  |  |  |  |  |
|  |  |  |  |  |  |  |  |
|  |  |  | **[1]<->[2]** | **[11]<->[12]** | **[11+]<->[22]** | **[11]<->[12+22]** | **odds ratio** |
|  |  |  | OR=0.922 | OR=0.938 | OR=0.579 | OR=0.928 | OR=0.913 |
|  |  |  | C.I.=[0.659-1.289] | C.I.=[0.658-1.337] | C.I.=[0.060-5.588] | C.I.=[0.653-1.318] |  |
|  |  |  | chi2=0.23 | chi2=0.12 | chi2=0.23 | chi2=0.18 | chi2=0.23 |
|  |  |  | p=0.63437 (P) | p=0.72434 | p=0.63259 | p=0.67539 | p=0.63104 |
|  |  |  | **Risk allele 1** | | | | |
|  |  |  | **[2]<->[1]** | **[22]<->[12]** | **[22]<->[11]** | **[11+12]<->[22]** | **odds ratio** |
|  |  |  | OR=1.085 | OR=1.620 | OR=1.727 | OR=1.713 | OR=1.101 |
|  |  |  | C.I.=[0.776-1.516] | C.I.=[0.165-15.953] | C.I.=[0.179-16.657] | C.I.=[0.178-16.515] |  |
|  |  |  | chi2=0.23 | chi2=0.17 | chi2=0.23 | chi2=0.22 | chi2=0.23 |
|  |  |  | p=0.63437 (P) | p=0.67654 | p=0.63259 | p=0.63772 | p=0.63104 |
| rs17126074 | 1.000 | 1.000 | **Risk allele 2** | | | | |
|  |  |  |  |  |  |  |  |
|  |  |  |  |  |  |  |  |
|  |  |  | **[1]<->[2]** | **[11]<->[12]** | **[11+]<->[22]** | **[11]<->[12+22]** | **odds ratio** |
|  |  |  | OR=0.546 | OR=0.543 | OR=1.737 | OR=0.543 | OR=0.543 |
|  |  |  | C.I.=[0.199-1.495] | C.I.=[0.198-1.493] | C.I.=[0.034-87.694] | C.I.=[0.198-1.493] |  |
|  |  |  | chi2=1.43 | chi2=1.44 | chi2=nan | chi2=1.44 | chi2=1.44 |
|  |  |  | p=0.23176 (P) | p=0.22970 | p=1.00000 | p=0.22970 | p=0.22970 |
|  |  |  | **Risk allele 1** | | | | |
|  |  |  | **[2]<->[1]** | **[22]<->[12]** | **[22]<->[11]** | **[11+12]<->[22]** | **odds ratio** |
|  |  |  | OR=1.832 | OR=0.333 | OR=0.576 | OR=0.570 | OR=1.426 |
|  |  |  | C.I.=[0.669-5.019] | C.I.=[0.006-18.885] | C.I.=[0.011-29.066] | C.I.=[0.011-28.788] |  |
|  |  |  | chi2=1.43 | chi2=nan | chi2=nan | chi2=nan | chi2=1.44 |
|  |  |  | p=0.23176 (P) | p=1.00000 | p=1.00000 | p=1.00000 | p=0.22970 |
| rs8004624 | 0.772 | 0.152 | **Risk allele 2** | | | | |
|  |  |  |  |  |  |  |  |
|  |  |  |  |  |  |  |  |
|  |  |  | **[1]<->[2]** | **[11]<->[12]** | **[11+]<->[22]** | **[11]<->[12+22]** | **odds ratio** |
|  |  |  | OR=1.094 | OR=0.972 | OR=1.188 | OR=1.043 | OR=1.090 |
|  |  |  | C.I.=[0.926-1.292] | C.I.=[0.729-1.297] | C.I.=[0.858-1.644] | C.I.=[0.797-1.366] |  |
|  |  |  | chi2=1.12 | chi2=0.04 | chi2=1.07 | chi2=0.10 | chi2=1.08 |
|  |  |  | p=0.29064 (P) | p=0.84921 | p=0.29983 | p=0.75654 | p=0.29835 |
|  |  |  | **Risk allele 1** | | | | |
|  |  |  | **[2]<->[1]** | **[22]<->[12]** | **[22]<->[11]** | **[11+12]<->[22]** | **odds ratio** |
|  |  |  | OR=0.914 | OR=0.819 | OR=0.842 | OR=0.827 | OR=0.917 |
|  |  |  | C.I.=[0.774-1.080] | C.I.=[0.616-1.089] | C.I.=[0.608-1.166] | C.I.=[0.633-1.079] |  |
|  |  |  | chi2=1.12 | chi2=1.89 | chi2=1.07 | chi2=1.96 | chi2=1.08 |
|  |  |  | p=0.29064 (P) | p=0.16883 | p=0.29983 | p=0.16157 | p=0.29835 |
| rs6572868 | 0.556 | 0.439 | **Risk allele 2** | | | | |
|  |  |  |  |  |  |  |  |
|  |  |  |  |  |  |  |  |
|  |  |  | **[1]<->[2]** | **[11]<->[12]** | **[11+]<->[22]** | **[11]<->[12+22]** | **odds ratio** |
|  |  |  | OR=0.999 | OR=0.930 | OR=1.741 | OR=0.962 | OR=1.042 |
|  |  |  | C.I.=[0.760-1.314] | C.I.=[0.686-1.262] | C.I.=[0.557-5.441] | C.I.=[0.715-1.295] |  |
|  |  |  | chi2=0.00 | chi2=0.22 | chi2=0.93 | chi2=0.06 | chi2=0.00 |
|  |  |  | p=0.99648 (P) | p=0.64229 | p=0.33391 | p=0.80027 | p=0.99647 |
|  |  |  | **Risk allele 1** | | | | |
|  |  |  | **[2]<->[1]** | **[22]<->[12]** | **[22]<->[11]** | **[11+12]<->[22]** | **odds ratio** |
|  |  |  | OR=1.001 | OR=0.534 | OR=0.574 | OR=0.567 | OR=0.973 |
|  |  |  | C.I.=[0.761-1.315] | C.I.=[0.167-1.712] | C.I.=[0.184-1.794] | C.I.=[0.182-1.767] |  |
|  |  |  | chi2=0.00 | chi2=1.14 | chi2=0.93 | chi2=0.98 | chi2=0.00 |
|  |  |  | p=0.99648 (P) | p=0.28467 | p=0.33391 | p=0.32136 | p=0.99647 |
| rs3825596 | 0.612 | 1.000 | **Risk allele 2** | | | | |
|  |  |  |  |  |  |  |  |
|  |  |  |  |  |  |  |  |
|  |  |  | **[1]<->[2]** | **[11]<->[12]** | **[11+]<->[22]** | **[11]<->[12+22]** | **odds ratio** |
|  |  |  | OR=1.100 | OR=1.070 | OR=1.778 | OR=1.088 | OR=1.122 |
|  |  |  | C.I.=[0.812-1.488] | C.I.=[0.772-1.484] | C.I.=[0.357-8.855] | C.I.=[0.789-1.502] |  |
|  |  |  | chi2=0.38 | chi2=0.16 | chi2=0.51 | chi2=0.27 | chi2=0.38 |
|  |  |  | p=0.53906 (P) | p=0.68507 | p=0.47635 | p=0.60638 | p=0.53500 |
|  |  |  | **Risk allele 1** | | | | |
|  |  |  | **[2]<->[1]** | **[22]<->[12]** | **[22]<->[11]** | **[11+12]<->[22]** | **odds ratio** |
|  |  |  | OR=0.909 | OR=0.602 | OR=0.562 | OR=0.568 | OR=0.897 |
|  |  |  | C.I.=[0.672-1.231] | C.I.=[0.118-3.066] | C.I.=[0.113-2.801] | C.I.=[0.114-2.828] |  |
|  |  |  | chi2=0.38 | chi2=0.38 | chi2=0.51 | chi2=0.49 | chi2=0.38 |
|  |  |  | p=0.53906 (P) | p=0.53705 | p=0.47635 | p=0.48427 | p=0.53500 |
| rs17127622 | 1.000 | 0.174 | **Risk allele 2** | | | | |
|  |  |  |  |  |  |  |  |
|  |  |  |  |  |  |  |  |
|  |  |  | **[1]<->[2]** | **[11]<->[12]** | **[11+]<->[22]** | **[11]<->[12+22]** | **odds ratio** |
|  |  |  | OR=0.669 | OR=0.622 | OR=1.119 | OR=0.638 | OR=0.706 |
|  |  |  | C.I.=[0.454-0.988] | C.I.=[0.410-0.945] | C.I.=[0.186-6.725] | C.I.=[0.424-0.961] |  |
|  |  |  | chi2=4.13 | chi2=5.02 | chi2=0.02 | chi2=4.70 | chi2=4.04 |
|  |  |  | p=0.04203 (P) | p=0.02512 | p=0.90226 | p=0.03024 | p=0.04431 |
|  |  |  | **Risk allele 1** | | | | |
|  |  |  | **[2]<->[1]** | **[22]<->[12]** | **[22]<->[11]** | **[11+12]<->[22]** | **odds ratio** |
|  |  |  | OR=1.494 | OR=0.556 | OR=0.894 | OR=0.854 | OR=1.421 |
|  |  |  | C.I.=[1.012-2.205] | C.I.=[0.089-3.478] | C.I.=[0.149-5.372] | C.I.=[0.142-5.133] |  |
|  |  |  | chi2=4.13 | chi2=0.40 | chi2=0.02 | chi2=0.03 | chi2=4.04 |
|  |  |  | p=0.04203 (P) | p=0.52527 | p=0.90226 | p=0.86323 | p=0.04431 |
| rs762063 | 0.607 | 0.162 | **Risk allele 2** | | | | |
|  |  |  |  |  |  |  |  |
|  |  |  |  |  |  |  |  |
|  |  |  | **[1]<->[2]** | **[11]<->[12]** | **[11+]<->[22]** | **[11]<->[12+22]** | **odds ratio** |
|  |  |  | OR=0.864 | OR=1.004 | OR=0.693 | OR=0.915 | OR=0.849 |
|  |  |  | C.I.=[0.729-1.023] | C.I.=[0.772-1.304] | C.I.=[0.484-0.992] | C.I.=[0.713-1.173] |  |
|  |  |  | chi2=2.88 | chi2=0.00 | chi2=4.04 | chi2=0.49 | chi2=2.92 |
|  |  |  | p=0.08970 (P) | p=0.97826 | p=0.04456 | p=0.48212 | p=0.08773 |
|  |  |  | **Risk allele 1** | | | | |
|  |  |  | **[2]<->[1]** | **[22]<->[12]** | **[22]<->[11]** | **[11+12]<->[22]** | **odds ratio** |
|  |  |  | OR=1.158 | OR=1.449 | OR=1.444 | OR=1.447 | OR=1.180 |
|  |  |  | C.I.=[0.978-1.371] | C.I.=[1.032-2.036] | C.I.=[1.008-2.068] | C.I.=[1.047-1.999] |  |
|  |  |  | chi2=2.88 | chi2=4.60 | chi2=4.04 | chi2=5.06 | chi2=2.92 |
|  |  |  | p=0.08970 (P) | p=0.03191 | p=0.04456 | p=0.02451 | p=0.08773 |
| rs12889199 | 0.796 | 0.456 | **Risk allele 2** | | | | |
|  |  |  |  |  |  |  |  |
|  |  |  |  |  |  |  |  |
|  |  |  | **[1]<->[2]** | **[11]<->[12]** | **[11+]<->[22]** | **[11]<->[12+22]** | **odds ratio** |
|  |  |  | OR=0.908 | OR=0.888 | OR=1.037 | OR=0.895 | OR=0.921 |
|  |  |  | C.I.=[0.659-1.253] | C.I.=[0.626-1.259] | C.I.=[0.246-4.361] | C.I.=[0.636-1.259] |  |
|  |  |  | chi2=0.34 | chi2=0.45 | chi2=0.00 | chi2=0.41 | chi2=0.34 |
|  |  |  | p=0.55767 (P) | p=0.50443 | p=0.96099 | p=0.52247 | p=0.56099 |
|  |  |  | **Risk allele 1** | | | | |
|  |  |  | **[2]<->[1]** | **[22]<->[12]** | **[22]<->[11]** | **[11+12]<->[22]** | **odds ratio** |
|  |  |  | OR=1.101 | OR=0.857 | OR=0.965 | OR=0.950 | OR=1.086 |
|  |  |  | C.I.=[0.798-1.518] | C.I.=[0.197-3.718] | C.I.=[0.229-4.060] | C.I.=[0.226-3.993] |  |
|  |  |  | chi2=0.34 | chi2=0.04 | chi2=0.00 | chi2=0.00 | chi2=0.34 |
|  |  |  | p=0.55767 (P) | p=0.83624 | p=0.96099 | p=0.94369 | p=0.56099 |
| rs17253619 | 0.453 | 0.491 | **Risk allele 2** | | | | |
|  |  |  |  |  |  |  |  |
|  |  |  |  |  |  |  |  |
|  |  |  | **[1]<->[2]** | **[11]<->[12]** | **[11+]<->[22]** | **[11]<->[12+22]** | **odds ratio** |
|  |  |  | OR=1.113 | OR=1.210 | OR=0.660 | OR=1.171 | OR=1.076 |
|  |  |  | C.I.=[0.857-1.444] | C.I.=[0.905-1.618] | C.I.=[0.209-2.089] | C.I.=[0.881-1.557] |  |
|  |  |  | chi2=0.65 | chi2=1.66 | chi2=0.51 | chi2=1.19 | chi2=0.65 |
|  |  |  | p=0.42167 (P) | p=0.19751 | p=0.47688 | p=0.27580 | p=0.42133 |
|  |  |  | **Risk allele 1** | | | | |
|  |  |  | **[2]<->[1]** | **[22]<->[12]** | **[22]<->[11]** | **[11+12]<->[22]** | **odds ratio** |
|  |  |  | OR=0.899 | OR=1.833 | OR=1.515 | OR=1.576 | OR=0.941 |
|  |  |  | C.I.=[0.693-1.166] | C.I.=[0.567-5.926] | C.I.=[0.479-4.794] | C.I.=[0.499-4.978] |  |
|  |  |  | chi2=0.65 | chi2=1.05 | chi2=0.51 | chi2=0.61 | chi2=0.65 |
|  |  |  | p=0.42167 (P) | p=0.30484 | p=0.47688 | p=0.43479 | p=0.42133 |
| rs2357947 | 1.000 | 0.612 | **Risk allele 2** | | | | |
|  |  |  |  |  |  |  |  |
|  |  |  |  |  |  |  |  |
|  |  |  | **[1]<->[2]** | **[11]<->[12]** | **[11+]<->[22]** | **[11]<->[12+22]** | **odds ratio** |
|  |  |  | OR=1.067 | OR=1.025 | OR=1.518 | OR=1.048 | OR=1.090 |
|  |  |  | C.I.=[0.813-1.400] | C.I.=[0.756-1.388] | C.I.=[0.506-4.552] | C.I.=[0.780-1.409] |  |
|  |  |  | chi2=0.22 | chi2=0.02 | chi2=0.56 | chi2=0.10 | chi2=0.22 |
|  |  |  | p=0.64166 (P) | p=0.87568 | p=0.45336 | p=0.75603 | p=0.64211 |
|  |  |  | **Risk allele 1** | | | | |
|  |  |  | **[2]<->[1]** | **[22]<->[12]** | **[22]<->[11]** | **[11+12]<->[22]** | **odds ratio** |
|  |  |  | OR=0.937 | OR=0.675 | OR=0.659 | OR=0.662 | OR=0.923 |
|  |  |  | C.I.=[0.714-1.230] | C.I.=[0.219-2.078] | C.I.=[0.220-1.976] | C.I.=[0.221-1.982] |  |
|  |  |  | chi2=0.22 | chi2=0.47 | chi2=0.56 | chi2=0.55 | chi2=0.22 |
|  |  |  | p=0.64166 (P) | p=0.49086 | p=0.45336 | p=0.45770 | p=0.64211 |
| rs11622740 | 0.813 | 0.232 | **Risk allele 2** | | | | |
|  |  |  |  |  |  |  |  |
|  |  |  |  |  |  |  |  |
|  |  |  | **[1]<->[2]** | **[11]<->[12]** | **[11+]<->[22]** | **[11]<->[12+22]** | **odds ratio** |
|  |  |  | OR=0.884 | OR=0.819 | OR=0.843 | OR=0.824 | OR=0.900 |
|  |  |  | C.I.=[0.742-1.054] | C.I.=[0.637-1.053] | C.I.=[0.578-1.229] | C.I.=[0.650-1.044] |  |
|  |  |  | chi2=1.89 | chi2=2.43 | chi2=0.79 | chi2=2.56 | chi2=1.84 |
|  |  |  | p=0.16965 (P) | p=0.11936 | p=0.37400 | p=0.10927 | p=0.17553 |
|  |  |  | **Risk allele 1** | | | | |
|  |  |  | **[2]<->[1]** | **[22]<->[12]** | **[22]<->[11]** | **[11+12]<->[22]** | **odds ratio** |
|  |  |  | OR=1.131 | OR=0.972 | OR=1.187 | OR=1.074 | OR=1.110 |
|  |  |  | C.I.=[0.949-1.348] | C.I.=[0.665-1.419] | C.I.=[0.814-1.730] | C.I.=[0.752-1.534] |  |
|  |  |  | chi2=1.89 | chi2=0.02 | chi2=0.79 | chi2=0.15 | chi2=1.84 |
|  |  |  | p=0.16965 (P) | p=0.88125 | p=0.37400 | p=0.69390 | p=0.17553 |
| rs946615 | 0.305 | 0.434 | **Risk allele 2** | | | | |
|  |  |  |  |  |  |  |  |
|  |  |  |  |  |  |  |  |
|  |  |  | **[1]<->[2]** | **[11]<->[12]** | **[11+]<->[22]** | **[11]<->[12+22]** | **odds ratio** |
|  |  |  | OR=0.901 | OR=0.810 | OR=1.000 | OR=0.835 | OR=0.932 |
|  |  |  | C.I.=[0.744-1.092] | C.I.=[0.632-1.039] | C.I.=[0.613-1.630] | C.I.=[0.659-1.059] |  |
|  |  |  | chi2=1.12 | chi2=2.75 | chi2=0.00 | chi2=2.21 | chi2=1.14 |
|  |  |  | p=0.28898 (P) | p=0.09744 | p=0.99973 | p=0.13741 | p=0.28636 |
|  |  |  | **Risk allele 1** | | | | |
|  |  |  | **[2]<->[1]** | **[22]<->[12]** | **[22]<->[11]** | **[11+12]<->[22]** | **odds ratio** |
|  |  |  | OR=1.109 | OR=0.810 | OR=1.000 | OR=0.919 | OR=1.073 |
|  |  |  | C.I.=[0.916-1.344] | C.I.=[0.491-1.338] | C.I.=[0.614-1.630] | C.I.=[0.569-1.482] |  |
|  |  |  | chi2=1.12 | chi2=0.68 | chi2=0.00 | chi2=0.12 | chi2=1.14 |
|  |  |  | p=0.28898 (P) | p=0.41068 | p=0.99973 | p=0.72827 | p=0.28636 |
| rs2277495 | 0.359 | 0.487 | **Risk allele 2** | | | | |
|  |  |  |  |  |  |  |  |
|  |  |  |  |  |  |  |  |
|  |  |  | **[1]<->[2]** | **[11]<->[12]** | **[11+]<->[22]** | **[11]<->[12+22]** | **odds ratio** |
|  |  |  | OR=0.888 | OR=0.889 | OR=0.798 | OR=0.870 | OR=0.892 |
|  |  |  | C.I.=[0.741-1.065] | C.I.=[0.693-1.140] | C.I.=[0.528-1.206] | C.I.=[0.688-1.101] |  |
|  |  |  | chi2=1.64 | chi2=0.86 | chi2=1.15 | chi2=1.34 | chi2=1.58 |
|  |  |  | p=0.20057 (P) | p=0.35465 | p=0.28289 | p=0.24779 | p=0.20808 |
|  |  |  | **Risk allele 1** | | | | |
|  |  |  | **[2]<->[1]** | **[22]<->[12]** | **[22]<->[11]** | **[11+12]<->[22]** | **odds ratio** |
|  |  |  | OR=1.126 | OR=1.115 | OR=1.254 | OR=1.189 | OR=1.121 |
|  |  |  | C.I.=[0.939-1.350] | C.I.=[0.732-1.698] | C.I.=[0.829-1.895] | C.I.=[0.799-1.769] |  |
|  |  |  | chi2=1.64 | chi2=0.26 | chi2=1.15 | chi2=0.73 | chi2=1.58 |
|  |  |  | p=0.20057 (P) | p=0.61285 | p=0.28289 | p=0.39417 | p=0.20808 |
| rs3751464 | 0.909 | 0.901 | **Risk allele 2** | | | | |
|  |  |  |  |  |  |  |  |
|  |  |  |  |  |  |  |  |
|  |  |  | **[1]<->[2]** | **[11]<->[12]** | **[11+]<->[22]** | **[11]<->[12+22]** | **odds ratio** |
|  |  |  | OR=1.434 | OR=1.427 | OR=2.062 | OR=1.497 | OR=1.433 |
|  |  |  | C.I.=[1.178-1.747] | C.I.=[1.110-1.833] | C.I.=[1.215-3.501] | C.I.=[1.178-1.903] |  |
|  |  |  | chi2=12.93 | chi2=7.74 | chi2=7.44 | chi2=10.91 | chi2=12.78 |
|  |  |  | p=0.00032 (P) | p=0.00542 | p=0.00637 | p=0.00095 | p=0.00035 |
|  |  |  | **Risk allele 1** | | | | |
|  |  |  | **[2]<->[1]** | **[22]<->[12]** | **[22]<->[11]** | **[11+12]<->[22]** | **odds ratio** |
|  |  |  | OR=0.697 | OR=0.692 | OR=0.485 | OR=0.552 | OR=0.698 |
|  |  |  | C.I.=[0.572-0.849] | C.I.=[0.402-1.191] | C.I.=[0.286-0.823] | C.I.=[0.328-0.929] |  |
|  |  |  | chi2=12.93 | chi2=1.78 | chi2=7.44 | chi2=5.12 | chi2=12.78 |
|  |  |  | p=0.00032 (P) | p=0.18196 | p=0.00637 | p=0.02360 | p=0.00035 |
| rs17127595 | 0.645 | 0.706 | **Risk allele 2** | | | | |
|  |  |  |  |  |  |  |  |
|  |  |  |  |  |  |  |  |
|  |  |  | **[1]<->[2]** | **[11]<->[12]** | **[11+]<->[22]** | **[11]<->[12+22]** | **odds ratio** |
|  |  |  | OR=1.117 | OR=1.059 | OR=1.648 | OR=1.095 | OR=1.150 |
|  |  |  | C.I.=[0.882-1.415] | C.I.=[0.805-1.392] | C.I.=[0.719-3.777] | C.I.=[0.840-1.428] |  |
|  |  |  | chi2=0.85 | chi2=0.17 | chi2=1.42 | chi2=0.45 | chi2=0.86 |
|  |  |  | p=0.35685 (P) | p=0.68120 | p=0.23361 | p=0.50305 | p=0.35509 |
|  |  |  | **Risk allele 1** | | | | |
|  |  |  | **[2]<->[1]** | **[22]<->[12]** | **[22]<->[11]** | **[11+12]<->[22]** | **odds ratio** |
|  |  |  | OR=0.895 | OR=0.643 | OR=0.607 | OR=0.616 | OR=0.876 |
|  |  |  | C.I.=[0.707-1.133] | C.I.=[0.274-1.506] | C.I.=[0.265-1.391] | C.I.=[0.269-1.407] |  |
|  |  |  | chi2=0.85 | chi2=1.05 | chi2=1.42 | chi2=1.35 | chi2=0.86 |
|  |  |  | p=0.35685 (P) | p=0.30594 | p=0.23361 | p=0.24590 | p=0.35509 |
| rs1695 | 0.552 | 0.739 | **Risk allele 2** | | | | |
|  |  |  |  |  |  |  |  |
|  |  |  |  |  |  |  |  |
|  |  |  | **[1]<->[2]** | **[11]<->[12]** | **[11+]<->[22]** | **[11]<->[12+22]** | **odds ratio** |
|  |  |  | OR=1.033 | OR=1.103 | OR=0.986 | OR=1.081 | OR=1.018 |
|  |  |  | C.I.=[0.864-1.237] | C.I.=[0.861-1.414] | C.I.=[0.650-1.495] | C.I.=[0.854-1.368] |  |
|  |  |  | chi2=0.13 | chi2=0.61 | chi2=0.00 | chi2=0.42 | chi2=0.13 |
|  |  |  | p=0.72008 (P) | p=0.43601 | p=0.94686 | p=0.51811 | p=0.72100 |
|  |  |  | **Risk allele 1** | | | | |
|  |  |  | **[2]<->[1]** | **[22]<->[12]** | **[22]<->[11]** | **[11+12]<->[22]** | **odds ratio** |
|  |  |  | OR=0.968 | OR=1.119 | OR=1.014 | OR=1.063 | OR=0.983 |
|  |  |  | C.I.=[0.809-1.158] | C.I.=[0.735-1.703] | C.I.=[0.669-1.538] | C.I.=[0.713-1.584] |  |
|  |  |  | chi2=0.13 | chi2=0.28 | chi2=0.00 | chi2=0.09 | chi2=0.13 |
|  |  |  | p=0.72008 (P) | p=0.59906 | p=0.94686 | p=0.76539 | p=0.72100 |
| rs11431 | 0.043 | 0.069 | **Risk allele 2** | | | | |
|  |  |  |  |  |  |  |  |
|  |  |  |  |  |  |  |  |
|  |  |  | **[1]<->[2]** | **[11]<->[12]** | **[11+]<->[22]** | **[11]<->[12+22]** | **odds ratio** |
|  |  |  | OR=0.980 | OR=1.004 | OR=0.954 | OR=0.990 | OR=0.977 |
|  |  |  | C.I.=[0.830-1.157] | C.I.=[0.754-1.336] | C.I.=[0.674-1.352] | C.I.=[0.753-1.300] |  |
|  |  |  | chi2=0.06 | chi2=0.00 | chi2=0.07 | chi2=0.01 | chi2=0.06 |
|  |  |  | p=0.80933 (P) | p=0.97932 | p=0.79223 | p=0.93974 | p=0.80136 |
|  |  |  | **Risk allele 1** | | | | |
|  |  |  | **[2]<->[1]** | **[22]<->[12]** | **[22]<->[11]** | **[11+12]<->[22]** | **odds ratio** |
|  |  |  | OR=1.021 | OR=1.052 | OR=1.048 | OR=1.051 | OR=1.023 |
|  |  |  | C.I.=[0.864-1.206] | C.I.=[0.778-1.422] | C.I.=[0.740-1.485] | C.I.=[0.788-1.401] |  |
|  |  |  | chi2=0.06 | chi2=0.11 | chi2=0.07 | chi2=0.11 | chi2=0.06 |
|  |  |  | p=0.80933 (P) | p=0.74223 | p=0.79223 | p=0.73660 | p=0.80136 |
| rs2075598 | 1.000 | 1.000 | **Risk allele 2** | | | | |
|  |  |  |  |  |  |  |  |
|  |  |  |  |  |  |  |  |
|  |  |  | **[1]<->[2]** | **[11]<->[12]** | **[11+]<->[22]** | **[11]<->[12+22]** | **odds ratio** |
|  |  |  | OR=0.581 | OR=0.576 | OR=1.727 | OR=0.576 | OR=0.576 |
|  |  |  | C.I.=[0.272-1.240] | C.I.=[0.268-1.237] | C.I.=[0.034-87.217] | C.I.=[0.268-1.237] |  |
|  |  |  | chi2=2.02 | chi2=2.05 | chi2=nan | chi2=2.05 | chi2=2.05 |
|  |  |  | p=0.15531 (P) | p=0.15216 | p=1.00000 | p=0.15216 | p=0.15216 |
|  |  |  | **Risk allele 1** | | | | |
|  |  |  | **[2]<->[1]** | **[22]<->[12]** | **[22]<->[11]** | **[11+12]<->[22]** | **odds ratio** |
|  |  |  | OR=1.723 | OR=0.345 | OR=0.579 | OR=0.570 | OR=1.485 |
|  |  |  | C.I.=[0.806-3.680] | C.I.=[0.006-18.650] | C.I.=[0.011-29.226] | C.I.=[0.011-28.788] |  |
|  |  |  | chi2=2.02 | chi2=nan | chi2=nan | chi2=nan | chi2=2.05 |
|  |  |  | p=0.15531 (P) | p=1.00000 | p=1.00000 | p=1.00000 | p=0.15216 |
| rs4901200 | 0.525 | 0.819 | **Risk allele 2** | | | | |
|  |  |  |  |  |  |  |  |
|  |  |  |  |  |  |  |  |
|  |  |  | **[1]<->[2]** | **[11]<->[12]** | **[11+]<->[22]** | **[11]<->[12+22]** | **odds ratio** |
|  |  |  | OR=0.901 | OR=0.952 | OR=0.574 | OR=0.921 | OR=0.881 |
|  |  |  | C.I.=[0.699-1.160] | C.I.=[0.715-1.266] | C.I.=[0.207-1.593] | C.I.=[0.697-1.217] |  |
|  |  |  | chi2=0.66 | chi2=0.12 | chi2=1.17 | chi2=0.34 | chi2=0.66 |
|  |  |  | p=0.41689 (P) | p=0.73364 | p=0.28023 | p=0.56252 | p=0.41822 |
|  |  |  | **Risk allele 1** | | | | |
|  |  |  | **[2]<->[1]** | **[22]<->[12]** | **[22]<->[11]** | **[11+12]<->[22]** | **odds ratio** |
|  |  |  | OR=1.110 | OR=1.659 | OR=1.743 | OR=1.724 | OR=1.146 |
|  |  |  | C.I.=[0.862-1.430] | C.I.=[0.585-4.707] | C.I.=[0.628-4.839] | C.I.=[0.622-4.776] |  |
|  |  |  | chi2=0.66 | chi2=0.92 | chi2=1.17 | chi2=1.12 | chi2=0.66 |
|  |  |  | p=0.41689 (P) | p=0.33722 | p=0.28023 | p=0.28912 | p=0.41822 |
| rs7150275 | 0.681 | 0.108 | **Risk allele 2** | | | | |
|  |  |  |  |  |  |  |  |
|  |  |  |  |  |  |  |  |
|  |  |  | **[1]<->[2]** | **[11]<->[12]** | **[11+]<->[22]** | **[11]<->[12+22]** | **odds ratio** |
|  |  |  | OR=1.033 | OR=0.950 | OR=1.249 | OR=0.991 | OR=1.060 |
|  |  |  | C.I.=[0.849-1.259] | C.I.=[0.737-1.224] | C.I.=[0.763-2.043] | C.I.=[0.779-1.260] |  |
|  |  |  | chi2=0.11 | chi2=0.16 | chi2=0.78 | chi2=0.01 | chi2=0.10 |
|  |  |  | p=0.74369 (P) | p=0.69101 | p=0.37613 | p=0.93969 | p=0.74841 |
|  |  |  | **Risk allele 1** | | | | |
|  |  |  | **[2]<->[1]** | **[22]<->[12]** | **[22]<->[11]** | **[11+12]<->[22]** | **odds ratio** |
|  |  |  | OR=0.968 | OR=0.761 | OR=0.801 | OR=0.786 | OR=0.946 |
|  |  |  | C.I.=[0.795-1.178] | C.I.=[0.457-1.268] | C.I.=[0.489-1.311] | C.I.=[0.485-1.276] |  |
|  |  |  | chi2=0.11 | chi2=1.11 | chi2=0.78 | chi2=0.95 | chi2=0.10 |
|  |  |  | p=0.74369 (P) | p=0.29292 | p=0.37613 | p=0.32899 | p=0.74841 |
| rs10141001 | 0.882 | 0.676 | **Risk allele 2** | | | | |
|  |  |  |  |  |  |  |  |
|  |  |  |  |  |  |  |  |
|  |  |  | **[1]<->[2]** | **[11]<->[12]** | **[11+]<->[22]** | **[11]<->[12+22]** | **odds ratio** |
|  |  |  | OR=0.901 | OR=0.936 | OR=0.643 | OR=0.913 | OR=0.886 |
|  |  |  | C.I.=[0.706-1.150] | C.I.=[0.709-1.236] | C.I.=[0.249-1.659] | C.I.=[0.696-1.197] |  |
|  |  |  | chi2=0.70 | chi2=0.22 | chi2=0.85 | chi2=0.44 | chi2=0.71 |
|  |  |  | p=0.40194 (P) | p=0.64048 | p=0.35734 | p=0.50832 | p=0.40084 |
|  |  |  | **Risk allele 1** | | | | |
|  |  |  | **[2]<->[1]** | **[22]<->[12]** | **[22]<->[11]** | **[11+12]<->[22]** | **odds ratio** |
|  |  |  | OR=1.110 | OR=1.456 | OR=1.556 | OR=1.531 | OR=1.135 |
|  |  |  | C.I.=[0.869-1.417] | C.I.=[0.552-3.837] | C.I.=[0.603-4.014] | C.I.=[0.595-3.942] |  |
|  |  |  | chi2=0.70 | chi2=0.58 | chi2=0.85 | chi2=0.79 | chi2=0.71 |
|  |  |  | p=0.40194 (P) | p=0.44524 | p=0.35734 | p=0.37401 | p=0.40084 |
| rs3794042 | 0.226 | 1.000 | **Risk allele 2** | | | | |
|  |  |  |  |  |  |  |  |
|  |  |  |  |  |  |  |  |
|  |  |  | **[1]<->[2]** | **[11]<->[12]** | **[11+]<->[22]** | **[11]<->[12+22]** | **odds ratio** |
|  |  |  | OR=1.011 | OR=1.083 | OR=0.988 | OR=1.058 | OR=1.003 |
|  |  |  | C.I.=[0.853-1.199] | C.I.=[0.836-1.403] | C.I.=[0.695-1.403] | C.I.=[0.829-1.350] |  |
|  |  |  | chi2=0.02 | chi2=0.37 | chi2=0.00 | chi2=0.20 | chi2=0.02 |
|  |  |  | p=0.89753 (P) | p=0.54448 | p=0.94413 | p=0.65172 | p=0.89901 |
|  |  |  | **Risk allele 1** | | | | |
|  |  |  | **[2]<->[1]** | **[22]<->[12]** | **[22]<->[11]** | **[11+12]<->[22]** | **odds ratio** |
|  |  |  | OR=0.989 | OR=1.097 | OR=1.013 | OR=1.059 | OR=0.997 |
|  |  |  | C.I.=[0.834-1.172] | C.I.=[0.781-1.541] | C.I.=[0.713-1.439] | C.I.=[0.769-1.458] |  |
|  |  |  | chi2=0.02 | chi2=0.29 | chi2=0.00 | chi2=0.12 | chi2=0.02 |
|  |  |  | p=0.89753 (P) | p=0.59330 | p=0.94413 | p=0.72651 | p=0.89901 |
| rs1874569 | 0.213 | 0.300 | **Risk allele 2** | | | | |
|  |  |  |  |  |  |  |  |
|  |  |  |  |  |  |  |  |
|  |  |  | **[1]<->[2]** | **[11]<->[12]** | **[11+]<->[22]** | **[11]<->[12+22]** | **odds ratio** |
|  |  |  | OR=0.955 | OR=0.838 | OR=1.186 | OR=0.881 | OR=0.997 |
|  |  |  | C.I.=[0.787-1.158] | C.I.=[0.653-1.076] | C.I.=[0.724-1.943] | C.I.=[0.694-1.117] |  |
|  |  |  | chi2=0.22 | chi2=1.93 | chi2=0.46 | chi2=1.10 | chi2=0.23 |
|  |  |  | p=0.63672 (P) | p=0.16512 | p=0.49846 | p=0.29399 | p=0.63450 |
|  |  |  | **Risk allele 1** | | | | |
|  |  |  | **[2]<->[1]** | **[22]<->[12]** | **[22]<->[11]** | **[11+12]<->[22]** | **odds ratio** |
|  |  |  | OR=1.048 | OR=0.707 | OR=0.843 | OR=0.786 | OR=1.006 |
|  |  |  | C.I.=[0.864-1.270] | C.I.=[0.426-1.174] | C.I.=[0.515-1.382] | C.I.=[0.485-1.276] |  |
|  |  |  | chi2=0.22 | chi2=1.81 | chi2=0.46 | chi2=0.95 | chi2=0.23 |
|  |  |  | p=0.63672 (P) | p=0.17878 | p=0.49846 | p=0.32899 | p=0.63450 |
| rs2509712 | 0.548 | 0.102 | **Risk allele 2** | | | | |
|  |  |  |  |  |  |  |  |
|  |  |  |  |  |  |  |  |
|  |  |  | **[1]<->[2]** | **[11]<->[12]** | **[11+]<->[22]** | **[11]<->[12+22]** | **odds ratio** |
|  |  |  | OR=1.127 | OR=1.210 | OR=0.974 | OR=1.190 | OR=1.102 |
|  |  |  | C.I.=[0.914-1.390] | C.I.=[0.942-1.556] | C.I.=[0.477-1.988] | C.I.=[0.931-1.519] |  |
|  |  |  | chi2=1.25 | chi2=2.22 | chi2=0.01 | chi2=1.93 | chi2=1.31 |
|  |  |  | p=0.26313 (P) | p=0.13604 | p=0.94268 | p=0.16436 | p=0.25150 |
|  |  |  | **Risk allele 1** | | | | |
|  |  |  | **[2]<->[1]** | **[22]<->[12]** | **[22]<->[11]** | **[11+12]<->[22]** | **odds ratio** |
|  |  |  | OR=0.887 | OR=1.243 | OR=1.027 | OR=1.095 | OR=0.908 |
|  |  |  | C.I.=[0.719-1.094] | C.I.=[0.601-2.571] | C.I.=[0.503-2.095] | C.I.=[0.540-2.223] |  |
|  |  |  | chi2=1.25 | chi2=0.34 | chi2=0.01 | chi2=0.06 | chi2=1.31 |
|  |  |  | p=0.26313 (P) | p=0.55757 | p=0.94268 | p=0.80113 | p=0.25150 |
| rs7149810 | 0.310 | 0.067 | **Risk allele 2** | | | | |
|  |  |  |  |  |  |  |  |
|  |  |  |  |  |  |  |  |
|  |  |  | **[1]<->[2]** | **[11]<->[12]** | **[11+]<->[22]** | **[11]<->[12+22]** | **odds ratio** |
|  |  |  | OR=1.047 | OR=0.977 | OR=1.227 | OR=1.014 | OR=1.066 |
|  |  |  | C.I.=[0.861-1.274] | C.I.=[0.758-1.259] | C.I.=[0.762-1.976] | C.I.=[0.798-1.289] |  |
|  |  |  | chi2=0.21 | chi2=0.03 | chi2=0.71 | chi2=0.01 | chi2=0.20 |
|  |  |  | p=0.64399 (P) | p=0.85442 | p=0.39834 | p=0.90944 | p=0.65289 |
|  |  |  | **Risk allele 1** | | | | |
|  |  |  | **[2]<->[1]** | **[22]<->[12]** | **[22]<->[11]** | **[11+12]<->[22]** | **odds ratio** |
|  |  |  | OR=0.955 | OR=0.796 | OR=0.815 | OR=0.808 | OR=0.940 |
|  |  |  | C.I.=[0.785-1.161] | C.I.=[0.485-1.305] | C.I.=[0.506-1.312] | C.I.=[0.506-1.289] |  |
|  |  |  | chi2=0.21 | chi2=0.82 | chi2=0.71 | chi2=0.80 | chi2=0.20 |
|  |  |  | p=0.64399 (P) | p=0.36422 | p=0.39834 | p=0.36974 | p=0.65289 |
| rs7167 | 0.217 | 1.000 | **Risk allele 2** | | | | |
|  |  |  |  |  |  |  |  |
|  |  |  |  |  |  |  |  |
|  |  |  | **[1]<->[2]** | **[11]<->[12]** | **[11+]<->[22]** | **[11]<->[12+22]** | **odds ratio** |
|  |  |  | OR=1.014 | OR=1.080 | OR=0.895 | OR=1.052 | OR=0.993 |
|  |  |  | C.I.=[0.832-1.235] | C.I.=[0.840-1.388] | C.I.=[0.531-1.511] | C.I.=[0.828-1.337] |  |
|  |  |  | chi2=0.02 | chi2=0.36 | chi2=0.17 | chi2=0.17 | chi2=0.02 |
|  |  |  | p=0.89291 (P) | p=0.54766 | p=0.67868 | p=0.67874 | p=0.89440 |
|  |  |  | **Risk allele 1** | | | | |
|  |  |  | **[2]<->[1]** | **[22]<->[12]** | **[22]<->[11]** | **[11+12]<->[22]** | **odds ratio** |
|  |  |  | OR=0.987 | OR=1.206 | OR=1.117 | OR=1.149 | OR=1.008 |
|  |  |  | C.I.=[0.810-1.202] | C.I.=[0.704-2.067] | C.I.=[0.662-1.885] | C.I.=[0.686-1.923] |  |
|  |  |  | chi2=0.02 | chi2=0.47 | chi2=0.17 | chi2=0.28 | chi2=0.02 |
|  |  |  | p=0.89291 (P) | p=0.49475 | p=0.67868 | p=0.59722 | p=0.89440 |
| rs3742536 | 0.465 | 0.477 | **Risk allele 2** | | | | |
|  |  |  |  |  |  |  |  |
|  |  |  |  |  |  |  |  |
|  |  |  | **[1]<->[2]** | **[11]<->[12]** | **[11+]<->[22]** | **[11]<->[12+22]** | **odds ratio** |
|  |  |  | OR=0.945 | OR=0.955 | OR=0.858 | OR=0.944 | OR=0.939 |
|  |  |  | C.I.=[0.773-1.157] | C.I.=[0.744-1.225] | C.I.=[0.470-1.565] | C.I.=[0.742-1.201] |  |
|  |  |  | chi2=0.30 | chi2=0.13 | chi2=0.25 | chi2=0.22 | chi2=0.31 |
|  |  |  | p=0.58563 (P) | p=0.71771 | p=0.61657 | p=0.64085 | p=0.57928 |
|  |  |  | **Risk allele 1** | | | | |
|  |  |  | **[2]<->[1]** | **[22]<->[12]** | **[22]<->[11]** | **[11+12]<->[22]** | **odds ratio** |
|  |  |  | OR=1.058 | OR=1.114 | OR=1.166 | OR=1.146 | OR=1.065 |
|  |  |  | C.I.=[0.864-1.294] | C.I.=[0.602-2.060] | C.I.=[0.639-2.128] | C.I.=[0.633-2.077] |  |
|  |  |  | chi2=0.30 | chi2=0.12 | chi2=0.25 | chi2=0.20 | chi2=0.31 |
|  |  |  | p=0.58563 (P) | p=0.73170 | p=0.61657 | p=0.65220 | p=0.57928 |
| rs12895034 | 0.518 | 0.240 | **Risk allele 2** | | | | |
|  |  |  |  |  |  |  |  |
|  |  |  |  |  |  |  |  |
|  |  |  | **[1]<->[2]** | **[11]<->[12]** | **[11+]<->[22]** | **[11]<->[12+22]** | **odds ratio** |
|  |  |  | OR=1.004 | OR=0.953 | OR=1.053 | OR=0.974 | OR=1.013 |
|  |  |  | C.I.=[0.842-1.197] | C.I.=[0.741-1.225] | C.I.=[0.723-1.533] | C.I.=[0.769-1.234] |  |
|  |  |  | chi2=0.00 | chi2=0.14 | chi2=0.07 | chi2=0.05 | chi2=0.00 |
|  |  |  | p=0.96476 (P) | p=0.70662 | p=0.78848 | p=0.82998 | p=0.96540 |
|  |  |  | **Risk allele 1** | | | | |
|  |  |  | **[2]<->[1]** | **[22]<->[12]** | **[22]<->[11]** | **[11+12]<->[22]** | **odds ratio** |
|  |  |  | OR=0.996 | OR=0.905 | OR=0.950 | OR=0.928 | OR=0.987 |
|  |  |  | C.I.=[0.836-1.187] | C.I.=[0.620-1.321] | C.I.=[0.652-1.383] | C.I.=[0.650-1.323] |  |
|  |  |  | chi2=0.00 | chi2=0.27 | chi2=0.07 | chi2=0.17 | chi2=0.00 |
|  |  |  | p=0.96476 (P) | p=0.60467 | p=0.78848 | p=0.67839 | p=0.96540 |
| rs17694496 | 0.166 | 0.418 | **Risk allele 2** | | | | |
|  |  |  |  |  |  |  |  |
|  |  |  |  |  |  |  |  |
|  |  |  | **[1]<->[2]** | **[11]<->[12]** | **[11+]<->[22]** | **[11]<->[12+22]** | **odds ratio** |
|  |  |  | OR=0.802 | OR=0.713 | OR=0.920 | OR=0.733 | OR=0.837 |
|  |  |  | C.I.=[0.649-0.990] | C.I.=[0.551-0.922] | C.I.=[0.497-1.704] | C.I.=[0.572-0.939] |  |
|  |  |  | chi2=4.23 | chi2=6.65 | chi2=0.07 | chi2=6.08 | chi2=4.32 |
|  |  |  | p=0.03967 (P) | p=0.00989 | p=0.79093 | p=0.01369 | p=0.03776 |
|  |  |  | **Risk allele 1** | | | | |
|  |  |  | **[2]<->[1]** | **[22]<->[12]** | **[22]<->[11]** | **[11+12]<->[22]** | **odds ratio** |
|  |  |  | OR=1.248 | OR=0.775 | OR=1.087 | OR=0.971 | OR=1.192 |
|  |  |  | C.I.=[1.010-1.541] | C.I.=[0.411-1.462] | C.I.=[0.587-2.013] | C.I.=[0.527-1.788] |  |
|  |  |  | chi2=4.23 | chi2=0.62 | chi2=0.07 | chi2=0.01 | chi2=4.32 |
|  |  |  | p=0.03967 (P) | p=0.43027 | p=0.79093 | p=0.92513 | p=0.03776 |
| rs1201378 | 0.453 | 0.058 | **Risk allele 2** | | | | |
|  |  |  |  |  |  |  |  |
|  |  |  |  |  |  |  |  |
|  |  |  | **[1]<->[2]** | **[11]<->[12]** | **[11+]<->[22]** | **[11]<->[12+22]** | **odds ratio** |
|  |  |  | OR=0.983 | OR=0.802 | OR=1.061 | OR=0.864 | OR=1.004 |
|  |  |  | C.I.=[0.829-1.165] | C.I.=[0.618-1.040] | C.I.=[0.752-1.497] | C.I.=[0.677-1.102] |  |
|  |  |  | chi2=0.04 | chi2=2.78 | chi2=0.12 | chi2=1.39 | chi2=0.04 |
|  |  |  | p=0.84054 (P) | p=0.09547 | p=0.73421 | p=0.23811 | p=0.84166 |
|  |  |  | **Risk allele 1** | | | | |
|  |  |  | **[2]<->[1]** | **[22]<->[12]** | **[22]<->[11]** | **[11+12]<->[22]** | **odds ratio** |
|  |  |  | OR=1.018 | OR=0.755 | OR=0.942 | OR=0.832 | OR=0.996 |
|  |  |  | C.I.=[0.859-1.206] | C.I.=[0.541-1.054] | C.I.=[0.668-1.329] | C.I.=[0.609-1.138] |  |
|  |  |  | chi2=0.04 | chi2=2.73 | chi2=0.12 | chi2=1.33 | chi2=0.04 |
|  |  |  | p=0.84054 (P) | p=0.09840 | p=0.73421 | p=0.24956 | p=0.84166 |
| rs803012 | 0.837 | 0.573 | **Risk allele 2** | | | | |
|  |  |  |  |  |  |  |  |
|  |  |  |  |  |  |  |  |
|  |  |  | **[1]<->[2]** | **[11]<->[12]** | **[11+]<->[22]** | **[11]<->[12+22]** | **odds ratio** |
|  |  |  | OR=0.943 | OR=0.971 | OR=0.815 | OR=0.952 | OR=0.932 |
|  |  |  | C.I.=[0.772-1.153] | C.I.=[0.757-1.247] | C.I.=[0.456-1.457] | C.I.=[0.749-1.211] |  |
|  |  |  | chi2=0.32 | chi2=0.05 | chi2=0.48 | chi2=0.16 | chi2=0.33 |
|  |  |  | p=0.56928 (P) | p=0.82022 | p=0.48967 | p=0.69009 | p=0.56570 |
|  |  |  | **Risk allele 1** | | | | |
|  |  |  | **[2]<->[1]** | **[22]<->[12]** | **[22]<->[11]** | **[11+12]<->[22]** | **odds ratio** |
|  |  |  | OR=1.060 | OR=1.192 | OR=1.227 | OR=1.214 | OR=1.074 |
|  |  |  | C.I.=[0.867-1.296] | C.I.=[0.658-2.160] | C.I.=[0.686-2.193] | C.I.=[0.684-2.154] |  |
|  |  |  | chi2=0.32 | chi2=0.34 | chi2=0.48 | chi2=0.44 | chi2=0.33 |
|  |  |  | p=0.56928 (P) | p=0.56262 | p=0.48967 | p=0.50730 | p=0.56570 |
| rs708502 | 0.881 | 0.229 | **Risk allele 2** | | | | |
|  |  |  |  |  |  |  |  |
|  |  |  |  |  |  |  |  |
|  |  |  | **[1]<->[2]** | **[11]<->[12]** | **[11+]<->[22]** | **[11]<->[12+22]** | **odds ratio** |
|  |  |  | OR=0.980 | OR=1.039 | OR=0.628 | OR=1.010 | OR=0.955 |
|  |  |  | C.I.=[0.771-1.246] | C.I.=[0.792-1.363] | C.I.=[0.224-1.761] | C.I.=[0.774-1.319] |  |
|  |  |  | chi2=0.03 | chi2=0.08 | chi2=0.79 | chi2=0.01 | chi2=0.03 |
|  |  |  | p=0.86982 (P) | p=0.78379 | p=0.37265 | p=0.93970 | p=0.86771 |
|  |  |  | **Risk allele 1** | | | | |
|  |  |  | **[2]<->[1]** | **[22]<->[12]** | **[22]<->[11]** | **[11+12]<->[22]** | **odds ratio** |
|  |  |  | OR=1.020 | OR=1.653 | OR=1.591 | OR=1.607 | OR=1.057 |
|  |  |  | C.I.=[0.803-1.297] | C.I.=[0.580-4.713] | C.I.=[0.568-4.459] | C.I.=[0.575-4.492] |  |
|  |  |  | chi2=0.03 | chi2=0.90 | chi2=0.79 | chi2=0.83 | chi2=0.03 |
|  |  |  | p=0.86982 (P) | p=0.34262 | p=0.37265 | p=0.36148 | p=0.86771 |
| rs1209087 | 0.548 | 0.022 | **Risk allele 2** | | | | |
|  |  |  |  |  |  |  |  |
|  |  |  |  |  |  |  |  |
|  |  |  | **[1]<->[2]** | **[11]<->[12]** | **[11+]<->[22]** | **[11]<->[12+22]** | **odds ratio** |
|  |  |  | OR=1.016 | OR=0.806 | OR=1.137 | OR=0.886 | OR=1.039 |
|  |  |  | C.I.=[0.858-1.204] | C.I.=[0.620-1.046] | C.I.=[0.810-1.597] | C.I.=[0.694-1.130] |  |
|  |  |  | chi2=0.03 | chi2=2.63 | chi2=0.55 | chi2=0.95 | chi2=0.03 |
|  |  |  | p=0.85363 (P) | p=0.10500 | p=0.45873 | p=0.32919 | p=0.85543 |
|  |  |  | **Risk allele 1** | | | | |
|  |  |  | **[2]<->[1]** | **[22]<->[12]** | **[22]<->[11]** | **[11+12]<->[22]** | **odds ratio** |
|  |  |  | OR=0.984 | OR=0.709 | OR=0.880 | OR=0.780 | OR=0.964 |
|  |  |  | C.I.=[0.831-1.166] | C.I.=[0.510-0.985] | C.I.=[0.626-1.235] | C.I.=[0.573-1.060] |  |
|  |  |  | chi2=0.03 | chi2=4.22 | chi2=0.55 | chi2=2.52 | chi2=0.03 |
|  |  |  | p=0.85363 (P) | p=0.03994 | p=0.45873 | p=0.11213 | p=0.85543 |
| rs17125273 | 1.000 | 0.027 | **Risk allele 2** | | | | |
|  |  |  |  |  |  |  |  |
|  |  |  |  |  |  |  |  |
|  |  |  | **[1]<->[2]** | **[11]<->[12]** | **[11+]<->[22]** | **[11]<->[12+22]** | **odds ratio** |
|  |  |  | OR=1.145 | OR=0.990 | OR=1.990 | OR=1.073 | OR=1.210 |
|  |  |  | C.I.=[0.915-1.433] | C.I.=[0.754-1.299] | C.I.=[1.037-3.819] | C.I.=[0.828-1.389] |  |
|  |  |  | chi2=1.40 | chi2=0.01 | chi2=4.43 | chi2=0.28 | chi2=1.34 |
|  |  |  | p=0.23660 (P) | p=0.94171 | p=0.03536 | p=0.59383 | p=0.24678 |
|  |  |  | **Risk allele 1** | | | | |
|  |  |  | **[2]<->[1]** | **[22]<->[12]** | **[22]<->[11]** | **[11+12]<->[22]** | **odds ratio** |
|  |  |  | OR=0.873 | OR=0.497 | OR=0.503 | OR=0.501 | OR=0.843 |
|  |  |  | C.I.=[0.698-1.093] | C.I.=[0.253-0.980] | C.I.=[0.262-0.965] | C.I.=[0.262-0.958] |  |
|  |  |  | chi2=1.40 | chi2=4.19 | chi2=4.43 | chi2=4.52 | chi2=1.34 |
|  |  |  | p=0.23660 (P) | p=0.04066 | p=0.03536 | p=0.03341 | p=0.24678 |
| rs1565970 | 1.000 | 1.000 | **Risk allele 2** | | | | |
|  |  |  |  |  |  |  |  |
|  |  |  |  |  |  |  |  |
|  |  |  | **[1]<->[2]** | **[11]<->[12]** | **[11+]<->[22]** | **[11]<->[12+22]** | **odds ratio** |
|  |  |  | OR=0.861 | OR=0.869 | OR=0.574 | OR=0.860 | OR=0.853 |
|  |  |  | C.I.=[0.614-1.206] | C.I.=[0.608-1.241] | C.I.=[0.059-5.533] | C.I.=[0.604-1.224] |  |
|  |  |  | chi2=0.76 | chi2=0.60 | chi2=0.24 | chi2=0.70 | chi2=0.78 |
|  |  |  | p=0.38272 (P) | p=0.43851 | p=0.62636 | p=0.40313 | p=0.37787 |
|  |  |  | **Risk allele 1** | | | | |
|  |  |  | **[2]<->[1]** | **[22]<->[12]** | **[22]<->[11]** | **[11+12]<->[22]** | **odds ratio** |
|  |  |  | OR=1.162 | OR=1.515 | OR=1.744 | OR=1.713 | OR=1.176 |
|  |  |  | C.I.=[0.829-1.628] | C.I.=[0.154-14.920] | C.I.=[0.181-16.820] | C.I.=[0.178-16.515] |  |
|  |  |  | chi2=0.76 | chi2=0.13 | chi2=0.24 | chi2=0.22 | chi2=0.78 |
|  |  |  | p=0.38272 (P) | p=0.72028 | p=0.62636 | p=0.63772 | p=0.37787 |
| rs1957844 | 0.912 | 0.781 | **Risk allele 2** | | | | |
|  |  |  |  |  |  |  |  |
|  |  |  |  |  |  |  |  |
|  |  |  | **[1]<->[2]** | **[11]<->[12]** | **[11+]<->[22]** | **[11]<->[12+22]** | **odds ratio** |
|  |  |  | OR=1.093 | OR=1.064 | OR=1.286 | OR=1.088 | OR=1.105 |
|  |  |  | C.I.=[0.893-1.339] | C.I.=[0.827-1.369] | C.I.=[0.730-2.266] | C.I.=[0.855-1.387] |  |
|  |  |  | chi2=0.75 | chi2=0.23 | chi2=0.76 | chi2=0.47 | chi2=0.75 |
|  |  |  | p=0.38741 (P) | p=0.62865 | p=0.38321 | p=0.49224 | p=0.38706 |
|  |  |  | **Risk allele 1** | | | | |
|  |  |  | **[2]<->[1]** | **[22]<->[12]** | **[22]<->[11]** | **[11+12]<->[22]** | **odds ratio** |
|  |  |  | OR=0.915 | OR=0.828 | OR=0.778 | OR=0.795 | OR=0.906 |
|  |  |  | C.I.=[0.747-1.120] | C.I.=[0.462-1.482] | C.I.=[0.441-1.370] | C.I.=[0.454-1.391] |  |
|  |  |  | chi2=0.75 | chi2=0.41 | chi2=0.76 | chi2=0.65 | chi2=0.75 |
|  |  |  | p=0.38741 (P) | p=0.52405 | p=0.38321 | p=0.42013 | p=0.38706 |
| rs17666653 | 0.738 | 0.165 | **Risk allele 2** | | | | |
|  |  |  |  |  |  |  |  |
|  |  |  |  |  |  |  |  |
|  |  |  | **[1]<->[2]** | **[11]<->[12]** | **[11+]<->[22]** | **[11]<->[12+22]** | **odds ratio** |
|  |  |  | OR=1.114 | OR=1.236 | OR=0.912 | OR=1.197 | OR=1.075 |
|  |  |  | C.I.=[0.910-1.364] | C.I.=[0.963-1.586] | C.I.=[0.492-1.688] | C.I.=[0.940-1.524] |  |
|  |  |  | chi2=1.09 | chi2=2.77 | chi2=0.09 | chi2=2.14 | chi2=1.11 |
|  |  |  | p=0.29582 (P) | p=0.09630 | p=0.76842 | p=0.14368 | p=0.29107 |
|  |  |  | **Risk allele 1** | | | | |
|  |  |  | **[2]<->[1]** | **[22]<->[12]** | **[22]<->[11]** | **[11+12]<->[22]** | **odds ratio** |
|  |  |  | OR=0.898 | OR=1.355 | OR=1.097 | OR=1.183 | OR=0.932 |
|  |  |  | C.I.=[0.733-1.099] | C.I.=[0.722-2.543] | C.I.=[0.593-2.031] | C.I.=[0.644-2.176] |  |
|  |  |  | chi2=1.09 | chi2=0.90 | chi2=0.09 | chi2=0.29 | chi2=1.11 |
|  |  |  | p=0.29582 (P) | p=0.34226 | p=0.76842 | p=0.58747 | p=0.29107 |
| rs17128136 | 0.130 | 0.034 | **Risk allele 2** | | | | |
|  |  |  |  |  |  |  |  |
|  |  |  |  |  |  |  |  |
|  |  |  | **[1]<->[2]** | **[11]<->[12]** | **[11+]<->[22]** | **[11]<->[12+22]** | **odds ratio** |
|  |  |  | OR=1.134 | OR=1.063 | OR=1.607 | OR=1.104 | OR=1.153 |
|  |  |  | C.I.=[0.858-1.498] | C.I.=[0.772-1.463] | C.I.=[0.647-3.993] | C.I.=[0.813-1.500] |  |
|  |  |  | chi2=0.78 | chi2=0.14 | chi2=1.06 | chi2=0.40 | chi2=0.73 |
|  |  |  | p=0.37689 (P) | p=0.70897 | p=0.30237 | p=0.52513 | p=0.39420 |
|  |  |  | **Risk allele 1** | | | | |
|  |  |  | **[2]<->[1]** | **[22]<->[12]** | **[22]<->[11]** | **[11+12]<->[22]** | **odds ratio** |
|  |  |  | OR=0.882 | OR=0.661 | OR=0.622 | OR=0.628 | OR=0.874 |
|  |  |  | C.I.=[0.668-1.165] | C.I.=[0.257-1.704] | C.I.=[0.250-1.545] | C.I.=[0.253-1.559] |  |
|  |  |  | chi2=0.78 | chi2=0.74 | chi2=1.06 | chi2=1.02 | chi2=0.73 |
|  |  |  | p=0.37689 (P) | p=0.38907 | p=0.30237 | p=0.31199 | p=0.39420 |
| rs12587410 | 0.189 | 0.467 | **Risk allele 2** | | | | |
|  |  |  |  |  |  |  |  |
|  |  |  |  |  |  |  |  |
|  |  |  | **[1]<->[2]** | **[11]<->[12]** | **[11+]<->[22]** | **[11]<->[12+22]** | **odds ratio** |
|  |  |  | OR=1.164 | OR=1.196 | OR=0.887 | OR=1.183 | OR=1.141 |
|  |  |  | C.I.=[0.744-1.822] | C.I.=[0.746-1.916] | C.I.=[0.080-9.816] | C.I.=[0.744-1.881] |  |
|  |  |  | chi2=0.44 | chi2=0.55 | chi2=0.01 | chi2=0.51 | chi2=0.43 |
|  |  |  | p=0.50518 (P) | p=0.45630 | p=0.92234 | p=0.47662 | p=0.51330 |
|  |  |  | **Risk allele 1** | | | | |
|  |  |  | **[2]<->[1]** | **[22]<->[12]** | **[22]<->[11]** | **[11+12]<->[22]** | **odds ratio** |
|  |  |  | OR=0.859 | OR=1.348 | OR=1.127 | OR=1.140 | OR=0.878 |
|  |  |  | C.I.=[0.549-1.344] | C.I.=[0.117-15.514] | C.I.=[0.102-12.466] | C.I.=[0.103-12.611] |  |
|  |  |  | chi2=0.44 | chi2=0.06 | chi2=0.01 | chi2=0.01 | chi2=0.43 |
|  |  |  | p=0.50518 (P) | p=0.81014 | p=0.92234 | p=0.91471 | p=0.51330 |
| rs17831682 | 0.028 | 0.101 | **Risk allele 2** | | | | |
|  |  |  |  |  |  |  |  |
|  |  |  |  |  |  |  |  |
|  |  |  | **[1]<->[2]** | **[11]<->[12]** | **[11+]<->[22]** | **[11]<->[12+22]** | **odds ratio** |
|  |  |  | OR=1.337 | OR=1.125 | OR=27.225 | OR=1.238 | OR=1.775 |
|  |  |  | C.I.=[0.995-1.796] | C.I.=[0.813-1.557] | C.I.=[1.550-478.067] | C.I.=[0.901-1.699] |  |
|  |  |  | chi2=3.73 | chi2=0.51 | chi2=12.56 | chi2=1.74 | chi2=3.77 |
|  |  |  | p=0.05356 (P) | p=0.47684 | p=0.00039 | p=0.18702 | p=0.05214 |
|  |  |  | **Risk allele 1** | | | | |
|  |  |  | **[2]<->[1]** | **[22]<->[12]** | **[22]<->[11]** | **[11+12]<->[22]** | **odds ratio** |
|  |  |  | OR=0.748 | OR=0.041 | OR=0.037 | OR=0.037 | OR=0.752 |
|  |  |  | C.I.=[0.557-1.005] | C.I.=[0.002-0.736] | C.I.=[0.002-0.645] | C.I.=[0.002-0.657] |  |
|  |  |  | chi2=3.73 | chi2=10.67 | chi2=12.56 | chi2=12.35 | chi2=3.77 |
|  |  |  | p=0.05356 (P) | p=0.00109 | p=0.00039 | p=0.00044 | p=0.05214 |
| rs17666689 | 0.894 | 0.510 | **Risk allele 2** | | | | |
|  |  |  |  |  |  |  |  |
|  |  |  |  |  |  |  |  |
|  |  |  | **[1]<->[2]** | **[11]<->[12]** | **[11+]<->[22]** | **[11]<->[12+22]** | **odds ratio** |
|  |  |  | OR=1.102 | OR=1.068 | OR=1.352 | OR=1.093 | OR=1.115 |
|  |  |  | C.I.=[0.883-1.376] | C.I.=[0.819-1.391] | C.I.=[0.682-2.680] | C.I.=[0.847-1.410] |  |
|  |  |  | chi2=0.74 | chi2=0.23 | chi2=0.75 | chi2=0.47 | chi2=0.73 |
|  |  |  | p=0.39118 (P) | p=0.62820 | p=0.38604 | p=0.49520 | p=0.39405 |
|  |  |  | **Risk allele 1** | | | | |
|  |  |  | **[2]<->[1]** | **[22]<->[12]** | **[22]<->[11]** | **[11+12]<->[22]** | **odds ratio** |
|  |  |  | OR=0.908 | OR=0.790 | OR=0.740 | OR=0.753 | OR=0.899 |
|  |  |  | C.I.=[0.727-1.133] | C.I.=[0.390-1.600] | C.I.=[0.373-1.466] | C.I.=[0.382-1.487] |  |
|  |  |  | chi2=0.74 | chi2=0.43 | chi2=0.75 | chi2=0.67 | chi2=0.73 |
|  |  |  | p=0.39118 (P) | p=0.51126 | p=0.38604 | p=0.41316 | p=0.39405 |
| rs708486 | 0.466 | 0.126 | **Risk allele 2** | | | | |
|  |  |  |  |  |  |  |  |
|  |  |  |  |  |  |  |  |
|  |  |  | **[1]<->[2]** | **[11]<->[12]** | **[11+]<->[22]** | **[11]<->[12+22]** | **odds ratio** |
|  |  |  | OR=1.119 | OR=0.920 | OR=1.275 | OR=1.021 | OR=1.123 |
|  |  |  | C.I.=[0.948-1.322] | C.I.=[0.696-1.215] | C.I.=[0.918-1.772] | C.I.=[0.786-1.325] |  |
|  |  |  | chi2=1.76 | chi2=0.35 | chi2=2.10 | chi2=0.02 | chi2=1.74 |
|  |  |  | p=0.18494 (P) | p=0.55662 | p=0.14729 | p=0.87779 | p=0.18681 |
|  |  |  | **Risk allele 1** | | | | |
|  |  |  | **[2]<->[1]** | **[22]<->[12]** | **[22]<->[11]** | **[11+12]<->[22]** | **odds ratio** |
|  |  |  | OR=0.893 | OR=0.721 | OR=0.784 | OR=0.744 | OR=0.891 |
|  |  |  | C.I.=[0.756-1.055] | C.I.=[0.536-0.971] | C.I.=[0.564-1.090] | C.I.=[0.563-0.983] |  |
|  |  |  | chi2=1.76 | chi2=4.66 | chi2=2.10 | chi2=4.35 | chi2=1.74 |
|  |  |  | p=0.18494 (P) | p=0.03090 | p=0.14729 | p=0.03700 | p=0.18681 |
| rs9671722 | 0.141 | 0.308 | **Risk allele 2** | | | | |
|  |  |  |  |  |  |  |  |
|  |  |  |  |  |  |  |  |
|  |  |  | **[1]<->[2]** | **[11]<->[12]** | **[11+]<->[22]** | **[11]<->[12+22]** | **odds ratio** |
|  |  |  | OR=1.081 | OR=1.079 | OR=1.151 | OR=1.087 | OR=1.076 |
|  |  |  | C.I.=[0.865-1.351] | C.I.=[0.825-1.412] | C.I.=[0.605-2.190] | C.I.=[0.841-1.406] |  |
|  |  |  | chi2=0.47 | chi2=0.31 | chi2=0.18 | chi2=0.41 | chi2=0.44 |
|  |  |  | p=0.49423 (P) | p=0.57831 | p=0.66765 | p=0.52291 | p=0.50525 |
|  |  |  | **Risk allele 1** | | | | |
|  |  |  | **[2]<->[1]** | **[22]<->[12]** | **[22]<->[11]** | **[11+12]<->[22]** | **odds ratio** |
|  |  |  | OR=0.925 | OR=0.938 | OR=0.869 | OR=0.887 | OR=0.930 |
|  |  |  | C.I.=[0.740-1.156] | C.I.=[0.481-1.829] | C.I.=[0.457-1.652] | C.I.=[0.468-1.680] |  |
|  |  |  | chi2=0.47 | chi2=0.04 | chi2=0.18 | chi2=0.14 | chi2=0.44 |
|  |  |  | p=0.49423 (P) | p=0.84980 | p=0.66765 | p=0.71235 | p=0.50525 |
| rs1953861 | 0.702 | 0.921 | **Risk allele 2** | | | | |
|  |  |  |  |  |  |  |  |
|  |  |  |  |  |  |  |  |
|  |  |  | **[1]<->[2]** | **[11]<->[12]** | **[11+]<->[22]** | **[11]<->[12+22]** | **odds ratio** |
|  |  |  | OR=1.136 | OR=1.095 | OR=1.317 | OR=1.146 | OR=1.142 |
|  |  |  | C.I.=[0.959-1.346] | C.I.=[0.845-1.420] | C.I.=[0.924-1.876] | C.I.=[0.896-1.465] |  |
|  |  |  | chi2=2.16 | chi2=0.47 | chi2=2.33 | chi2=1.18 | chi2=2.18 |
|  |  |  | p=0.14129 (P) | p=0.49284 | p=0.12680 | p=0.27672 | p=0.13997 |
|  |  |  | **Risk allele 1** | | | | |
|  |  |  | **[2]<->[1]** | **[22]<->[12]** | **[22]<->[11]** | **[11+12]<->[22]** | **odds ratio** |
|  |  |  | OR=0.880 | OR=0.832 | OR=0.759 | OR=0.800 | OR=0.876 |
|  |  |  | C.I.=[0.743-1.043] | C.I.=[0.592-1.168] | C.I.=[0.533-1.082] | C.I.=[0.580-1.102] |  |
|  |  |  | chi2=2.16 | chi2=1.14 | chi2=2.33 | chi2=1.87 | chi2=2.18 |
|  |  |  | p=0.14129 (P) | p=0.28669 | p=0.12680 | p=0.17178 | p=0.13997 |
| rs17831675 | 0.170 | 0.269 | **Risk allele 2** | | | | |
|  |  |  |  |  |  |  |  |
|  |  |  |  |  |  |  |  |
|  |  |  | **[1]<->[2]** | **[11]<->[12]** | **[11+]<->[22]** | **[11]<->[12+22]** | **odds ratio** |
|  |  |  | OR=1.363 | OR=1.209 | OR=10.978 | OR=1.297 | OR=1.548 |
|  |  |  | C.I.=[1.013-1.833] | C.I.=[0.874-1.672] | C.I.=[1.316-91.540] | C.I.=[0.944-1.782] |  |
|  |  |  | chi2=4.21 | chi2=1.31 | chi2=7.69 | chi2=2.59 | chi2=4.25 |
|  |  |  | p=0.04016 (P) | p=0.25189 | p=0.00556 | p=0.10736 | p=0.03914 |
|  |  |  | **Risk allele 1** | | | | |
|  |  |  | **[2]<->[1]** | **[22]<->[12]** | **[22]<->[11]** | **[11+12]<->[22]** | **odds ratio** |
|  |  |  | OR=0.734 | OR=0.110 | OR=0.091 | OR=0.094 | OR=0.724 |
|  |  |  | C.I.=[0.546-0.987] | C.I.=[0.013-0.934] | C.I.=[0.011-0.760] | C.I.=[0.011-0.782] |  |
|  |  |  | chi2=4.21 | chi2=5.86 | chi2=7.69 | chi2=7.43 | chi2=4.25 |
|  |  |  | p=0.04016 (P) | p=0.01551 | p=0.00556 | p=0.00640 | p=0.03914 |
| rs1993839 | 0.819 | 0.757 | **Risk allele 2** | | | | |
|  |  |  |  |  |  |  |  |
|  |  |  |  |  |  |  |  |
|  |  |  | **[1]<->[2]** | **[11]<->[12]** | **[11+]<->[22]** | **[11]<->[12+22]** | **odds ratio** |
|  |  |  | OR=0.967 | OR=0.934 | OR=1.044 | OR=0.945 | OR=0.981 |
|  |  |  | C.I.=[0.784-1.194] | C.I.=[0.723-1.207] | C.I.=[0.562-1.942] | C.I.=[0.738-1.210] |  |
|  |  |  | chi2=0.10 | chi2=0.27 | chi2=0.02 | chi2=0.20 | chi2=0.10 |
|  |  |  | p=0.75618 (P) | p=0.60109 | p=0.89063 | p=0.65453 | p=0.75581 |
|  |  |  | **Risk allele 1** | | | | |
|  |  |  | **[2]<->[1]** | **[22]<->[12]** | **[22]<->[11]** | **[11+12]<->[22]** | **odds ratio** |
|  |  |  | OR=1.034 | OR=0.894 | OR=0.957 | OR=0.936 | OR=1.020 |
|  |  |  | C.I.=[0.838-1.276] | C.I.=[0.472-1.693] | C.I.=[0.515-1.780] | C.I.=[0.507-1.731] |  |
|  |  |  | chi2=0.10 | chi2=0.12 | chi2=0.02 | chi2=0.04 | chi2=0.10 |
|  |  |  | p=0.75618 (P) | p=0.73110 | p=0.89063 | p=0.83391 | p=0.75581 |
| rs555835 | 1.000 | 0.921 | **Risk allele 2** | | | | |
|  |  |  |  |  |  |  |  |
|  |  |  |  |  |  |  |  |
|  |  |  | **[1]<->[2]** | **[11]<->[12]** | **[11+]<->[22]** | **[11]<->[12+22]** | **odds ratio** |
|  |  |  | OR=0.909 | OR=0.898 | OR=0.831 | OR=0.880 | OR=0.910 |
|  |  |  | C.I.=[0.767-1.076] | C.I.=[0.691-1.165] | C.I.=[0.585-1.179] | C.I.=[0.688-1.126] |  |
|  |  |  | chi2=1.23 | chi2=0.66 | chi2=1.08 | chi2=1.04 | chi2=1.22 |
|  |  |  | p=0.26818 (P) | p=0.41706 | p=0.29866 | p=0.30808 | p=0.26845 |
|  |  |  | **Risk allele 1** | | | | |
|  |  |  | **[2]<->[1]** | **[22]<->[12]** | **[22]<->[11]** | **[11+12]<->[22]** | **odds ratio** |
|  |  |  | OR=1.100 | OR=1.081 | OR=1.204 | OR=1.131 | OR=1.099 |
|  |  |  | C.I.=[0.929-1.303] | C.I.=[0.773-1.510] | C.I.=[0.848-1.708] | C.I.=[0.824-1.550] |  |
|  |  |  | chi2=1.23 | chi2=0.21 | chi2=1.08 | chi2=0.58 | chi2=1.22 |
|  |  |  | p=0.26818 (P) | p=0.64992 | p=0.29866 | p=0.44631 | p=0.26845 |
| rs545659 | 0.160 | 0.686 | **Risk allele 2** | | | | |
|  |  |  |  |  |  |  |  |
|  |  |  |  |  |  |  |  |
|  |  |  | **[1]<->[2]** | **[11]<->[12]** | **[11+]<->[22]** | **[11]<->[12+22]** | **odds ratio** |
|  |  |  | OR=0.983 | OR=1.023 | OR=0.896 | OR=1.003 | OR=0.972 |
|  |  |  | C.I.=[0.808-1.197] | C.I.=[0.796-1.316] | C.I.=[0.541-1.486] | C.I.=[0.789-1.275] |  |
|  |  |  | chi2=0.03 | chi2=0.03 | chi2=0.18 | chi2=0.00 | chi2=0.03 |
|  |  |  | p=0.86776 (P) | p=0.85881 | p=0.67099 | p=0.98011 | p=0.87027 |
|  |  |  | **Risk allele 1** | | | | |
|  |  |  | **[2]<->[1]** | **[22]<->[12]** | **[22]<->[11]** | **[11+12]<->[22]** | **odds ratio** |
|  |  |  | OR=1.017 | OR=1.141 | OR=1.116 | OR=1.125 | OR=1.029 |
|  |  |  | C.I.=[0.835-1.238] | C.I.=[0.678-1.923] | C.I.=[0.673-1.849] | C.I.=[0.685-1.849] |  |
|  |  |  | chi2=0.03 | chi2=0.25 | chi2=0.18 | chi2=0.22 | chi2=0.03 |
|  |  |  | p=0.86776 (P) | p=0.61891 | p=0.67099 | p=0.64176 | p=0.87027 |
